# Molecular evolution and diversification of the Argonaute family of proteins in plants

**DOI:** 10.1186/s12870-014-0364-6

**Published:** 2015-01-28

**Authors:** Ravi K Singh, Klaus Gase, Ian T Baldwin, Shree P Pandey

**Affiliations:** Department of Biological Sciences, Indian Institute of Science Education and Research Kolkata, Mohanpur Campus, Mohanpur, Nadia, 741246 West Bengal India; Department of Molecular Ecology, Max Planck Institute for Chemical Ecology, Jena, 07745 Germany

**Keywords:** Argonaute, miRNA, Plants, *Nicotiana attenuata*, Herbivory, Evolution, Small-RNA

## Abstract

**Background:**

Argonaute (AGO) proteins form the core of the RNA-induced silencing complex, a central component of the smRNA machinery. Although reported from several plant species, little is known about their evolution. Moreover, these genes have not yet been cloned from the ecological model plant, *Nicotiana attenuata,* in which the smRNA machinery is known to mediate important ecological traits.

**Results:**

Here, we not only identify 11 AGOs in *N. attenuata*, we further annotate 133 genes in 17 plant species, previously not annotated in the Phytozome database, to increase the number of plant AGOs to 263 genes from 37 plant species. We report the phylogenetic classification, expansion, and diversification of AGOs in the plant kingdom, which resulted in the following hypothesis about their evolutionary history: an ancestral AGO underwent duplication events after the divergence of unicellular green algae, giving rise to four major classes with subsequent gains/losses during the radiation of higher plants, resulting in the large number of extant AGOs. Class-specific signatures in the RNA-binding and catalytic domains, which may contribute to the functional diversity of plant AGOs, as well as context-dependent changes in sequence and domain architecture that may have consequences for gene function were found.

**Conclusions:**

Together, the results demonstrate that the evolution of AGOs has been a dynamic process producing the signatures of functional diversification in the smRNA pathways of higher plants.

**Electronic supplementary material:**

The online version of this article (doi:10.1186/s12870-014-0364-6) contains supplementary material, which is available to authorized users.

## Background

Small-RNA (smRNA)-mediated pathways form a fundamental layer of the transcriptional and post-transcriptional gene regulatory network whose complexity is not fully realized [1-4]. The core of this process of RNA interference (RNAi) involves the formation of the RNA-induced silencing complex (RISC) with the help of two major factors. The first factor is the growing class of 18-40 nucleotide (nt) non-coding smRNAs, such as microRNAs (miRNAs), and small-interfering RNAs (siRNAs) [1,5]. These smRNAs act as sequence specific guides for the second component, the AGOs [4,6,7]. AGOs have been implicated as proteins essential in the gene regulatory mechanisms fundamental to developmental and cellular processes such as mRNA stability/degradation, protein synthesis, and genomic integrity [4,6,8]. The AGO proteins have characteristically four domains: an N-terminal domain, the PAZ domain, the MID domain and the PIWI domain [4,9]. The C-terminus of the protein harbors the MID-PIWI lobes. MID-domains have a ‘nucleotide specificity loop’ that is involved in recognition and binding of the 5’phosphate of smRNAs, whereas the PIWI domains harbor the capacity to slice due to their characteristic catalytic tetrad, 'D-E-D-H/D', at the active site [4,9,10]. The 2-nt overhang at the 3' end of miRNAs is recognized by and anchored in the groove of the hydrophilic cleft of the PAZ domain [10,11]. The N-terminus probably facilitates the separation of smRNA-mRNA duplex as well as may regulate the slicer activity on the target mRNA by interacting with the 3’ end of the guide RNA, as recently shown for *Drosophila melanogaster* AGOs [12].

An AGO was originally discovered in forward genetic screens for genes involved in development in *Arabidopsis thaliana* [13]. Yet, little is known about the evolutionary diversification of these proteins across different plant genomes. In Eukaryotes, AGOs are broadly classified into two paralogous families: the AGO family, which have similarities to the founder member, AGO1 of the Arabidopsis, and the PIWI-like proteins, related to *D. melanogaster* ‘P-element induced wimpy testis’ (PIWI) proteins [4]. While plants have been reported to encode only the AGO-like paralogs, animal genomes harbor representatives of both groups, whereas Amoebozoa are reported to have only PIWI-like genes [4]. A third group of AGOs is specific to *Caenorhabditis elegans* [14]. These findings suggest that both the families have experienced lineage-specific losses [4]. The number of AGO genes varies from 1 (*Schizosaccharomyces pombe* ) to 27 (*C. elegans*; [7,14]); the AGO genes seem to have undergone multiple gene duplication events, but mainly in plant genomes [7]. Plants such as *Chlamydomonas reinhardtii* and *Physcomitrella patens* (‘lower plants’) contain 4 and 6 members, respectively [15,16], whereas ‘higher plants’ such as *Oryza sativa* (OsAGOs) and *A. thaliana* (AtAGOs) contain 18 and 10 members, respectively [1,2]. In a phylogenetic classification based on protein similarity, 10 AtAGOs were distributed into 3 phylogenetic clades [4,7], whereas 18 AGO genes of *O. sativa* were divided into 4 clades [7,17]. However a comprehensive classification of plant AGOs is still missing.

In plants, duplication events may have resulted in functional diversification of AGOs as well as their biochemical activities [7,18]. For instance, of the 10 AGOs in Arabidopsis, catalytic activities have been demonstrated for only AGO1, AGO2, AGO4, AGO7 and AGO10 [19-21]. AtAGO1 and AtAGO10 preferentially bind to smRNAs with a 5'-Uridine (U), whereas AtAGO2, AtAGO4, AtAGO8 and AtAGO9 prefer smRNAs having a 5'-Adenine (A) [22-24], while AtAGO5 has greater affinity to 5'-Cytosine (C) containing smRNA [24]. AtAGO10 preferentially binds to smRNAs of 21-nt length, whereas AtAGO4, AtAGO6 and AtAGO9 bind to 24-nt endogenous smRNAs [23,24]. AtAGO1 binds to miRNAs that are processed by DCL1 and ta-siRNA processed by DCL4 [23,24]. Furthermore, 82% of smRNAs that associate with AtAGO1 are miRNAs [23], whereas, approximately 11, 2 and 5% of miRNAs are associated with AtAGO2, AtAGO4 and AtAGO5, respectively [23]. AtAGO4 has preferences for miRNAs that are processed by DCL3 [25]. AtAGO4, AtAGO6 and AtAGO9 participate in the RNA-directed DNA methylation pathway [18], whereas AtAGO1 and AtAGO4 play a role in virus resistance [26,27]. The large number of AGO genes suggests that the smRNA regulatory pathways in plants has undergone substantial diversification and evolution.

**Figure 1:**
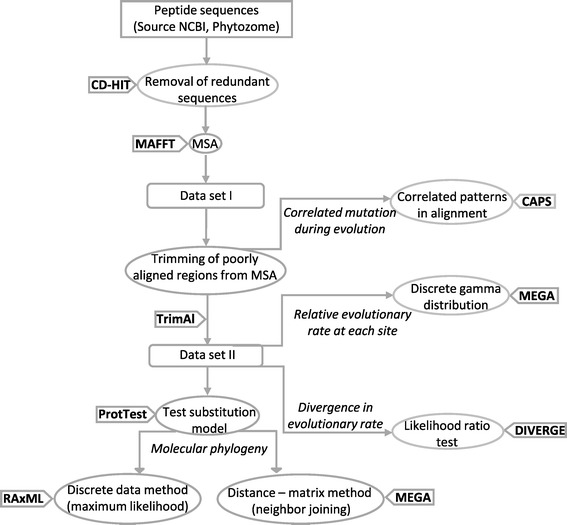
**Summary of sequential steps adapted to study evolution of AGOs in plants.** A total of 263 AGO sequences from 37 plant species were used in this analysis. Additionally, 5 AGOs from *T. castaneum,* and 1 each from Human (PDB code 4F3T) and *K. polysporus* (PDB code 4F1N) were also used (as out groups) to create the ‘AGO dataset I’, comprising a total of 270 AGO sequences. The list of AGOs for each species is available in Additional file 1. After MSA and trimming of poorly aligned regions or large gaps, ‘AGO dataset II’ was generated to contain 270 sequences (rows) and 620 positions (columns; Additional file 2).

Other than in Arabidopsis, AGOs have been reported in other plant species such as rice, maize, and tomato. These genes, however, have yet been identified in the ecological model plant *Nicotiana attenuata* in which the smRNA machinery is known to mediate important ecological traits such as herbivore resistance, competitive ability and UV-B tolerance [28-32]. Here, we identify the AGO family of genes in *N. attenuata* (NaAGO), a plant that grows in agricultural primordial niches and is an important model system for the study of plant-herbivore interactions. Further, we investigated the occurrence of AGO proteins in 17 plant species to identify 133 new AGO proteins in plants. Using integrative biology approach (Figure 1) involving molecular phylogenies, consensus sequence comparisons, signature determination, substitution rate estimations and divergence analysis, we propose a model for the evolutionary history of the AGO family of proteins in plants.

## Results

### Data set assembly and identification of new AGOs in plant genomes

We began with the isolation of 11 unique, full length AGO gene homologs (Additional file 1) from *N. attenuata*. Putative NaAGOs showed high identity to 8 types of AtAGOs and were thus annotated accordingly as NaAGO1 (identity of >78% to AtAGO1), NaAGO2 (50.55%), NaAGO4 (>74%), NaAGO5 (59.98 %), NaAGO7 (68.95%), NaAGO8 (52.69 %), NaAGO9 (68.04%) and NaAGO10 (80.71%). For NaAGO1, three gene sequences shared >78% peptide identity with AtAGO1 and >87% peptide identity amongst each other; these were thus annotated as NaAGO1a, NaAGO1b and NaAGO1c. Similarly, two gene sequences of the NaAGO4 share 86.86 % peptide identity with each other and >74% identity with AtAGO4; these were named NaAGO4a and NaAGO4b*.* However, we were not able to identify AtAGO3 and 6 homologs in *N. attenuata*. Further, we mined the sequence data of 17 plant species to identify and similarly annotate 133 full length AGOs. These had not been previously annotated as AGOs (Additional file 1). Altogether, 263 protein sequences were used from 37 plant species (Additional files 1 and 2). Additionally, 5 AGO sequences from *Tribolium castaneum,* 32 AGO sequences (including AGO1 and AGO2) from insects and early branching animals (e.g. sponges, cnideria), and one each of HsAGO2 (PDB code: 4F3T) and KpAGO (PDB code: 4F1N) (for a total of 302 sequences from 66 species; Additional files 1; detailed in methods section) were used as the out-group in this analysis.

### Phylogenetic classification and evolutionary expansion of plant AGOs

**Figure 2:**
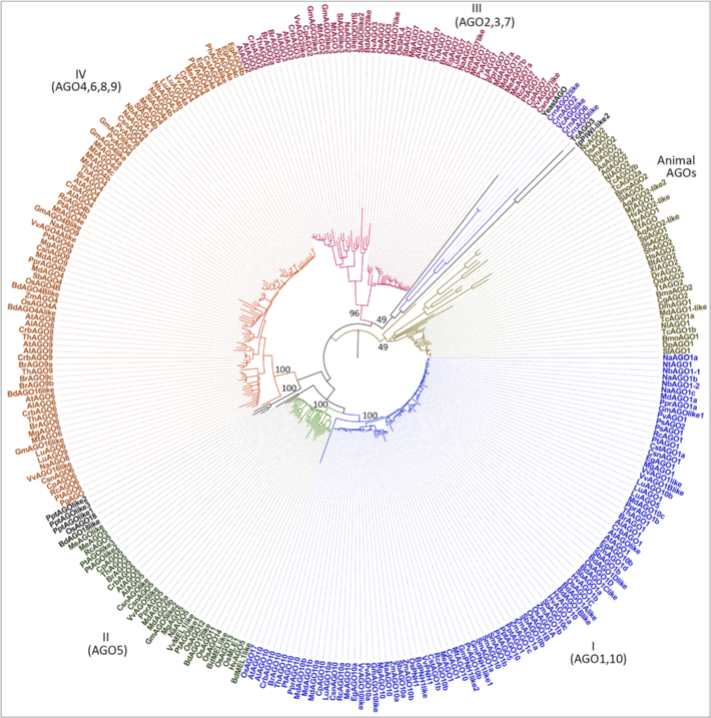
**Neighbor joining (NJ) based phylogenetic analysis of AGOs.** MEGA 5.2 was used to run the NJ analyses. 39 non-plant AGOs were used to determine the root. Clade robustness was assessed with 100 bootstrap replicates.

During evolution, AGO genes have formed an expanding family across different lineages [1,7]. To determine the evolutionary relatedness of plant AGOs, we reconstructed their phylogeny to evaluate their evolutionary patterns (Figure 1). In order to increase the confidence in the root we included 39 non-plant AGO sequences in the phylogenetic analysis. Plant AGO family proved monophyletic and the phylogenetic tree continued to consist of four major classes/clades (Figure 2, Additional files 1). Both the Neighbor Joining (NJ) and the Maximum Likelihood (ML) approaches were used to reconstruct the phylogeny of plant AGOs and both produced similar tree topologies and phylogenetic distributions into four classes/clades (Additional file 3). Homologs of AGO1 and AGO10 were clustered together (Clade I); similarly homologs of AGOs 2, 3 and 7 formed a clade (Clade III). Likewise, homologs of AGOs 4, 6, 8 and 9 formed the largest cluster (Clade IV), whereas AGO5 homologs formed an independent group (Clade II; Figure 2).

**Figure 3:**
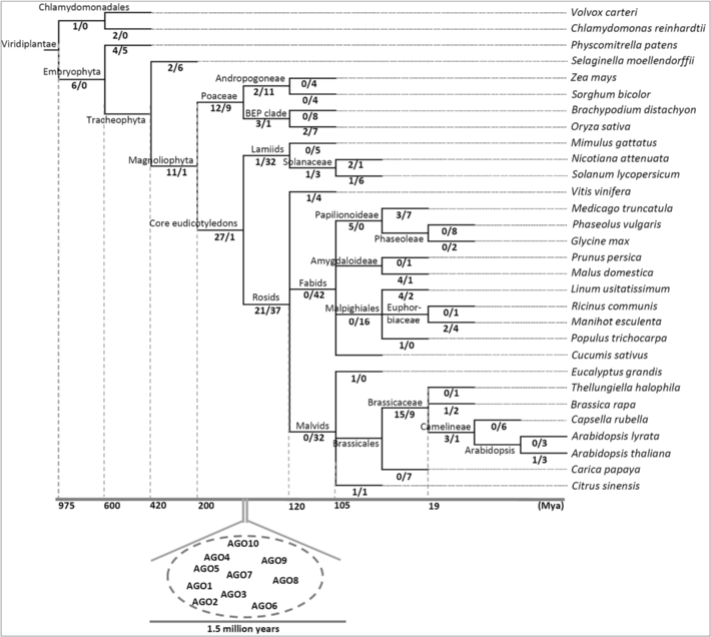
**Expansion of AGOs during plant evolution.** AGO gene family tree was reconciled with the completely sequenced species tree to identify gain and loss events in each lineage during evolution. The proportions of gains (numerators) versus losses events (denominators) for AGO genes are shown on each of the branches. Lower panel indicates the tentative time of appearance of different members of the AGO family in plants.

From the analysis of AGO gene expansion and loss (detailed in method section), it was observed that AGOs might have undergone between 133-143 duplication and 272-299 loss events (Figure 3, Additional file 4). We altered the alignment and alignment processing parameters to test the robustness of our analysis. When L-INSI in MAFFT and ‘Automated I’ in TrimAl were used, 140 duplication and 299 loss events were obtained; when the parameters were changed to L-INSI (MAFFT) and user defined parameters in TrimAl (detailed in methods section), 133 duplication and 294 loss events were recorded. Similarly, when Auto options were used for both MAFFT and TrimAl, 143 and 294 duplication and loss events were recorded respectively, whereas 137 duplication and 279 loss events were recorded when ‘Auto’ option in MAFFT and user-defined parameters for TrimAl were used. The reconciliation of species tree and AGO gene family tree (GFT) revealed that the AGO ancestor underwent at least five major duplication events early in its evolution, after the divergence of unicellular green algae, such as, Chlamydomonas and Volvox*,* but before the divergence of the Bryophytes. This probably gave rise to four distinct phylogenetic clades of AGOs (with strong statistical support with bootstrap values >90%; Figure 2, Additional file 3).

The AGO5 clade may have diverged before the divergence of higher plants, but after the evolution of multicellularity, suggesting a physiological role, possibly different from the ones regulating developmental processes (Figure 2, Additional file 3). Reconciliation of AGO GFT with the species tree showed that an ancestral AGO may have undergone >50 rounds of duplications by the time of the dicot-monocot divergence. (Figure 3, Additional file 4). Thus, diversification and duplication of AGOs could have coincided with the evolution of multicellularity, suggesting the relevance of AGOs and their associated smRNA pathways for developmental and adaptive programs.

The nodes of divergence between dicots and monocots apparent in all four AGO phylogenetic classes (Additional file 3) indicate that duplications were followed by speciation events (Additional file 4). For example, the relatively large number of AGO genes (containing all the four domains) in the Poaceae lineage, such as the 17 in *O. sativa* and the 14 in *Brachypodium distachyon* were noted (Additional file 1). These duplication events may have occurred in parallel with events leading to the loss of AGO family members during the evolution of Rosids and Lamiids (Additional file 4). Few such losses appeared to have occurred in the Brassicaceae and Solanaceae, for example, in which 10-11 members are found in *A. thaliana* and 11 AGOs in *N. attenuata* respectively (Figure 3, Additional files 1 and 4). In *N. attenuata*, homologs of AtAGO3 and AtAGO6 might have been lost while AGO1 and AGO4 were duplicated (Additional file 1). Duplicated copies of AGO4 are found in other Solanaceae taxa as well, such as in *N. benthamiana* [33] and *Solanum lycopersicum* (this study; Additional file 1).

The molecular clock test was performed to gain further insight into the relative timing of duplication and divergence events (Figure 3, Additional files 5 and 6). This analysis indicates that ancestral AGO gene may have required around 2 million years to duplicate four times after divergence from the unicellular green algae (Additional file 5). Clade IV may have been the first to diverge, followed by Clade III, Clade II and Clade I, respectively. It may have taken 0.5 million years for Clade I to evolve that now includes AGO1 and AGO10 homologs, while Clade IV may have required around 1.5 million years to evolve to include AGO4, AGO6, AGO8 and AGO9 homologs; AGO8 and AGO9 as its more recent descendants. Clade III most likely evolved around 1.25 million years and sub-diverged into two clusters, one comprising AGO7 and the other AGO2 and AGO3.

The phylogenetic tree (Figure 2, Additional file 3) reveals that AGO1 and AGO10 have orthologs in Selaginella and Physcomitrella*.* Interestingly, we found that of the 6 AGOs in Physcomitrella, the 3 previously unannotated AGO-like genes form a separate cluster (bootstrap value 100%). These AGOs may have diverged from the Clade IV lineage at a time comparable to the duplication of the ancestral AGO (Additional file 5), and thus may be orthologs of Class IV AGOs. Furthermore, homologs in unicellular forms, such as Chlamydomonas and Volvox*,* may have evolved independently from the multicellular lineages (Figure 2, Additional file 3). We observed that Chlamydomonas and Volvox AGOs harbor rudimentary forms of the PAZ domain but do not contain a distinct MID domain (Additional file 7). These results indicate that AGOs of higher plants are intricate and have substantially diverged from the lower, unicellular forms, potentially to facilitate the complex functions known to be regulated by smRNA pathways.

### Variability in signature residues of plant AGOs

**Figure 4:**
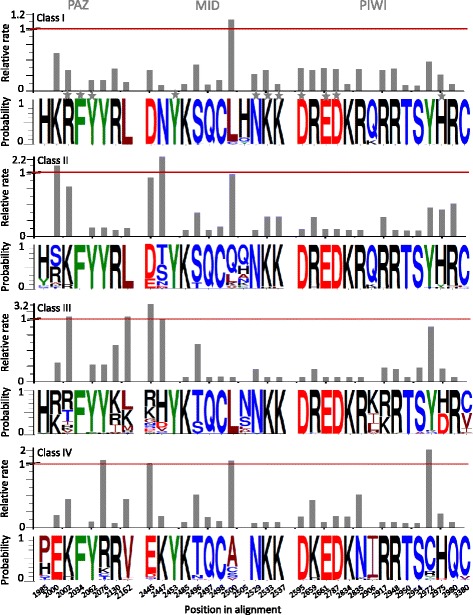
**Relative residue bias (probability; lower panel) and relative evolution rate (upper panel) at functionally important positions in the three domains of AGOs in the plant kingdom.** Relative frequency of each residue is represented by the height of the corresponding symbol. Height of the bar indicates the relative rate value for respective position. The positions marked with stars (in grey color) are the previously known signature residues.

Phylogenetic analysis indicates the presence of four clades/classes of AGOs and that these have been evolving differently. In addition, in plants, different AGOs are known to interact with different types of smRNAs (as described in the Background), wherein each residue of the 7nt region of smRNA, ‘the seed region’, sits in a narrow groove to interact with different residues of the MID-PIWI lobe of AGO proteins [10]. It is hypothesized that the sorting of different species of smRNAs to various AGOs [22,23] may depend on the conservation of these residues across various AGOs. Such functionally important residues may also be regarded as signatures of specific domains. Therefore, we attempted to define class-wise signature residues for each of the four classes as well as to re-examine the overarching architecture of AGO sequences in plant genomes. The N-terminal domain of AGOs is the most variable domain, whereas, 'R/K-F-Y', 'Y-N-K-K', 'D-E-D-H/D' have been regarded as the signatures of PAZ, MID and PIWI domains, respectively [7]. Upon examining the MSA of all the plant AGOs, we found 55 positions (column score >90) with highly conserved residues (Additional file 2). In parallel, we also examined the MSA of plant AGOs in each of the four classes independently to determine class-wise signature residues (Figure 4). We identified 8 sites in the PAZ domains, 12 sites in the MID domains and 15 sites in the PIWI domains that show conservation in the four classes AGOs. In the MID domain, residues ‘K’, ‘Q’ and ‘C’ (alignment position 2485, 2497 and 2498, respectively), thought to directly bind to the 5’-phosphate of smRNAs [10], are conserved in all four classes (Figure 4). Similarly, ‘K and ‘S’ (alignment position 2834 and 2954) of PIWI domain are conserved in all the four classes (Figure 4).

Results of the MSA indicated that residue ‘R’, the popularly regarded signature of the PAZ domain ('R-F-Y', alignment positions 2002, 2034 and 2062, respectively), are only conserved in Class I AGOs (AGOs 1 and 10). ‘R’ has been largely replaced by ‘K’ (Figure 4) in AGO of Class II (AGO5) and IV (AGOs 4, 6, 8 and 9), whereas the consensus residue could not be determined for this position (Figure 4) in the PAZ domain of Class III AGOs (AGOs 2, 3 and 7). Further, ‘H’ at the alignment position 1985 (Figure 4) in the PAZ domain, thought to be important in the recognition of the 3’ ends of smRNAs [10], is conserved only in Classes I-III; conserved residues were not found at this position in the PAZ domain of Class IV genes (Figure 4).

Another residue relevant to the interaction of AGO with the 5’-phosphate of the smRNA in the ‘nucleotide specificity loop’ of the MID domain is 'T526' (in HsAGO2), which corresponds to alignment position 2447 in plants (Additional file 2). Classes I and IV genes harbor a conserved ‘N’ and ‘K’ respectively, whereas there is no consensus in Classes II and III at this position. Studies of HsAGO2 [10] suggest that the first oxygen atom of the 5'-phosphate of smRNAs also interacts with side-chain residue of ‘R812’ in the PIWI domain. Position 2980 corresponds to ‘R812’, and harbors a conserved ‘R’ in Classes I-III genes, while in the Class IV genes, PIWI has a conserved ‘Q’ instead (Figure 4). In the crystal structure of HsAGO2 in a complex with miR-20a, the 2^nd^ nucleotide of smRNA interacts with ‘Q548’ of the MID domain and ‘Q757’ of the PIWI domain [10]. These residues correspond to positions 2500 and 2906 in MSA. An ‘L’ is present at position 2500 in Classes I and III, whereas Classes II and IV are highly variable, with ‘Q’ and ‘A’ being over-represented in these two classes respectively (Figure 4). The 5^th^ nucleotide interacts with 'S798' and 'Y804' from the PIWI domain in HsAGO2 [10]. The first corresponding sites in plant AGOs contain 'S' (MSA position 2954) in all four classes, the second site harbors 'Y' (MSA position 2972) in Class I-III whereas 'C' is the over-represented residue in Class IV.

The ‘D-E-D-H/D’ signature has been associated with the catalytic activity of the PIWI domain [4,7]. The ‘D-E-D-H’ signature is apparent in Classes I, II, IV (and half of the Class III) of plant AGOs, whereas the D-E-D-D signature is present in AGO2 and AGO3 (Class III PIWIs; Figure 4, Additional file 2). In general, most of the functionally important sites of Class-I AGOs are conserved, while the converse seems true for Class-III AGOs (Figure 4).

Since the phylogenetic analysis indicates that the AGOs of unicellular forms such as Chlamydomonas and Volvox are highly divergent and evolved independently of those of the multicellular forms, we further investigated the occurrence of the above-mentioned residue signatures and predicted functionally important sites (Additional file 8). We found a high diversity across many important sites (Additional file 8). Similarly, the three *Physcomitrella* AGOs also have unique residues compared to AGOs in other lineages (Additional file 8).

Such patterns of occurrence of functionally important residues may have consequences for smRNA recruitment, their biochemical activities and the roles of AGOs in diverse physiological processes in both unicellular and multicellular life-forms. Indeed, our homology modeling and RNA docking studies clearly pointed towards differences in seed recognition and catalytic region of the four classes of AGOs (Additional file 9).

### Evolution of AGO sequences

**Figure 5:**
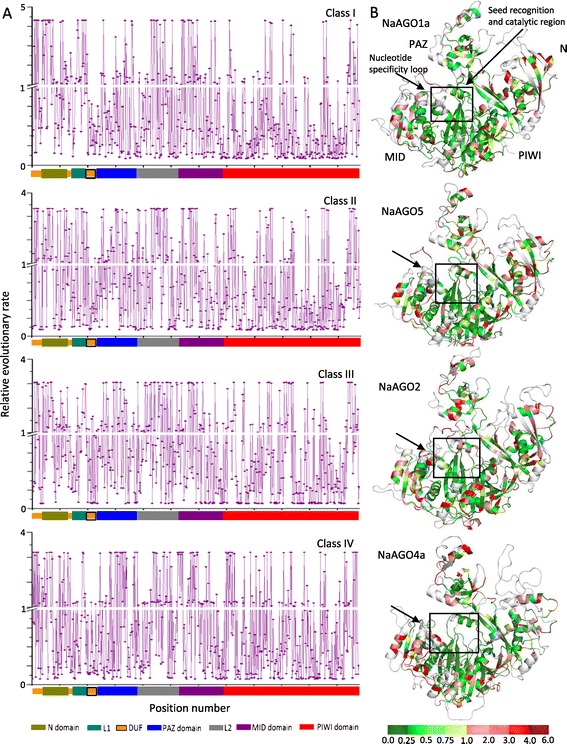
**Relative evolutionary rate for each site across four plant AGOs classes. (A)** shows site specific relative evolutionary rates of AGOs across classes I-IV. Position-by-position (maximum likelihood) relative evolutionary rates are estimated under the JTT amino acid substitution model. Mean (relative) evolutionary rates are scaled such that the average evolutionary rate across all sites is 1. X-axis represent the positions of residues (620 residues) of the ‘AGO dataset II’ along the N-terminal, PAZ, MID and PIWI domains in AGO sequence. Y-axis shows the relative evolutionary rate. Sites showing rates <1 are evolving slower than average and those with rates >1 are evolving faster than average. **(B)** Threaded structures of NaAGO1a, NaAGO5, NaAGO2 and NaAGO4a are modeled as representatives of Classes I-IV respectively, and relative evolutionary rates are mapped on to these structures. Sites with green color represent slow evolving sites (rates <1) and those with red color represent fast evolving sites (rates >1). Different colors in the color bar represent the different rate values.

We next determined the 'position-by-position' ML-based relative evolutionary rates using a gamma (γ)-distribution based best substitution model. Of the total 620 sites in ‘AGO dataset II’ (Figure 1, Additional files 2 and 10), 218 sites have a relative rate <1 whereas 69 sites have relative rates >1 in all four classes (Additional file 10: Table S3A). Relatively small ML values of γ- shape parameter were observed for Class I (0.5881; Additional file 10: Table S3B), indicating that the majority of sites (405) in Class I AGOs (Additional file 10: Table S3B) are evolving at slow relative rates. These sites are more frequently found in the MID and PIWI domains (Figure 5). On the other hand, Class III AGOs show a large ML value of the γ- shape parameter (1.0174; Additional file 10: Table S3B), indicating that less number of sites (361 as compared to 405 for example in Class I) are evolving at slow relative rates (Figure 5, Additional file 10: Table S3B).

Residues involved in substrate recognition and catalysis show low relative rates of evolution (Figures 4 and 5), indicating such residues are conserved during the course of evolution. For instance, the ‘D-E-D-D/H’ signature involved in catalytic activity of the PIWI domain has low relative rates across all the four classes of AGOs. Overall, the seed recognizing MID-PIWI lobe of Classes I and II show a low relative rate (slow evolving; Figure 5). Moreover, other regions putatively involved in seed recognition and the nucleotide specificity loop show a low relative evolutionary rate in Class I AGOs as compared to other classes (Figure 5). For certain sites, substitution of residues along with variability in relative rates was noticed between different classes. For instance, at position 2000, located near the seed recognition pocket and implicated in the 3' overhang recognition of smRNA [10], substitution of K in Class I to E in Class IV was observed; both the residues are evolving at slow rates (Figure 4). Such changes may explain the capacity of AGO proteins to sort and load smRNAs with specific residues at their termini [23]. On the other hand, it was interesting to note that the N-terminal and the PAZ domains have several sites with high relative rates (fast evolving) across all four classes of AGOs (Figure 5).

These observations suggested the possibility that different classes of AGOs undergo site-specific rate shifts. We performed the likelihood ratio test by calculating the coefficient of Type I (θ_I_) divergence and the posterior probability (PP) of a shift in substitution rate (Additional file 11). Rejection of the null hypothesis (θ_I_ > 0) indicates that after duplication, selection constraints may have altered many sites differently in different classes (thus shifts in substitution rates in different classes; θ_I_ values of 0.2814-0.6509 for pairwise comparisons; Additional file 11: Table S4A). Hence, as expected, large variations in site-specific profiles of PP among different classes were observed (Additional file 11: Table S4B). Maximum shifts were observed between Classes I and IV (Additional files 11: Table S4B, and Additional file 12). Also, the functional branch lengths (b_F_) of Class IV and Class III were nearly two times greater than the branch length of Class I and Class II (*p* <0.05; Additional file 13). Such results point to different evolutionary histories of different classes of AGOs that may have resulted in different structural and functional properties; Class I AGOs may have diverged functionally more than Class IV AGOs.

### Context-dependent coevolution of amino acid residue

**Figure 6:**
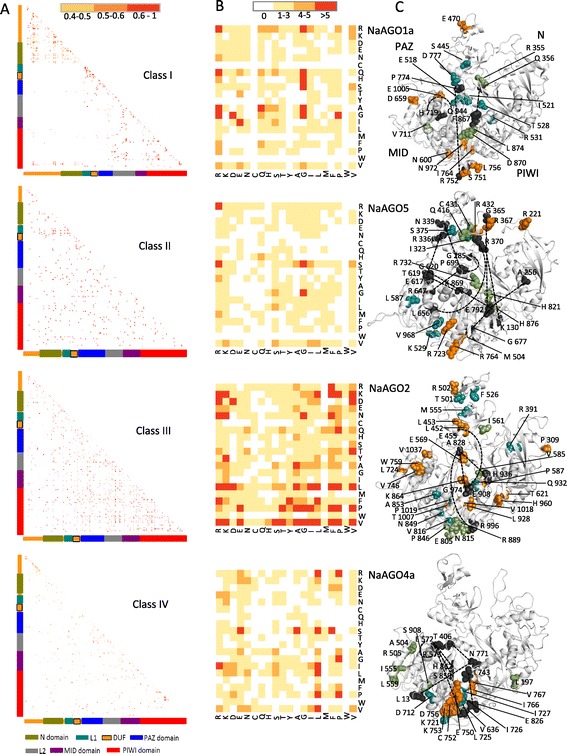
**CAPS 2.0 analysis of coevolving sites in plant AGOs. (A)** shows heatmaps of coevolving sites in the four classes of plant AGOs. Coevolving pairs showing correlation coefficient of ≥0.5 are plotted. **(B)** is the color-coded representation of the coevolution frequency matrix of 20 amino acid pairs in NaAGO1a, NaAGO5, NaAGO2 and NaAGO4a, the representatives of four classes respectively. **(C)** Threaded structures of NaAGO1a, NaAGO5, NaAGO2 and NaAGO4a show the position and arrangement of top coevolving groups in the classes I-IV respectively (orange, green and blue colored; residues in black represent other functionally important coevolving sites as described in Results and Discussion). Residues coded with same color show the correlation with each other in evolutionary context.

The evolution of protein residues is frequently context-dependent in that substitutions at a given site are affected by local structure, residues at the other sites, and related functions. Such context-dependent substitutions result in co-evolution of amino-acid residues that have implications for protein structure and function. We uncovered coevolving residues in plant AGOs by using Pearson correlation coefficient (r) as implemented in CAPS 2.0 (coevolution analysis using protein sequences) algorithm [34]. Only co-evolving sites with r ≥ 0.5 were considered significant (Figure 6, Additional file 14: Table S5A). Class III AGOs accounted for largest number of coevolving residues (Figure 6A, Additional file 14: Table S5A). Strong correlation of r > 0.9 was observed between the sites coevolving in the PAZ domain and PIWI domain of Class III AGOs (Figure 6A, Additional file 14: Table S5A). Four classes of AGOs displayed heterogenous coevolving groups of residues that are of different sizes. In Class III AGOs, PIWI domains displayed the largest number of coevolving residues (Figure 6A, Additional file 14: Table S5B). In general, the amino acid residue 'R' is the most frequently correlating residue in Class I and II, while residue ‘L’ is found most frequently correlating in Classes III and IV (Figure 6B, Additional file 14: Table S5C). In Class I, 'G' is the second most frequent residue that is significantly correlated mainly to 'G', 'Q', ‘R’ and 'H'. In Class II, ‘G’ is again the second most frequent residue that instead significantly correlates to 'V', 'S', ‘E’, 'K' and 'R' (Figure 6B, Additional file 14: Table S5C). In Class III and IV, ‘P’ is the second most frequent residue that significantly correlates frequently to ‘V’, ‘Q’ and ‘F’, and to ‘P’, ‘G’ and 'R' respectively (Figure 6B, Additional file 14: Table S5C).

Correlation patterns in the context of specific residues at a site in the sequence were observed. For instance, position 2002 (in MSA) in the PAZ domain (may play a role in wedging 14^th^ and 15^th^ nucleotide of loaded RNA duplex [10]), is overrepresented by the residues ‘R’ in Classes I and III, and ‘K’ in Classes II and IV AGOs respectively (Figure 4). This 'K' is highly correlated with two other residues in Classes II and IV (Figure 6C, Additional file 14: Table S5A). On the other hand, position 2002 in Classes I and III do not show any significant correlation coefficient with other residues in the protein. Similarly, the ‘H’ at position 2505 (Figure 4) in the MID domain of Class I AGO is highly correlated to residue ‘Q’ at position 2906 in PIWI domain (Figure 6C, Additional file 14: Table S5A). Residue corresponding to position 2505 (Figure 4) could bind to phosphate of 2^nd^ nucleotide of smRNA, directing the 1^st^ nucleotide into a deep binding pocket at the interface between MID and PIWI domain, whereas Q, corresponding to postion 2906 may coordinate with N2 and N3 on the minor groove side of the G5 base at seed sequence of smRNA [10] . In other classes, where ‘H’ is replaced, no significant correlation is observed. In HsAGO, ‘R’ corresponding to 2835 in the PIWI domain (MSA; Additional file 2) stacks between the U9 and U10 of miRNAs to result in a major kink [10]. In Classes I, II and III residues ‘R’ is conserved at position 2835 (Figure 4) and do not show any correlation with other residues, whereas in Class IV AGOs, this position is overrepresented by ‘N’ and shows significant correlation to two other residues (Figure 6C, Additional file 14: Table S5A).

Diverse correlation patterns were observed in the ‘nucleotide specificity loop’ across the four classes of AGOs (Figure 6). None of the five residues of nucleotide specificity loop of Class I AGO showed any significant correlation. The 5’-end of the smRNAs interact with peptide backbone of the HsAGO residues [10] corresponding to positions 2445 and 2447 (Additional file 2). ‘T’, as in HsAGO, was overrepresented only in Class II (AGO5) and correlated to residues in PIWI and PAZ domains (Figure 6C). On the other hand, in Class IV, ‘E’ (MID domain; position 2445; Figure 4) correlates with ‘R’ in MID domain and ‘V’, ‘S’ and ‘H’ in PIWI domain (positions 2446, 2567, 2972 and 2975 respectively; Figure 4 and 6C; Additional file 14: Table S5A). Such class specific coevolving residues may influence the functional diversification of AGOs.

## Discussion

Several differences in smRNA processing and mode of action have been noted between plants and animals [1,3,35]. In addition, no significant homologies have been found in miRNAs of plants and animals, plants and green algae, or between animals and sponges [1,3,36,37]. This indicates that the smRNA pathways may have evolved independently in the different lineages of life. AGO proteins form the core of the smRNA-mediated regulatory mechanisms and thus are *bonafide* candidates for studying the evolution of smRNA pathways. Here we have reconstructed a comprehensive phylogeny of plant AGO proteins and examined their evolution. Based on this analysis of 302 AGO genes from 66 species, plant AGOs can be divided into four phylogenetic clades/classes. These results suggest that early speciation events separated the AGOs in unicellular and multicellular organisms, wherein AGOs expanded independently to evolve complex domain structures. An ancestral AGO gene may have undergone approximately five duplication events during the time of divergence of green algae and mosses. The AGO family may have further expanded with the emergence of monocot and dicot lineages in plants. Later speciation events may have resulted in species-specific gains or losses of some members.

The smRNA-mediated interaction is biochemically based on the principle of recognition and loading of smRNAs onto the AGOs to form an RNA-protein complex. This complex targets complementary mRNAs and regulates protein synthesis. Diverse pools of smRNAs occur in plant cells that exploit this elegant principle of RNA recognition and cleavage to fine-tune gene expression. Plants produce a large diversity of smRNAs (for instance, miRNAs, tasiRNAs, lsiRNAs, natsiRNAs; [1] that vary in their length (21 nt, 22 nt, 24 nt, and others; [1] and in a preferred base at the 5’ end of smRNAs (e.g. U, A or C; [35]. The particular type of smRNAs that is recruited for executing a particular biochemical RNAi depends on the interaction of the specific smRNA type with an AGO partner [23,35]. For instance, virus and sense transgene silencing requires the recruitment of 21-22 nt smRNAs onto Class I AGO (AGO1); DNA methylation/chromatin modifications require the association of 24 nt smRNAs onto Class IV proteins (AGOs 4, 6, 9), whereas 21 nt miRNAs recruited onto AGO1 results in mRNA cleavage in plants [23]. The organization of the signatures in RNA-recognition and catalytic domains (PAZ, MID and PIWI) of AGOs play a crucial role in recognizing various forms of smRNAs (Additional file 9). Such variations in RNA-interacting domains of AGOs (such as the conservation of R in the signature RFY residues occur only in Class I proteins, the variation of DDH in Class III; conservation of 'N' in the 'nucleotide specificity loop' in the MID domain of only Class I AGOs; Figure 4) may influence substrate recruitment as well as its biochemical fate (such as mRNA degradation, translation inhibition or DNA methylation/chromatin modifications) of the smRNA-target interaction [22,23,35].

Here we provide a comprehensive evaluation of plant AGOs: we have not only annotated 133 AGOs in 17 plant species (Additional file 1), we have also cloned 11 AGO genes from the wild tobacco plant, *N. attenuata* (Additional file 1), a well-studied model system for plant-insect interactions and adaptive plasticity. We have shown earlier that *N. attenuata* harbors at least three functional RdRs [29-31,38] and four DCLs [32]; information on AGO protein in *N. attenuata* had been totally missing. A detailed investigation on the elucidation of function of these 11 NaAGOs could reveal the effector molecules of a unique herbivory-elicited smRNA pathway.

It is evident that many of the functionally important residues are coevolving between the four groups of AGOs. For instance, the residue ‘P’ (position 2605; Additional file 2) is conserved in Classes I, II and IV, and correlates with residues R in Classes I, II, and III (Additional file 14: Table S5A). Similarly, position 2800 correlates with only a single polar residue in Classes I, III and IV whereas in Class II, this position correlates with seven residues that may be polar or nonpolar, indicating that the selection pressure is higher at this site in Class II (AGO5) as compared to other classes (Additional file 14: Table S5A). Coevolution patterns may form the basis of specialization of AGOs for differential sorting of smRNAs [22,23]; the 5’end of the incoming smRNA interacts with the peptide backbone of the AGOs. The conformational variability of the residues in the nucleotide specificity loop (Figure 5B and 6C) would make the AGOs selective for specific smRNAs and thus helping them sort different 5’ nucleotides of an incoming RNA [22,23].

Such specific variations in residues may lead to the functional specialization of AGO proteins. Indeed, specificity in the physiological functions of AGOs has been noticed in Arabidopsis. For instance, AGO4 has been implicated in anti-bacterial defense, whereas AGO1 is implicated in anti-viral response mechanisms [39]. It is noteworthy that although multi-member AGO families have been predicted computationally in several plants, many AGOs in plants (for example in rice and tomato) have not been experimentally confirmed; information on their physiological roles is thus meager. Here we have experimentally determined the full length sequences of 11 *N. attenuata* AGOs. Extensive 'loss-of-function' analyses of these 11 NaAGOs is planned for future investigations.

## Conclusions

The evolution of AGO proteins highly coincides with the evolution of multicellular forms of plants, indicating that smRNAs may have played crucial roles in their development and adaptation. Such an evolutionary pattern of duplication of AGOs also coincides with the evolution of other components of smRNA pathway as recently reported for animal and plant Dicers [40]. Our analysis is consistent with the conclusion that the AGO-mediated recognition of RNA and its regulation is a highly dynamic phenomenon across evolutionary time scales.

## Methods

### Dataset assembly

Data was assembled in three stages: (1) cloning of 11 *N. attenuata* AGOs, (2) mining of novel AGOs and their annotation in plant genomes from public repository, and (3) analysis of already annotated AGO proteins in public databases and sequence repositories. To annotate AGOs, individual AGO transcript and protein sequences (protein sequences wherever not available, were generated by using ORF-finder [http://www.ncbi.nlm.nih.gov/projects/gorf/]) were compared to AtAGOs by using BLAST. While comparing AGO sequences to those of *A. thaliana,* we applied an ‘*e*’ value cutoff of ≤ 2e^−20^. For isolating *N. attenuata* AGOs, we used a strategy similar to one described earlier [32,41]. A library made from RNA of *M. sexta* OS-elicited leaf material was sequenced extensively. More than 100 sequenced clones were analyzed to identify full-length NaAGOs. Annotation for each of these NaAGOs was made on the basis of degree of similarity with *A. thaliana* AGOs1-10. Accession numbers of these NaAGOs have been listed in Additional file 1. The AGO nucleotide and protein sequence data have been deposited at DDBJ/EMBL/GenBank. Accession numbers of individual NaAGOs are listed in Additional file 1. The NaAGO sequences form a part of the Transcriptome Shotgun Assembly project that has been deposited at DDBJ/EMBL/GenBank under the accession GBGF00000000. The version described in this paper is the first version, GBGF01000000.

NCBI was the primary source of mining of the AGO peptide sequences. A total of 986 unique accession IDs with annotated AGO or AGO like sequences from different plant species available in NCBI (before 30^th^ January, 2012) were retrieved. Further, *A. thaliana* and *O. sativa* AGOs were confirmed from searches of the TAIR and TIGR database, respectively. ‘Tomato SBM database’ [42] was mined with TBLASTN using default parameters to search for AGO homologs in *Solanum lycopersicum*. Further, the Phytozome database (upto 8^th^ June, 2012; [43]) was mined with BLASTP using default parameter setting to search putative AGO homologs in each completely sequenced species. The CD-HIT program (http://weizhong-lab.ucsd.edu/cdhit_suite/cgi-bin/index.cgi?cmd=cd-hit) [44] with a 90% sequence identity cut-off was used to remove redundant peptide sequences from putative AGOs. Only full-length peptides containing all characteristic domains were retained for further analysis. We annotated each predicted AGO on the basis of their degree of similarity with *A. thaliana* AGOs.

MSA (Multiple sequence alignment) was performed with the help of MAFFT v. 7.130b [45,46] using the option of 'L-INS-I', a gap-opening penalty of 1.53 and an offset value of 0.123 as well as ‘Auto’ options. The variable length of many AGOs from different species and numerous poorly aligned regions from the MSA analysis could lead to phylogenetic artifacts. To improve our phylogenetic inferences, we removed the poorly aligned regions [47] to create a trimmed-down version, ‘AGO dataset II’, by using the program TrimAl v1.3 [48]. To test the robustness of analysis (determine how alignment and alignment processing parameters could affect evolutionary inferences e.g. duplication and loss events), TrimAl was used with 'automated I' option as well as user defined parameters: minimum percentage of position coverage = 10, gap threshold (fraction of positions without gaps in a column) = 0.9, similarity threshold (minimum level of residue similarity within a column) = 0.0. Four parameter combinations were tested to compute duplication/loss events: (i) L-INS-I (MAFFT) + Automated I (TrimAl), (ii) Auto (MAFFT) + Automated I (TrimAl), (iii) L-INS-1 (MAFFT) + User Defined (TrimAl), (iv) Auto (MAFFT) + User Defined (TrimAl). To test the best fit amino acid substitution model and parameter value for ‘AGO dataset II’ for the tree building analyses, we used ProtTest v 2.4 [49]. The Jones-Taylor-Thornton (JTT) model [50] with an estimated γ-distribution parameter (G) and the proportion of invariant sites (I) was the best fit model according to Akaike Information Criterion (AIC) frame work [51]. This suggests that sequences in the dataset ‘Plant AGO dataset II’ are closely related with discrete proportion of invariant sites [50].

The MSA of ‘AGO dataset I’ suggest diverse sequence features in lower groups such as algae. The positions of different domains in the lower plant AGOs were identified by sequence search of the SMART database (http://smart.embl-heidelberg.de/) [52,53].

### Phylogenetic analysis

Evolutionary relationship among different AGOs was determined using (i) the distance matrix based (NJ) and (ii) the discrete data based methods (ML). NJ analysis was performed with the help of MEGA 5.2 [54] using a JTT model and γ-distributed (G) rate among sites with parameter 1. We tested the homogeneity among lineages to measure the differences in evolutionary patterns for a pair of sequences. Site coverage was kept at 90%; in other words, only sites that have residues in more than 90% sequences were used in the analysis. Clade robustness was assessed with 100 bootstrap replicates. ML analysis was performed by RAxML v 7.2.8 [55]. RAxML analyses were conducted using the PROTCATJTT model with the optimization of substitution rates and site-specific evolutionary rates that are categorized into 25 distinct rate categories for greater computational efficiency [55]. The final tree was evaluated under the γ-distribution of rates and the robustness of clades was assessed with 100 bootstrap replicates.

### Expansion of AGOs

To infer the evolutionary history, the AGO gene family tree (GFT) was reconciled with species tree, generated by NCBI Taxonomy Browser (http://www.ncbi.nlm.nih.gov/Taxonomy/CommonTree/wwwcmt.cgi), using NOTUNG program [56], to identify gain and loss events of AGO genes during evolution. Of the 37 species, complete genome sequences of 30 species were available (Additional file 1). 248 AGOs from these 30 species were used to analyze gain or loss events. NOTUNG takes a gene family tree, a species tree and a bootstrap threshold as input to generate a gene duplication history as output. The proportion of ‘gain’ versus ‘loss’ events is shown on each branch. The numerators and denominators represent the number of gain and loss events respectively of AGO genes during course of evolution (Figure 3). The tentative time of appearance of different members of the AGO family during evolution was calculated using TIME TREE (www.timetree.org) [57,58].

### Molecular clock

To estimate the divergence time among the AGOs, the molecular clock test was performed by comparing the ML values for a given tree topology with molecular clock constraints to a topology, and without the molecular clock constraints under the JTT (+G + I) model [50] by using MEGA 5.2. Differences in evolutionary rates among sites were modeled using a discrete γ-(G) distribution that allowed for invariant (I) sites to exist. The null hypothesis of equal evolutionary rates throughout the tree was rejected at a 5% significance level.

### Structural modeling

HHpred server (http://toolkit.tuebingen.mpg.de/hhpred/) [59] was used to model the structure of NaAGO1a, NaAGO5, NaAGO2 and NaAGO4a as representatives AGO from classes I-IV respectively. HHpred detects remote protein homology and predicts structure from pairwise comparison of HMM profiles (Hidden Markov models) through various databases search, such as the PDB, SCOP, Pfam, SMART, COGs and CDD. It accepts a single query sequence or a multiple alignment as input and searches through local or global alignments and by scoring secondary structure similarities. HHpred produces pairwise query-template alignments, multiple alignments of the query with a set of templates selected from the search results, as well as 3D structural models that are calculated by the MODELLER from these alignments. Among all the plant AGOs known, the structure of only the MID domain of AtAGO1, 2 and 5 has been resolved to date [22] Human AGO2 (PDB code 4F3T, chain A) was the best template for most of the NaAGOs. ClusPro server (http://cluspro.bu.edu/login.php) [60] was used to dock the 20nt RNA (PDB code 4F3T, chain R) to all four NaAGOs.

### Relative evolutionary rate and divergence analysis

MEGA 5.2 [54] tool was used to estimate the position-by-position (ML) relative evolutionary rate under JTT + G + I amino acid substitution model. The estimate was performed on 90% conservation of sites with 4 rate categories [54]. Individual relative evolutionary rates at all the sites are scaled such that the average evolutionary rate across all positions equals to 1. Hence, positions showing relative rates <1 are more conserved than the average conservation of the sites in the alignment and vice versa. DIVERGE 2.0 [61] was used to identify the sites that show changes in the amino acids substitution rates among different classes (Type I divergence). A trimmed-down version containing 154 sequences of ‘Plant AGO dataset II’ was used as input to DIVERGE 2.0. A NJ tree for 154 sequences was generated with the help of MEGA 5.2 [54] using a JTT substitution model and clusters for respective classes were selected for pairwise comparison [61]. All paired class comparisons were used to calculate the Coefficient of Type I (θ_I_) divergence and the posterior probability of shift in substitution rate for sites. θ_I_ between different classes provides the statistical evidence for supporting the hypothesis of rate shift between different AGO classes (Additional file 10). A tree-like topology for AGO classes was generated that suggests about the functional distance among the classes.

### Intramolecular coevolution in AGOs

CAPS 2.0 (Coevolution analysis using protein sequences; http://bioinf.gen.tcd.ie/~faresm/software/software.html [34]) with default parameters was used to identify co-evolving amino acid site pairs (*e* and *k*) by measuring the correlated evolutionary variation at these sites. Evolutionary variation is measured using divergence time-corrected Blosum values for the transition between two amino acids at a particular site when comparing sequence '*i*' to sequence '*j*' at site '*e*' and '*k*' (*θ*_*ek*_)_*ij*._ The time is estimated as the mean number of substitutions per synonymous site between the two sequences being compared. Correlation of the mean variability is measured using the Pearson coefficient. Finally, the significance of the correlation coefficients is estimated by comparing the real correlation coefficients to the distribution of 10,000 randomly sampled correlation coefficients. For the coevolution analyses, a trimmed down version of AGO dataset I’ containing columns without gaps was used (Figure 1). Only coevolving sites showing correlation coefficient of ≥0.5 were considered.

### Voucher specimens

A voucher specimens is deposited at the Herbarium Haussknecht, Jena Germany with following details: *Nicotiana attenuata* Torrey ex Watson “Utah”, selfed for 30 generations; cultivation: Max-Planck-Institute for Chemical Ecology, Jena; origin: Washington County, Utah, USA; collected by Ian T. Baldwin, June 3, 1988; GPS coordinates: 37°19'36.26"N 113°57'53.05"W; leg: Tamara Krügel, 18.11.2014, JE. Whole plant including flowers and developing seed capsules was used for nucleic acids isolation.

### Deposition of phylogenetic trees/data in Treebase

All the phylogenetic trees and associated matrices have been deposited to the Treebase (http://treebase.org/treebase-web/home.html) with the study number 16716. The Treebase generated URL to access the deposition is http://treebase.org/treebase-web/search/study/summary.html?id=16716.

### Availability of supporting data

All the supporting data are included as additional files.

## Additional files

Additional file 1: Table S1. List of AGOs used in this study. Additional file 2: Figure S1. Multiple sequence alignment of ‘AGO dataset I’ by using the MAFFT v 7.130b with option ‘L-INS-I’. A total of 270 AGO sequences were aligned. In total, 55 columns are having column score >90 (score for conservation of physio-chemical properties of residues for a particular column, calculated by ClustalX; range: 0 - 100) are marked with star. The columns marked on the top of the MSA are the positions that were retained in the ‘plant AGO dataset II’ after trimming (the co-ordinates of positions are: 1256-1262, 1267-1282, 1444-1460, 1474-1490, 1598-1611, 1659-1677, 1747-1757, 1767-1772, 1778-1788, 1795-1800, 1844-1852, 1873-1879, 1941-1950, 1999-2013, 2058-2063, 2074-2082, 2086-2091, 2100-2107, 2111-2116, 2123-2134, 2157-2172, 2198-2206, 2213-2229, 2233-2246, 2282-2292, 2298-2315, 2333-2337, 2343-2347, 2400-2406, 2436-2449, 2484-2500, 2522-2537, 2548-2567, 2571-2577, 2592-2599, 2605-2622, 2639-2668, 2738-2743, 2766-2777, 2787-2815, 2822-2841, 2883-2905, 2914-2955, 2968-2980, 2986-2990, 3001-3007, 3312-3319 and 3331-3341. A total of 620 positions were retained). Additional file 3: Figure S2. Phylogenetic classification of plant AGOs. (A) Neighbor-Joining (NJ) phylogenetic tree of the plant AGOs (plant ‘AGO dataset II’). The brown colored nodes indicate duplication whereas the grey colored circles indicate the speciation followed by duplication nodes respectively. Clade robustness was assessed with 100 bootstrap replicates. (B) Maximum Likelihood (ML) phylogeny of all the AGOs in current study (‘plant AGO dataset II’). RAxML v 7.2.8 was used to run the ML analyses. The phylogeny was reconstructed by using the Jones-Taylor-Thornton (JTT) amino acid substitution model with 'G + I' parameter. Nodes show the AGOs from the respective plant. Additional file 4: Figure S3. Reconciled AGO gene family tree (GFT) with species tree showing details of duplication and loss events. The red internal node represents the duplication event while the blue node represents the speciation event. Grey color terminals are the lost nodes from respective taxonomic units. Additional file 5: Figure S4. Maximum likelihood (ML) tree of AGOs showing the approximate relative time of divergence of all the nodes using the molecular clock test on ‘plant AGO dataset II’. Small rectangular bars at the nodes in the tree represent the 90% confidence interval of the variance of the node height. The ‘scale’ (x-axis) is in million-years. Additional file 6: Table S2. Log-likelihood parameter of molecular clock test. Additional file 7: Figure S5. Schematic representation of domain architectures in AGOs from lower plant groups. The positions of different domains were identified by sequence search of the SMART database. Additional file 8: Figure S6. Comparative analysis of the signature residues at functionally important sites in different domains of AGOs of lower plant groups as compared to higher plant. Amino acids at sites corresponding to signature residues of AGOs of higher plants are not found/substituted in lower organisms such as Chlamydomonas. Additional file 9: Figure S7. Effect of variation in the residues in four classes on smRNA binding and stability with AGOs. Fig A, B, C and D show the interaction and position of the first oxygen atom of 5' P-U residue of RNA (PDB code 4F3T, chain R) in the lobe formed by MID-PIWI domain of NaAGO1a, NaAGO5, NaAGO2 and NaAGO4a, respectively. These docked structures were generated by using ClusPro server. The colored regions are the positions that interact and bind to the 5' P of smRNAs (red color on the PIWI domain and violet color on the MID domain). The first oxygen atom of the 5' P of smRNAs interacts with hydroxyl of 2^nd^ and 3^rd^ functionally important position of the MID domain and the 14^th^ functionally important position of the PIWI domain. In NaAGO1a, the distance between hydroxyl of asparagine (N) at 2^nd^ position and oxygen atom of the 5' P of smRNAs is estimated to be 7.3 Å, while in NaAGO4a, which has a lysine (K) at 2^nd^ position, this distance is estimated to be 9.6 Å. This difference may affect the binding capacity and functional specificity of plant AGOs. Additional file 10: Table S3. Relative evolutionary rate in plant AGOs sequences. (A) Site-specific relative evolutionary rate of AGOs across Classes. Orange: sites that show high relative rate (>1) across all classes. Sky blue: sites showing low relative rate (<1) across all classes. (B) Comparative summary of relative rate across four AGO Classes. Additional file 11: Table S4. Functional diversification in AGOs among four classes. (A) Coefficient of Type I (θ_I_) divergence (i.e functional divergence) and functional distance (dF) between different AGO Classes. The upper diagonal shows the 'θ_I_ ± SE' values for all pairwise combinations of AGOs Classes. The lower diagonal shows functional distance (dF) between respective Classes. (B) Site-specific profile of posterior probability (PP) responsible for the Class specific type I functional divergence among AGOs. The colored cells are the corresponding sites between AGO Classes indicating a strong probability of divergence (PP > 0.90). Additional file 12: Figure S8. The site-specific profile indicating critical amino acid positions measured by the posterior probability of being functionally divergent. X-axis represent the positions in ‘plant AGO dataset II’. Y-axis shows the posterior probability (PP). Result shows that there are 51 sites (PP > 0.9) that have strong probability of divergence between Class I and Class IV, whereas 36 and 38 sites (PP > 0.9) show strong probability of divergence between Class III and Class IV & Class II and IV, respectively. Additional file 13: Figure S9. Functional distance between four AGO classes in terms of tree-like topology. Class IV has longest branch length, indicating shift in largest number of sites from ancestor after duplication. Additional file 14: Table S5. Correlated amino acid pairs among different Classes. (A) Column ‘AA1’ and ‘AA2’ are the positions in the alignment of AGO sequences of respective Classes between which correlation coefficient (in the last column) were calculated by CAPS 2.0. (B) Summary of coevolving amino acid pairs in NaAGO1a, NaAGO5, NaAGO2 and NaAGO4a, representatives of Class I – IV, respectively. (C) Frequency of coevolving amino acid (AA) pairs in NaAGO1a, NaAGO5, NaAGO2 and NaAGO4a.
