# Supplementary material for: Molecular evolution and diversification of the Argonaute family of proteins in plants
Source: BMC Plant Biol. 2015 Jan 28;15:23. doi: 10.1186/s12870-014-0364-6 (PMC4318128; doi:10.1186/s12870-014-0364-6)
Supplement: Additional file 1: Table S1. — List of AGOs used in this study. [file 12870_2014_364_MOESM1_ESM.pdf]

Table S1. List of AGOs used in this study.
('√' = species where accession numbers were freshly annotation as AGOs. Sequences corresponding to these accession numbers were earlier not annotated as AGOs in the respective DB).

Species	Accession No.	Argonautes	Source	Status
Arabidopsis lyrata	EFH41660.1	AlAGO10	NCBI	
	EFH48230.1	AlAGO9		
	EFH50287.1	AlAGO8		
	EFH57134.1	AlAGO4		
	EFH57221.1	AlAGO5		
	EFH57513.1	AlAGO6		
	EFH64977.1	AlAGO7		
	EFH70108.1	AlAGO3		
	EFH70109.1	AlAGO2		
	EFH70370.1	AlAGO1		
Arabidopsis thaliana	AAF79718.1	AtAGOlike	NCBI / TAIR	
	At1g48410	AtAGO1		
	At1g31280	AtAGO2		
	At1g31290	AtAGO3		
	At2g27040	AtAGO4		
	At2g27880	AtAGO5		
	At2g32940	AtAGO6		
	At1g69440	AtAGO7		
	At5g21030	AtAGO8		
	At5g21150	AtAGO9		
	At5g43810	AtAGO10		
Brachypodium distachyon	XP_003559290.1	BdMEL1like	NCBI	
	XP_003559577.1	BdAGO12like		
	XP_003559728.1	BdAGO7like		
	XP_003560234.1	BdAGO18like		
	XP_003560616.1	BdPNH1like		
	XP_003561331.1	BdMELlike		
	XP_003563234.1	BdAGO1Dlike		
	XP_003567034.1	BdAGO4Alike		
	XP_003567769.1	BdAGO4Blike		
	XP_003570049.1	BdAGO1Alike		
	XP_003573171.1	BdAGO1Clike		
	XP_003576965.1	BdAGO16like		
	XP_003580340.1	BdAGO1Blike		
	XP_003581669.1	BdAGO2like		
Brassica rapa	Bra040815	BrAGO2b	Phytozome	√
	Bra020152	BrAGO9a		
	Bra002360	BrAGO9c		
	Bra002361	BrAGO9b		
	Bra033698	BrAGO10		
	Bra003999	BrAGO7		
	Bra011993	BrAGO5		
	Bra032254	BrAGO1		
	Bra034318	BrAGO4		
	Bra023172	BrAGO2a		
	Bra022918	BrAGO6		
Carica papaya	evm.model.supercontig_1.68	CpAGO7	Phytozome	√

	evm.model.supercontig_135.40<br>evm.model.supercontig_26.59<br>evm.model.supercontig_44.130<br>evm.model.supercontig_47.31<br>evm.model.supercontig_600.1<br>evm.model.supercontig_75.90	CpAGO2<br>CpAGO4<br>CpAGO10<br>CpAGO5<br>CpAGO6<br>CpAGO1		
Capsella rubella	Carubv10008180m<br>Carubv10008158m<br>Carubv10010881m<br>Carubv10022614m<br>Carubv10025461m<br>Carubv10022599m<br>Carubv10019715m<br>Carubv10002706m<br>Carubv10000166m<br>Carubv10025804m	CrbAGO2<br>CrbAGO1<br>CrbAGO3<br>CrbAGO6<br>CrbAGO5<br>CrbAGO4<br>CrbAGO7<br>CrbAGO8<br>CrbAGO9<br>CrbAGO10	Phytozome	√
Chlamydomonas reinhardtii	EDO99352.1<br>EDP01992.1<br>EDP01993.1<br>EDO99188.1	CrnAGOlike<br>CrnAGO2like<br>CrnAGO2<br>CrnAGO6	NCBI	
Citrus sinensis	orange1.1g002636m<br>orange1.1g003630m<br>orange1.1g001684m<br>orange1.1g001954m<br>orange1.1g002661m<br>orange1.1g002449m<br>orange1.1g001466m<br>orange1.1g002204m	CsnAGO9<br>CsnAGO5<br>CsnAGO7<br>CsnAGO10<br>CsnAGO6<br>CsnAGO4<br>CsnAGO1<br>CsnAGOlike	Phytozome	√
Cucumis sativus	Cucsa.082260.1<br>Cucsa.112480.1<br>Cucsa.152920.1	CstAGO1b<br>CstAGO1a<br>CstAGO4	Phytozome	√
Eucalyptus grandis	Eucgr.B03780.1<br>Eucgr.G02476.1<br>Eucgr.H00532.1<br>Eucgr.H00615.1<br>Eucgr.J00634.1<br>Eucgr.J00634.2<br>Eucgr.K02304.1	EgAGO4a<br>EgAGO7<br>EgAGO10a<br>EgAGO10b<br>EgAGO4c<br>EgAGO4b<br>EgAGO1	Phytozome	√
Glycine max	CAW52557.1<br>CAW52604.1<br>XP_003516290.1<br>XP_003519489.1<br>XP_003524004.1<br>XP_003526363.1<br>XP_003536500.1<br>XP_003540778.1<br>XP_003541581.1<br>XP_003547312.1<br>XP_003550835.1<br>XP_003556681.1	GmAGOlike1<br>GmAGOlike2<br>GmAGO7like<br>GmAGO4like<br>GmPNH1like1<br>GmAGO4Blike<br>GmAGO10like<br>GmAGO5like2<br>GmAGO16like<br>GmAGO2like1<br>GmPNH1like2<br>GmAGO2like2	NCBI	
Hordeum vulgare	BAK05938.1<br>BAK06654.1<br>BAJ95476.1<br>BAJ99496.1	HvAGO5<br>HvAGO1b<br>HvAGO3<br>HvAGO1a	NCBI	

	BAK04309.1	HvAGO10		
Lotus japonicus	ACN79520.1	LjAGO7	NCBI	
Linum usitatissimum	Lus10025537<br>Lus10006627<br>Lus10017983<br>Lus10015155<br>Lus10031331<br>Lus10037136<br>Lus10041978<br>Lus10035331<br>Lus10026750	LuAGO4b<br>LuAGO10a<br>LuAGO1<br>LuAGO9<br>LuAGO10b<br>LuAGO7<br>LuAGO5<br>LuAGO6<br>LuAGO4a	Phytozome	√
Manihot esculenta	cassava4.1_000932m<br>cassava4.1_000845m<br>cassava4.1_000940m<br>cassava4.1_001305m<br>cassava4.1_000956m<br>cassava4.1_000826m<br>cassava4.1_028612m	MeAGO10<br>MeAGO7b<br>MeAGO5<br>MeAGO4<br>MeAGOlike<br>MeAGO7a<br>MeAGO1	Phytozome	√
Malus domestica	MDP0000161046<br>MDP0000118779<br>MDP0000215105<br>MDP0000071268<br>MDP0000159246<br>MDP0000069525<br>MDP0000209079<br>MDP0000774227<br>MDP0000232035<br>MDP0000199819<br>MDP0000191579	MdAGO10c<br>MdAGO7a<br>MdAGO9<br>MdAGO10a<br>MdAGO7b<br>MdAGO1a<br>MdAGO4<br>MdAGO5b<br>MdAGO5a<br>MdAGO1b<br>MdAGO10b	Phytozome	√
Medicago truncatula	AES62761.1<br>AES64710.1<br>AES68604.1<br>AES71838.1<br>AES90053.1<br>AES91411.1<br>AES96874.1<br>AET00054.1<br>AET00055.1<br>AET00057.1	MtAGO4a<br>MtAGO2<br>MtAGO4e<br>MtAGO6<br>MtAGO3<br>MtAGO10<br>MtAGO7<br>MtAGO4b<br>MtAGO4c<br>MtAGO4d	NCBI	
Mimulus guttatus	mgv1a001065m<br>mgv1a000944m<br>mgv1a000822m<br>mgv1a000922m<br>mgv1a001365m<br>mgv1a000545m<br>mgv1a001084m	MgAGO6<br>MgAGO4<br>MgAGO5<br>MgAGO10<br>MgAGO7<br>MgAGO1<br>MgAGO9	Phytozome	√
Nicotiana attenuata	GBGF01000001.1<br>GBGF01000002.1<br>GBGF01000003.1<br>GBGF01000004.1<br>GBGF01000005.1<br>GBGF01000006.1<br>GBGF01000007.1<br>GBGF01000008.1	NaAGO1a<br>NaAGO1b<br>NaAGO1c<br>NaAGO2<br>NaAGO4a<br>NaAGO4b<br>NaAGO5<br>NaAGO7	GenBank	√

	GBGF01000009.1<br>GBGF01000010.1<br>GBGF01000011.1	NaAGO8<br>NaAGO9<br>NaAGO10		
Nicotiana benthamiana	ABC61503.1<br>ABC61504.1<br>ABC61505.1<br>BAJ09698.1	NbAGO4-2<br>NbAGO4-1<br>NbAGO1-2<br>NbAGO1-1	NCBI	
Nicotiana tabacum	ABC61502.1	NtAGO1	NCBI	
Oryza sativa	LOC_Os02g45070.1<br>LOC_Os04g47870.1<br>LOC_Os02g58490.1<br>LOC_Os06g51310.2<br>LOC_Os04g52540.1<br>LOC_Os04g52550.1<br>LOC_Os01g16870.3<br>LOC_Os04g06770.2<br>LOC_Os07g09020.1<br>LOC_Os03g58600.1<br>LOC_Os03g57560.1<br>LOC_Os03g33650.1<br>LOC_Os06g39640.1<br>LOC_Os02g07310.1<br>LOC_Os03g47820.1<br>LOC_Os03g47830.1<br>LOC_Os07g28850.1	OsAGO1A<br>OsAGO1B<br>OsAGO1C<br>OsAGO1D<br>OsAGO2<br>OsAGO3<br>OsAGO4A<br>OsAGO4B<br>OsAGO14<br>OsMLE1<br>OsAGO13<br>OsSHL4<br>OsPNH1<br>OsAGO17<br>OsAGO12<br>OsAGO11<br>OsAGO18	TIGR	
Pelargonium hortorum	ACZ04920.1	PhAGO4like	NCBI	
Physcomitrella patens	EDQ50204.1<br>EDQ61013.1<br>EDQ66080.1<br>EDQ77668.1<br>EDQ79477.1<br>EDQ82032.1	PptAGOlike1<br>PptAGO5<br>PptAGOlike2<br>PptAGO1<br>PptAGOlike3<br>PptAGO10	NCBI	
Phaseolus vulgaris	Phvulv091017154m<br>Phvulv091002019m<br>Phvulv091006909m<br>Phvulv091010302m<br>Phvulv091023278m<br>Phvulv091011331m	PvAGO10b<br>PvAGO7<br>PvAGO4<br>PvAGO9<br>PvAGO10a<br>PvAGO1	Phytozome	√
Picea glauca	AAY67884.1	PgAGO	NCBI	
Pisum sativum	ABL63483.1<br>ABL63484.1	PsAGO2<br>PsAGO1	NCBI	
Populus trichocarpa	EEE72070.1<br>EEE82967.1<br>EEE88272.1<br>EEE88323.1<br>EEE89922.1<br>EEE92366.1<br>EEE96558.1<br>EEE98804.1<br>EEF02279.1	PtAGOlike1<br>PtAGOlike2<br>PtAGOlike3<br>PtAGO4a<br>PtAGO10<br>PtAGO4b<br>PtAGO1<br>PtAGO6<br>PtAGO7	NCBI	
Prunus persica	ppa000990m<br>ppa024131m<br>ppa000759m<br>ppa000547m	PprAGO4a<br>PprAGO5<br>PprAGO7<br>PprAGO1b	Phytozome	√

	ppa000619m<br>ppa017623m<br>ppa000823m	PprAGO1a<br>PprAGO4b<br>PprAGO10		
Ricinus communis	27389.m000069<br>29589.m001295<br>29677.m000188<br>29684.m000322<br>29807.m000479<br>29813.m001544<br>29828.m000386<br>29844.m003212	RcAGO6<br>RcAGO5<br>RcAGO1<br>RcAGO4a<br>RcAGO10b<br>RcAGO7<br>RcAGO4b<br>RcAGO10a	Phytozome	√
Selaginella moellendorfii	EFJ14095.1<br>EFJ14096.1<br>EFJ24991.1	SmAGO10<br>SmAGO1a<br>SmAGO1b	NCBI	
Solanum lycopersicum	SlSBM_S02331_01.70<br>SlSBM_S01043_02.10<br>SlSBM_S01043_02.20<br>SlSBM_S01043_02.30<br>SlSBM_S00897_03.10	SlAGO4b<br>SlAGOlike1<br>SlAGOlike2<br>SlAGOlike3<br>SlAGO4a	Tomato SBM database	√
Sorghum bicolor	EER88597.1<br>EER89008.1<br>EER92100.1<br>EES07823.1<br>EES12675.1<br>EES18816.1<br>EES18841.1	SbAGO10a<br>SbAGO1b<br>SbAGO7<br>SbAGO1a<br>SbAGO10b<br>SbAGO4<br>SbAGO10c	NCBI	
Thellungiella halophila	Thhalv10016181m<br>Thhalv10016224m<br>Thhalv10011196m<br>Thhalv10006643m<br>Thhalv10018068m<br>Thhalv10001902m<br>Thhalv10015501m<br>Thhalv10012624m<br>Thhalv10003139m	ThAGO5<br>ThAGO6<br>ThAGO1<br>ThAGO2<br>ThAGO7<br>ThAGO4<br>ThAGO8<br>ThAGO9<br>ThAGO10	Phytozome	√
Vitis vinifera	XP_002279408.1<br>XP_002275928.1<br>XP_002281687.1<br>XP_002267746.1<br>XP_002264978.1<br>XP_002271225.1<br>XP_002271699.2<br>XP_002264527.2<br>XP_002274220.2<br>XP_002271447.2<br>XP_003634638.1	VvAGO10like<br>VvAGO4<br>VvPNH1<br>VvAGO7like<br>VvAGO4Alike<br>VvAGO1like<br>VvAGO5like<br>VvMEL1like<br>VvAGO2like<br>VvAGO16like<br>VvAGO1Blike	NCBI	
Volvox carteri	EFJ46144.1	VcAGOlike	NCBI	
Zea mays	GRMZM2G441583_T01<br>GRMZM2G079080_T02<br>AC189879.3_FGT003<br>GRMZM2G039455_T01<br>GRMZM2G589579_T01<br>AC209206.3_FGT011<br>GRMZM2G361518_T01	ZmAGO1b<br>ZmAGO10a<br>ZmAGO10b<br>ZmAGO1a<br>ZmAGO4<br>ZmAGO1c<br>ZmAGO1d	Phytozome	√

Kluyveromyces polysporus	4F1N_A	YeastAGO	PDB	
Tribolium castaneum	XP_971295.2<br>EFA02921.1<br>EFA04626.1<br>EFA09197.1<br>EFA11590.1	TcAGO1b<br>TcAGO3<br>TcAGO2b<br>TcAGO1a<br>TcAGO2a	NCBI	
Homo sapiens	4F3T_A	HsAGO2	PDB	
Aedes aegypti	ACR56327.1	AaAGO2	NCBI	
Amphimedon queenslandica	XP_003385988.1<br>XP_003382578.1	AqAGO2like1<br>AqAGO2like2	NCBI<br>NCBI	
Anopheles darlingi	ETN67307.1	AdAGO2	NCBI	
Bombyx mori	BAF73719.1<br>BAD91160.2	BmoAGO1<br>BmoAGO2	NCBI	
Bos taurus	AAS21301.1	BtAGO2	NCBI	
Brugia malayi	XP_001894256.1	BmaAGO2	NCBI	
Crassostrea gigas	EKC19600.1	CgAGO2	NCBI	
Danaus plexippus	EHJ71131.1	DpAGO1	NCBI	
Danio rerio	AFU66008.1	DrAGO2	NCBI	
Drosophila immigrans	AHZ92157.1	DiAGO2	NCBI	
Drosophila melanogaster	AAF58313.1<br>AAF49619.2	DmAGO1<br>DmAGO2	NCBI<br>NCBI	
Drosophila santomea	ABB54736.1	DsAGO2	NCBI	
Ephydatia fluviatilis	BAJ07611.1	EfAGO	NCBI	
Hydra vulgaris	XP_002160880.2	HyvAGO2like	NCBI	
Isodiametrica pulchra	CAQ03960.1	IpPIWIlike2	NCBI	
Marsupenaeus japonicus	BAM37459.1	MjAGO2	NCBI	
Musca domestica	XP_005175308.1	MudAGO1like	NCBI	
Nilaparvata lugens	AGH30326.1<br>AGE12619.1	NlAGO1<br>NlAGO2	NCBI	
Nematostella vectensis	AGW15594.1<br>AGW15595.1	NvAGO1<br>NvAGO2	NCBI	
Oikopleura dioica	CAP07637.1	OdAGO2	NCBI	
Sarcophilus harrisii	XP_003760467.1	ShAGO2	NCBI	
Spodoptera litura	AHC98009.1<br>AHC98010.1	SplAGO1<br>SplAGO2	NCBI	
Strongylocentrotus purpuratus	ACE63524.1	SpAGO1	NCBI	
Sus scrofa	ADH59736.1	SsAGO2	NCBI	
Trichuris trichiura	CDW54952.1	TtAGO2	NCBI	
Xenopus laevis	ACA52290.1	XlAGO2	NCBI	
