# Supplementary figures and images for: Molecular evolution and diversification of the Argonaute family of proteins in plants
Source: BMC Plant Biol. 2015 Jan 28;15:23. doi: 10.1186/s12870-014-0364-6 (PMC4318128; doi:10.1186/s12870-014-0364-6)

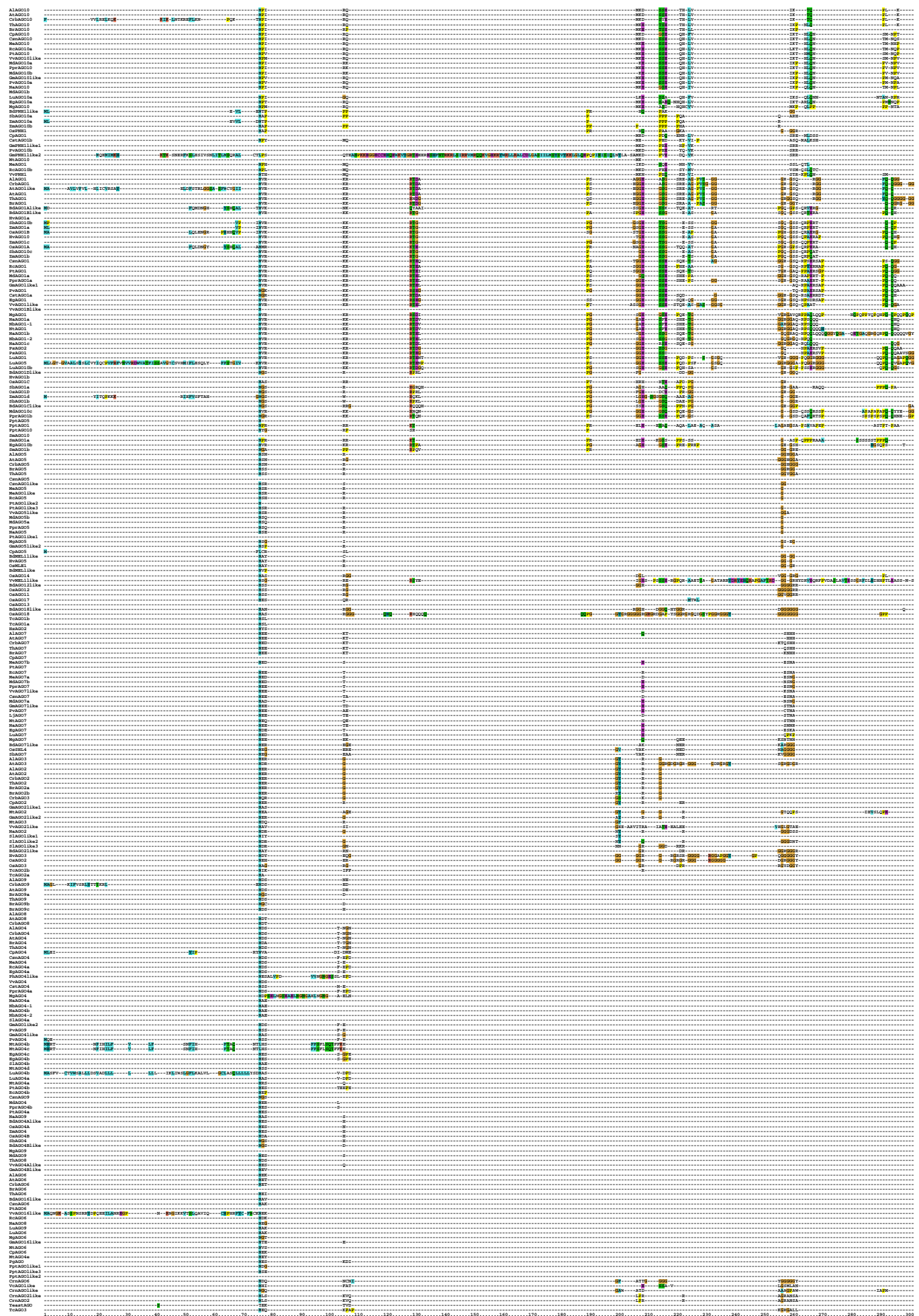

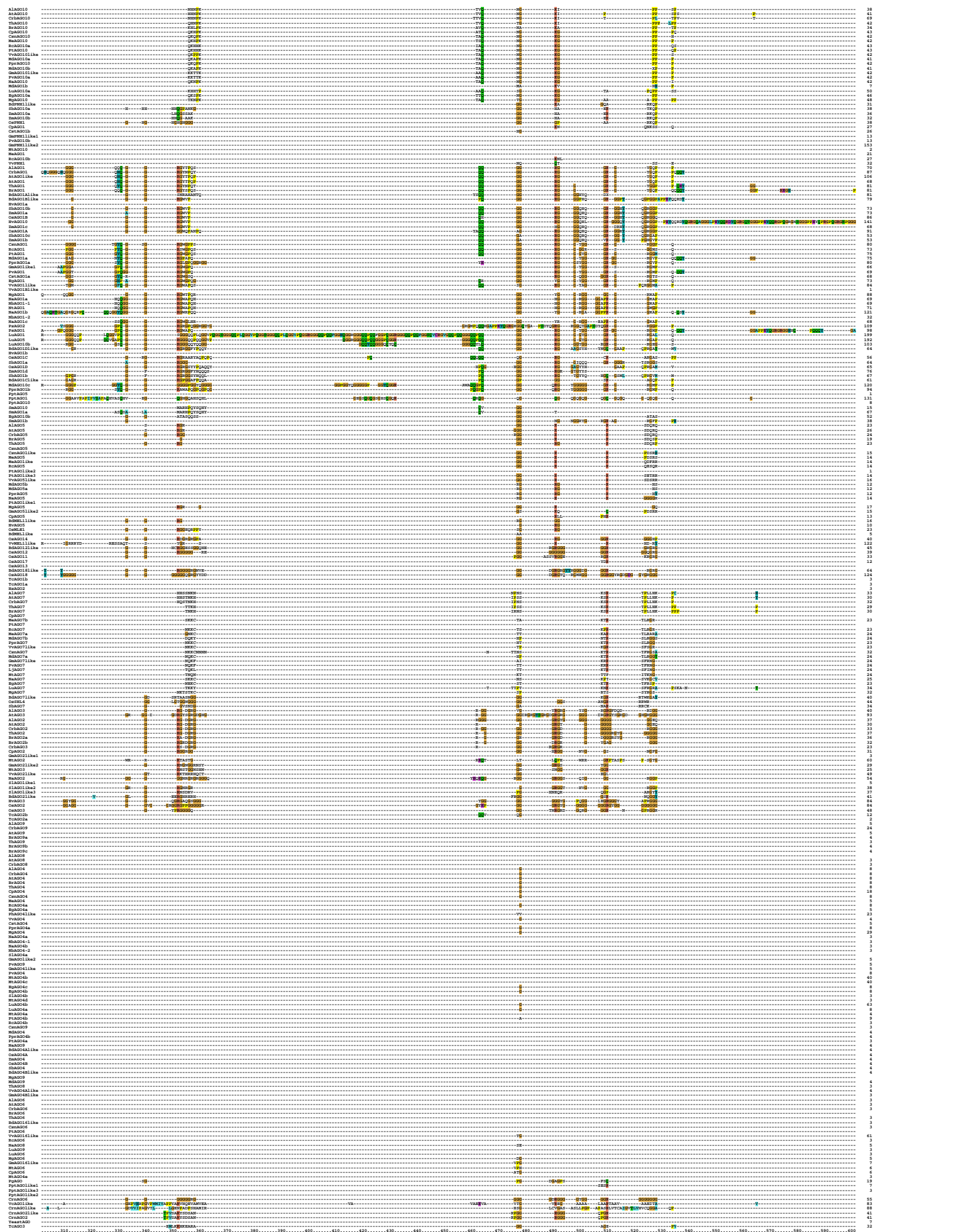

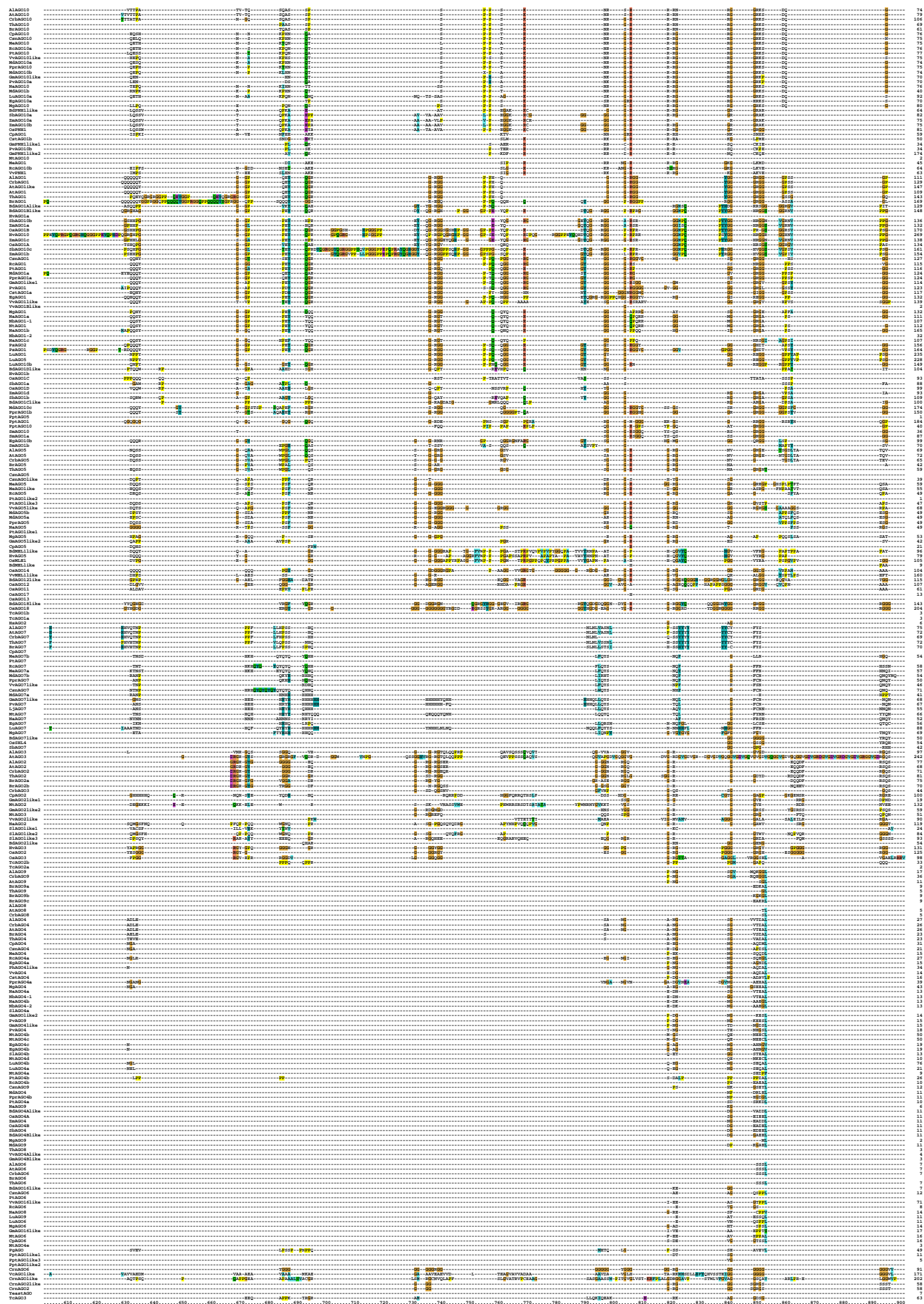

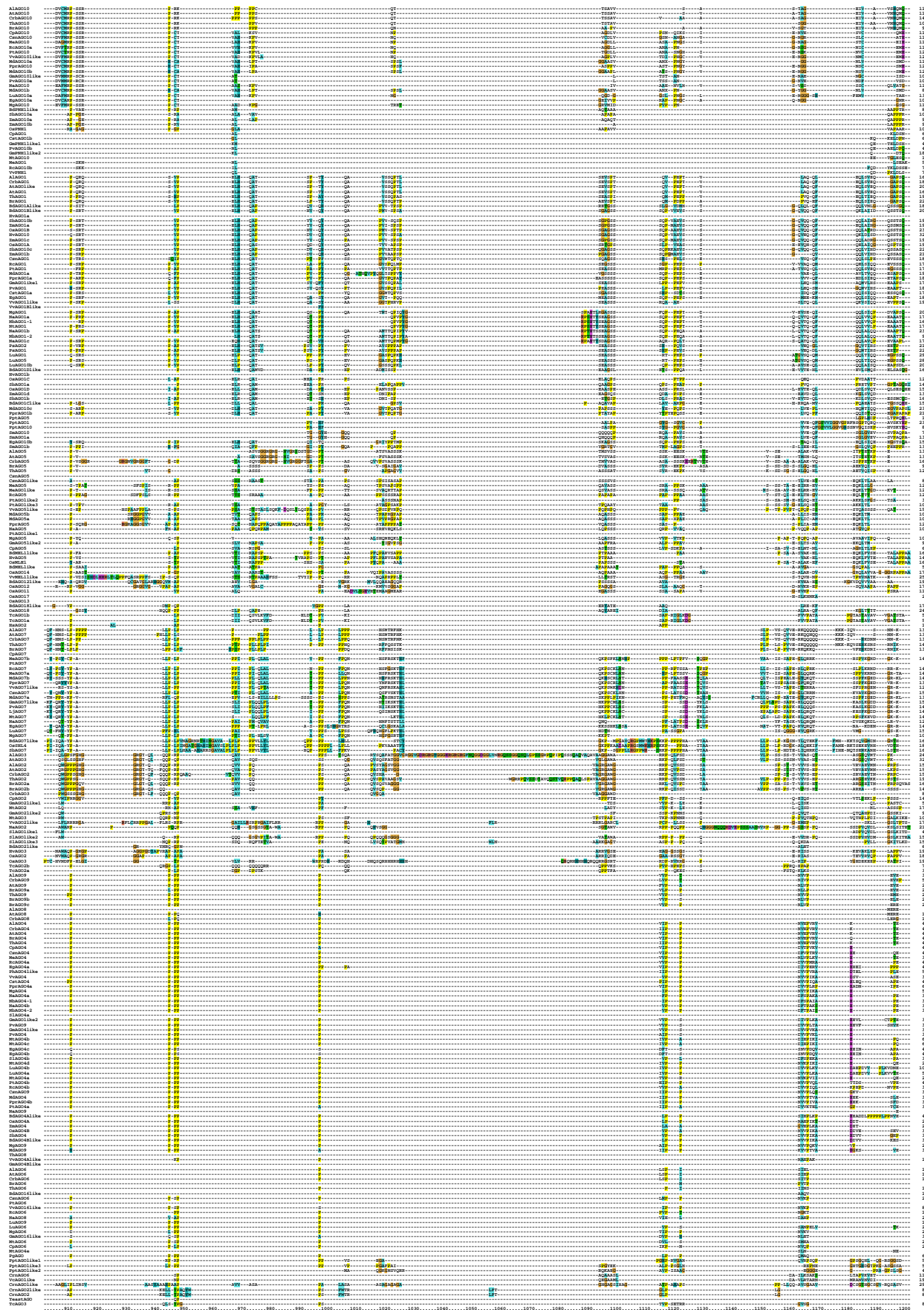

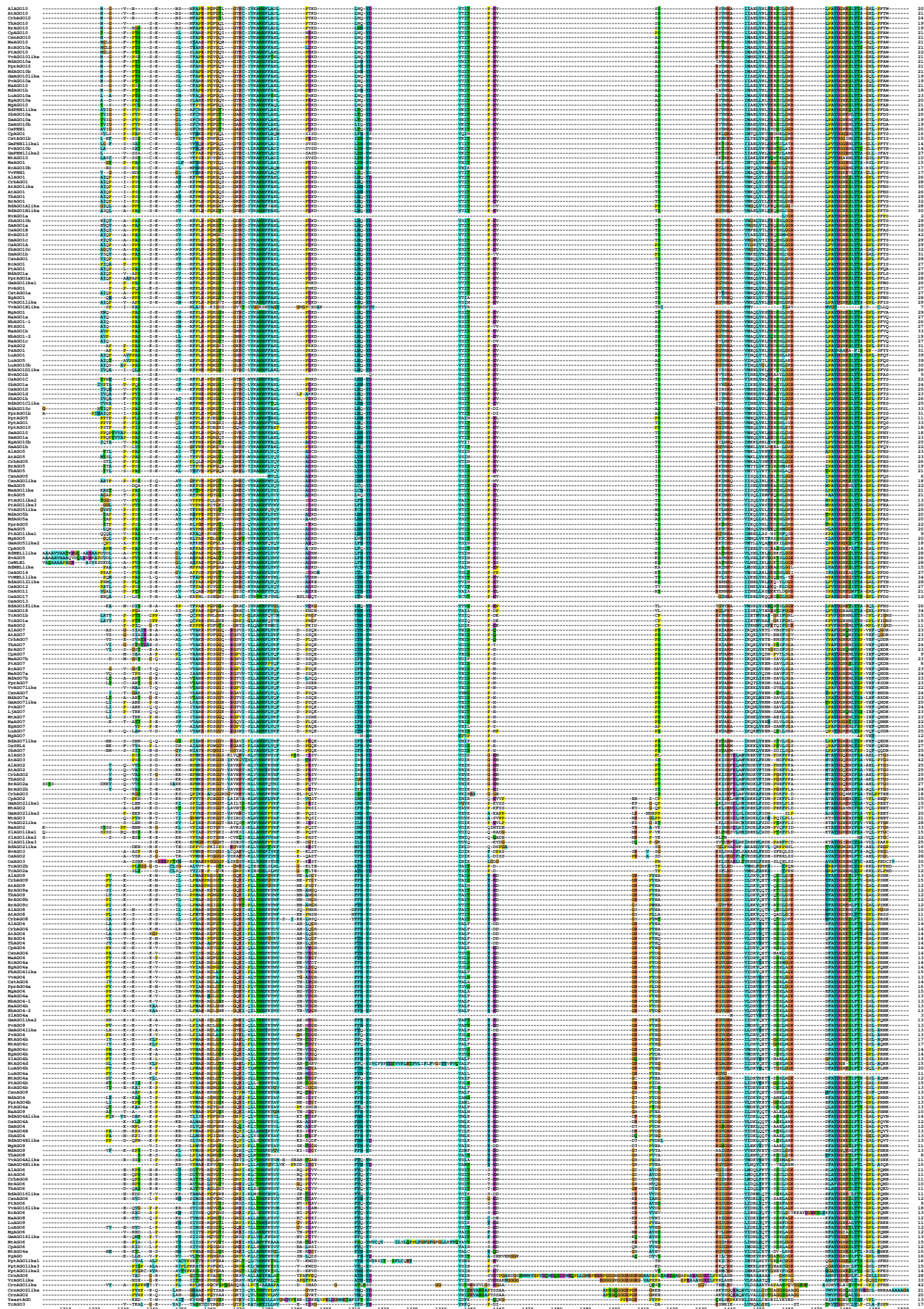

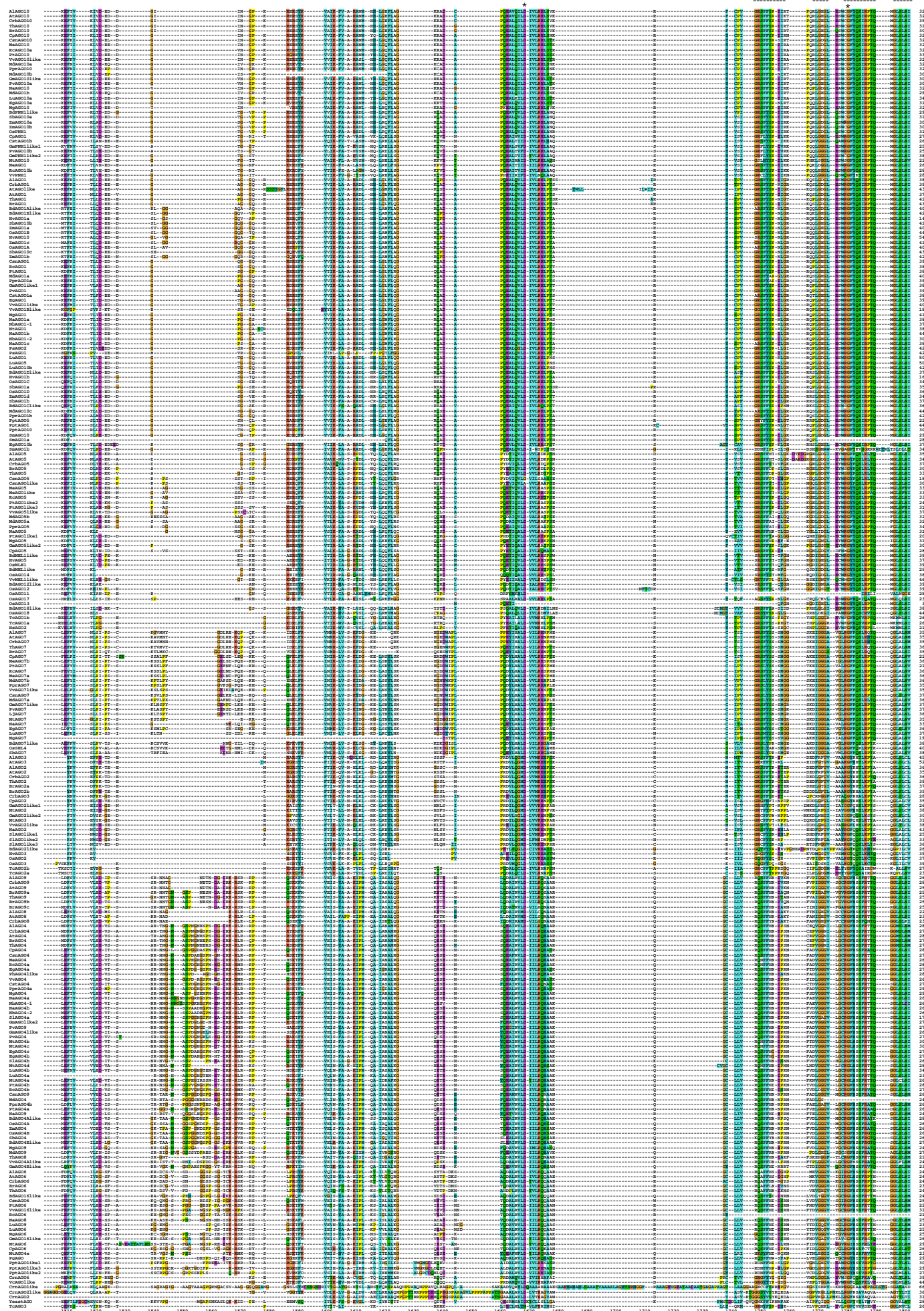

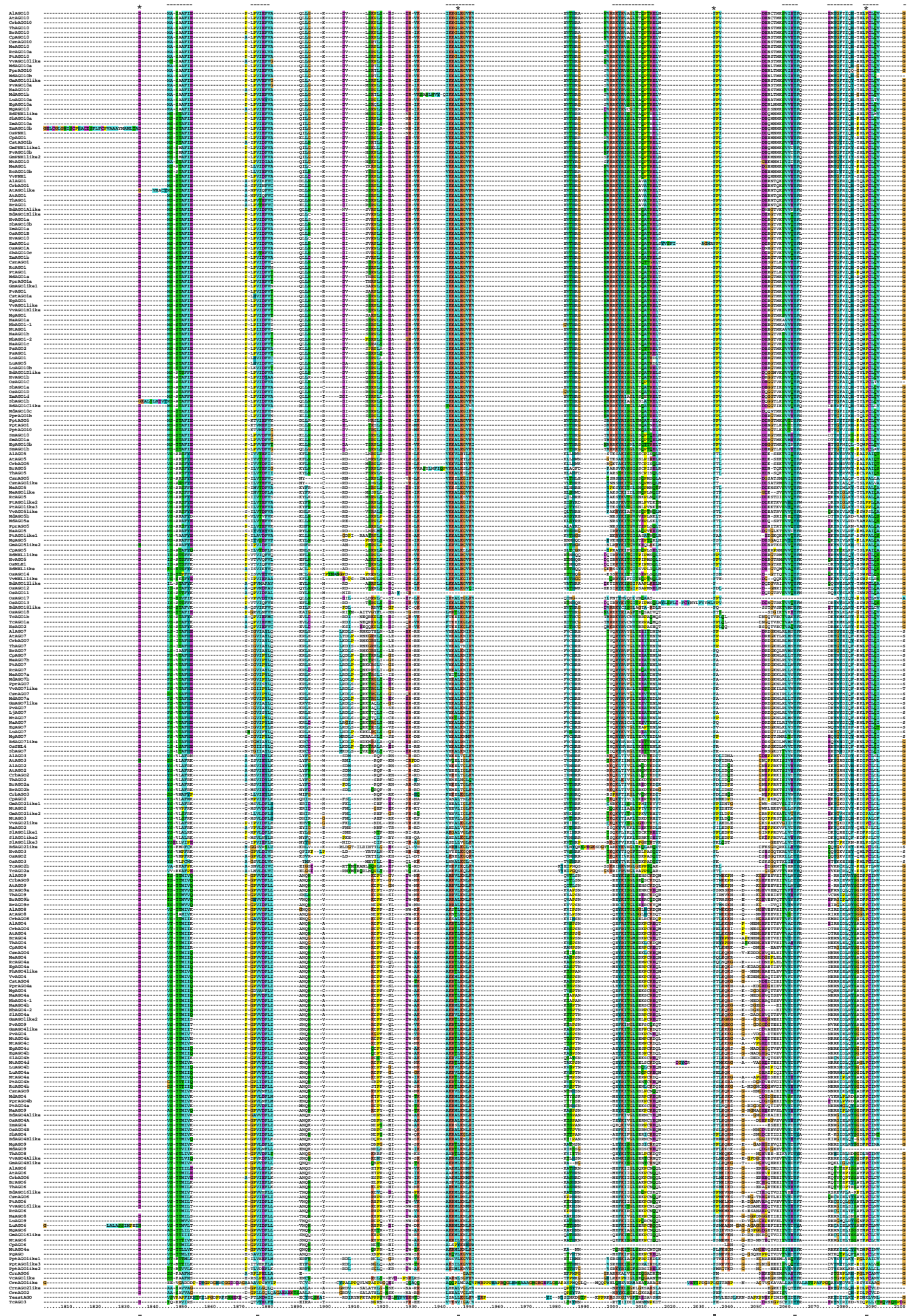

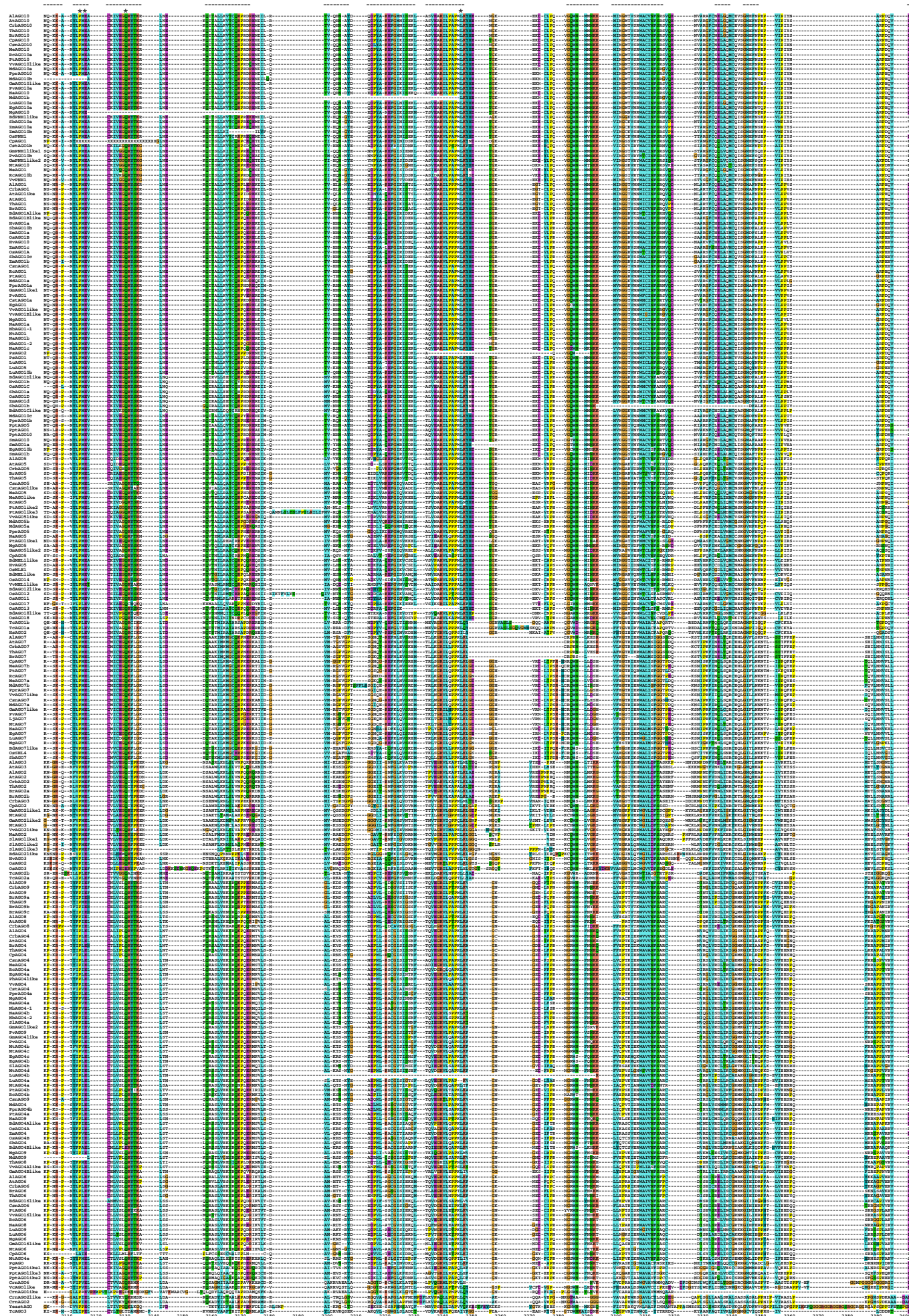

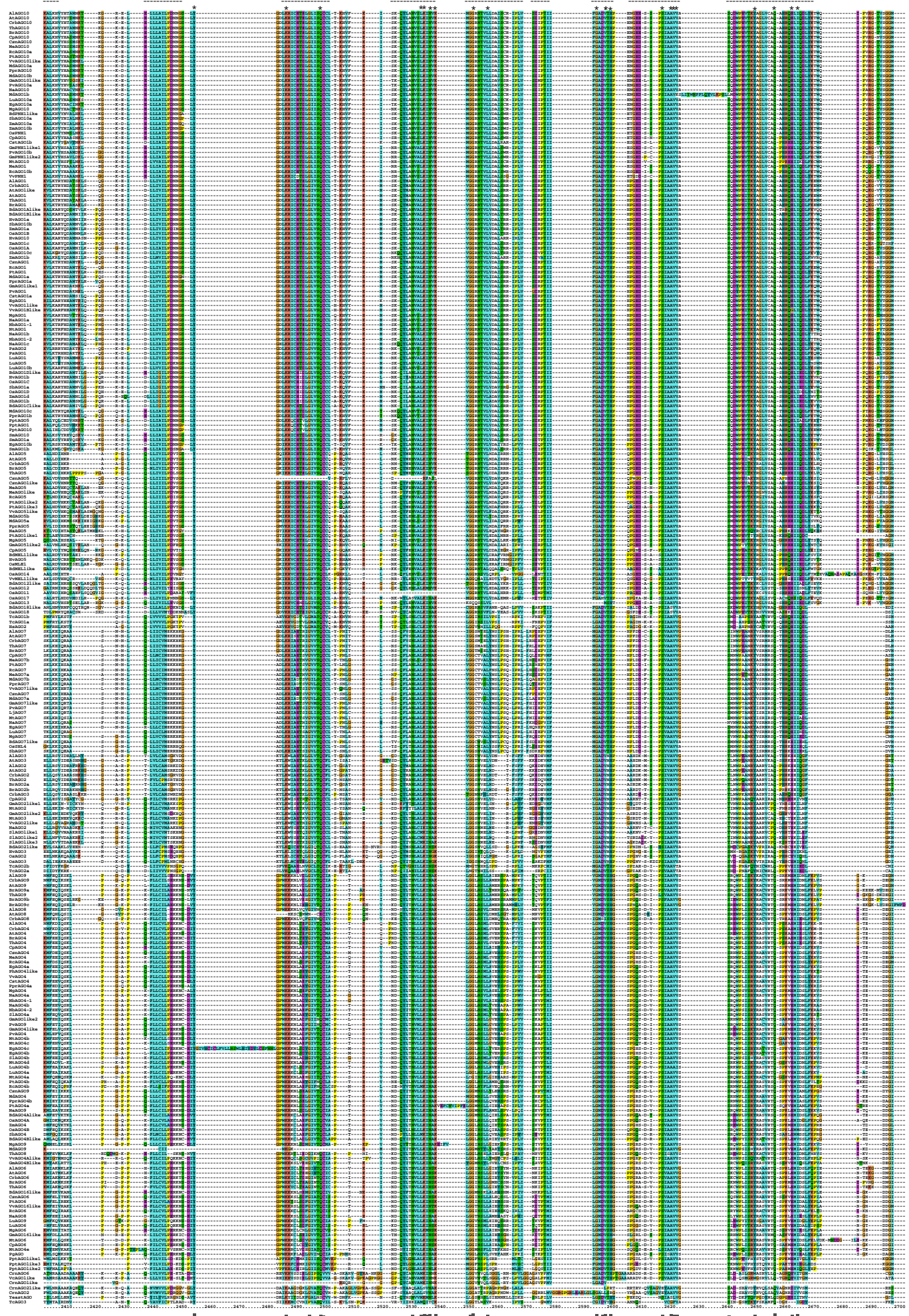

66  
67  
68  
69  
70  
71  
72  
73  
74  
75  
76  
77  
78  
79  
80  
81  
82  
83  
84  
85  
86  
87  
88  
89  
90  
91  
92  
93  
94  
95  
96  
97  
98  
99  
100  
101  
102  
103  
104  
105  
106  
107  
108  
109  
110  
111  
112  
113  
114  
115  
116  
117  
118  
119  
120  
121  
122  
123  
124  
125  
126  
127  
128  
129  
130  
131  
132  
133  
134  
135  
136  
137  
138  
139  
140  
141  
142  
143  
144  
145  
146  
147  
148  
149  
150  
151  
152  
153  
154  
155  
156  
157  
158  
159  
160  
161  
162  
163  
164  
165  
166  
167  
168  
169  
170  
171  
172  
173  
174  
175  
176  
177  
178  
179  
180  
181  
182  
183  
184  
185  
186  
187  
188  
189  
190  
191  
192  
193  
194  
195  
196  
197  
198  
199  
200  
201  
202  
203  
204  
205  
206  
207  
208  
209  
210  
211  
212  
213  
214  
215  
216  
217  
218  
219  
220  
221  
222  
223  
224  
225  
226  
227  
228  
229  
230  
231  
232  
233  
234  
235  
236  
237  
238  
239  
240  
241  
242  
243  
244  
245  
246  
247  
248  
249  
250  
251  
252  
253  
254  
255  
256  
257  
258  
259  
260  
261  
262  
263  
264  
265  
266  
267  
268  
269  
270  
271  
272  
273  
274  
275  
276  
277  
278  
279  
280  
281  
282  
283  
284  
285  
286  
287  
288  
289  
290  
291  
292  
293  
294  
295  
296  
297  
298  
299  
300  
301  
302  
303  
304  
305  
306  
307  
308  
309  
310  
311  
312  
313  
314  
315  
316  
317  
318  
319  
320  
321  
322  
323  
324  
325  
326  
327  
328  
329  
330  
331  
332  
333  
334  
335  
336  
337  
338  
339  
340  
341  
342  
343  
344  
345  
346  
347  
348  
349  
350  
351  
352  
353  
354  
355  
356  
357  
358  
359  
360  
361  
362  
363  
364  
365  
366  
367  
368  
369  
370  
371  
372  
373  
374  
375  
376  
377  
378  
379  
380  
381  
382  
383  
384  
385  
386  
387  
388  
389  
390  
391  
392  
393  
394  
395  
396  
397  
398  
399  
400  
401  
402  
403  
404  
405  
406  
407  
408  
409  
410  
411  
412  
413  
414  
415  
416  
417  
418  
419  
420  
421  
422  
423  
424  
425  
426  
427  
428  
429  
430  
431  
432  
433  
434  
435  
436  
437  
438  
439  
440  
441  
442  
443  
444  
445  
446  
447  
448  
449  
450  
451  
452  
453  
454  
455  
456  
457  
458  
459  
460  
461  
462  
463  
464  
465  
466  
467  
468  
469  
470  
471  
472  
473  
474  
475  
476  
477  
478  
479  
480  
481  
482  
483  
484  
485  
486  
487  
488  
489  
490  
491  
492  
493  
494  
495  
496  
497  
498  
499  
500  
501  
502  
503  
504  
505  
506  
507  
508  
509  
510  
511  
512  
513  
514  
515  
516  
517  
518  
519  
520  
521  
522  
523  
524  
525  
526  
527  
528  
529  
530  
531  
532  
533  
534  
535  
536  
537  
538  
539  
540  
541  
542  
543  
544  
545  
546  
547  
548  
549  
550  
551  
552  
553  
554  
555  
556  
557  
558  
559  
560  
561  
562  
563  
564  
565  
566  
567  
568  
569  
570  
571  
572  
573  
574  
575  
576  
577  
578  
579  
580  
581  
582  
583  
584  
585  
586  
587  
588  
589  
590  
591  
592  
593  
594  
595  
596  
597  
598  
599  
600  
601  
602  
603  
604  
605  
606  
607  
608  
609  
610  
611  
612  
613  
614  
615  
616  
617  
618  
619  
620  
621  
622  
623  
624  
625  
626  
627  
628  
629  
630  
631  
632  
633  
634  
635  
636  
637  
638  
639  
640  
641  
642  
643  
644  
645  
646  
647  
648  
649  
650  
651  
652  
653  
654  
655  
656  
657  
658  
659  
660  
661  
662  
663  
664  
665  
666  
667  
668  
669  
670  
671  
672  
673  
674  
675  
676  
677  
678  
679  
680  
681  
682  
683  
684  
685  
686  
687  
688  
689  
690  
691  
692  
693  
694  
695  
696  
697  
698  
699  
700  
701  
702  
703  
704  
705  
706  
707  
708  
709  
710  
711  
712  
713  
714  
715  
716  
717  
718  
719  
720  
721  
722  
723  
724  
725  
726  
727  
728  
729  
730  
731  
732  
733  
734  
735  
736  
737  
738  
739  
740  
741  
742  
743  
744  
745  
746  
747  
748  
749  
750  
751  
752  
753  
754  
755  
756  
757  
758  
759  
760  
761  
762  
763  
764  
765  
766  
767  
768  
769  
770  
771  
772  
773  
774  
775  
776  
777  
778  
779  
780  
781  
782  
783  
784  
785  
786  
787  
788  
789  
790  
791  
792  
793  
794  
795  
796  
797  
798  
799  
800  
801  
802  
803  
804  
805  
806  
807  
808  
809  
810  
811  
812  
813  
814  
815  
816  
817  
818  
819  
820  
821  
822  
823  
824  
825  
826  
827  
828  
829  
830  
831  
832  
833  
834  
835  
836  
837  
838  
839  
840  
841  
842  
843  
844  
845  
846  
847  
848  
849  
850  
851  
852  
853  
854  
855  
856  
857  
858  
859  
860  
861  
862  
863  
864  
865  
866  
867  
868  
869  
870  
871  
872  
873  
874  
875  
876  
877  
878  
879  
880  
881  
882  
883  
884  
885  
886  
887  
888  
889  
890  
891  
892  
893  
894  
895  
896  
897  
898  
899  
900  
901  
902  
903  
904  
905  
906  
907  
908  
909  
910  
911  
912  
913  
914  
915  
916  
917  
918  
919  
920  
921  
922  
923  
924  
925  
926  
927  
928  
929  
930  
931  
932  
933  
934  
935  
936  
937  
938  
939  
940  
941  
942  
943  
944  
945  
946  
947  
948  
949  
950  
951  
952  
953  
954  
955  
956  
957  
958  
959  
960  
961  
962  
963  
964  
965  
966  
967  
968  
969  
970  
971  
972  
973  
974  
975  
976  
977  
978  
979  
980  
981  
982  
983  
984  
985  
986  
987  
988  
989  
990  
991  
992  
993  
994  
995  
996  
997  
998  
999  
1000

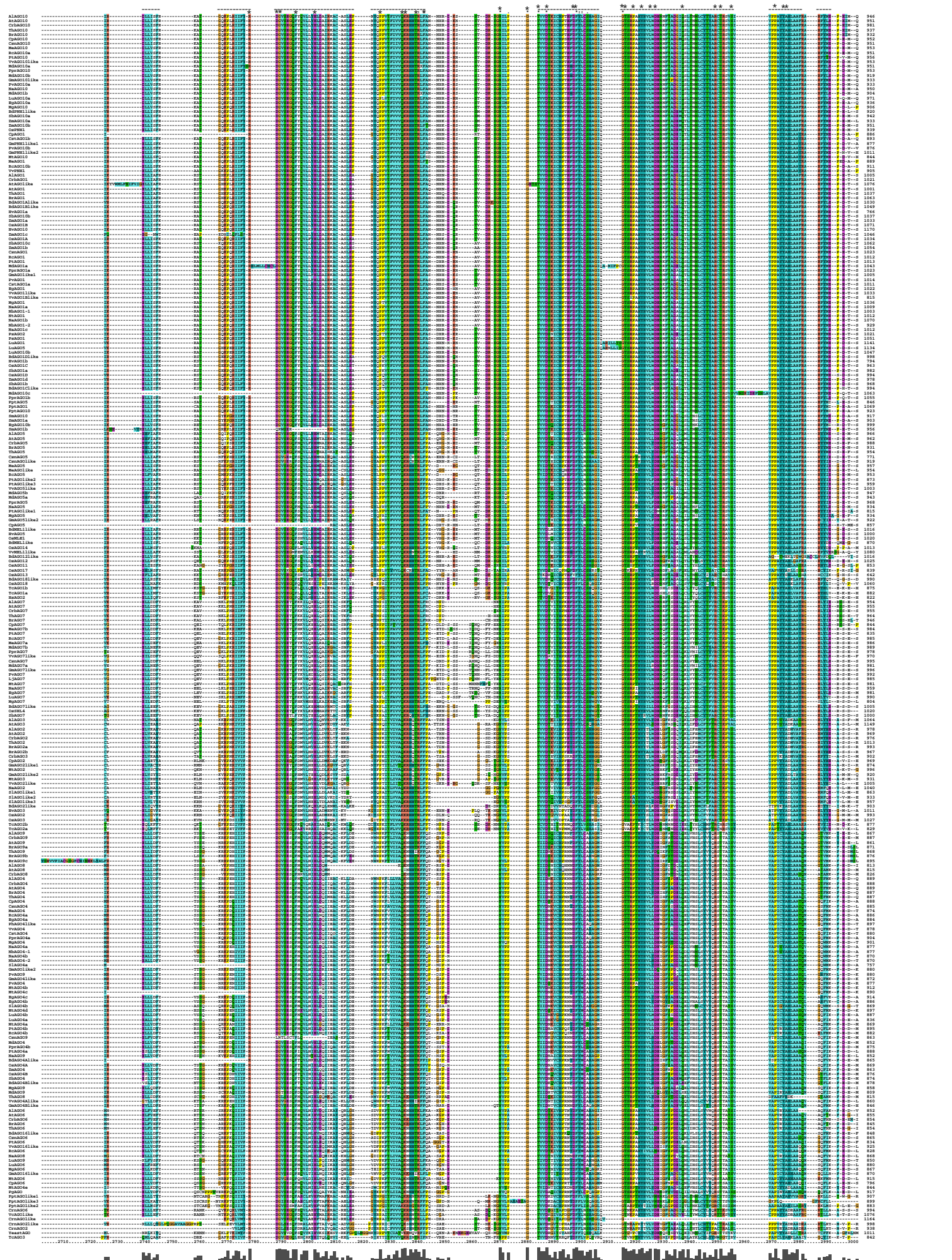

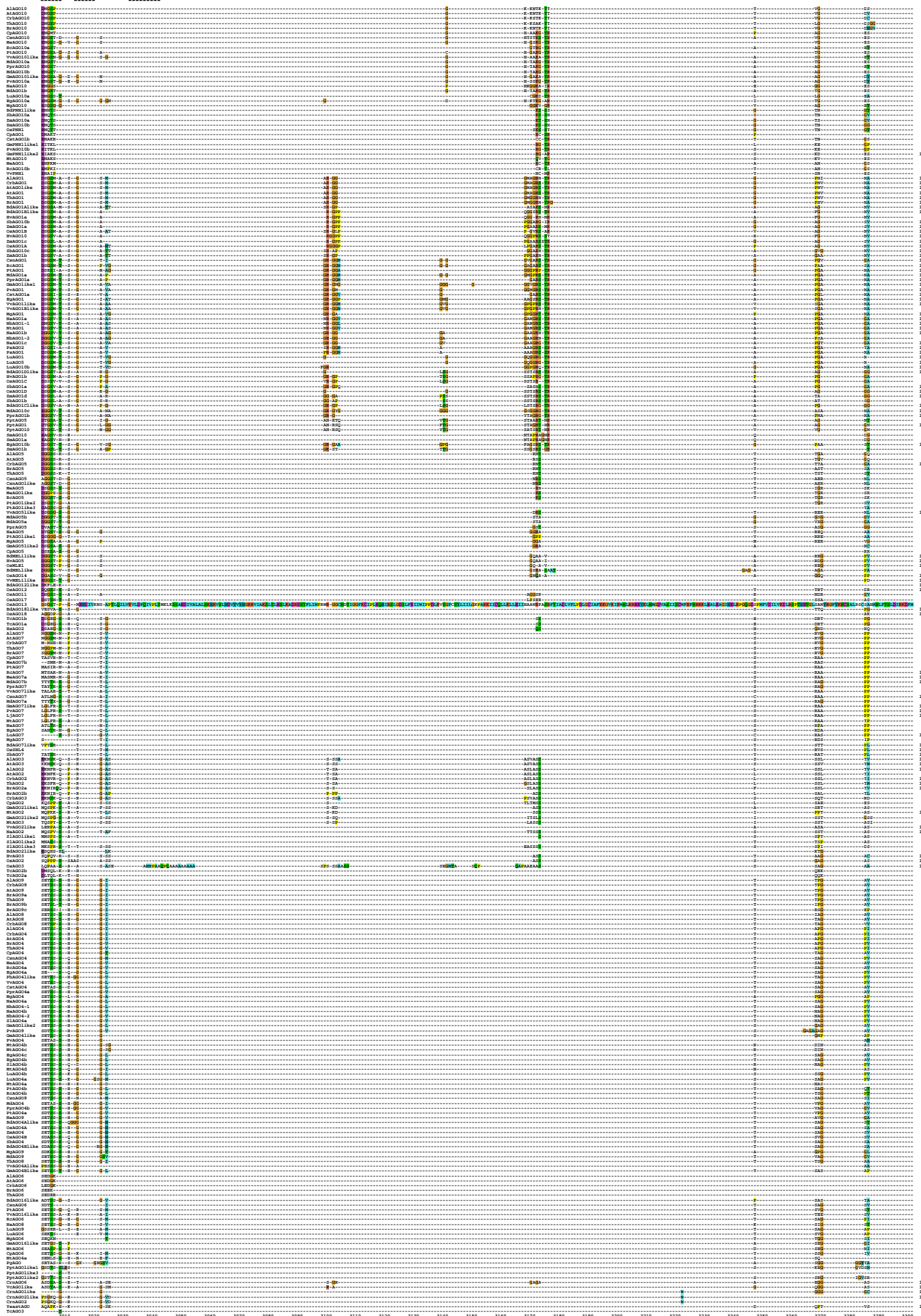

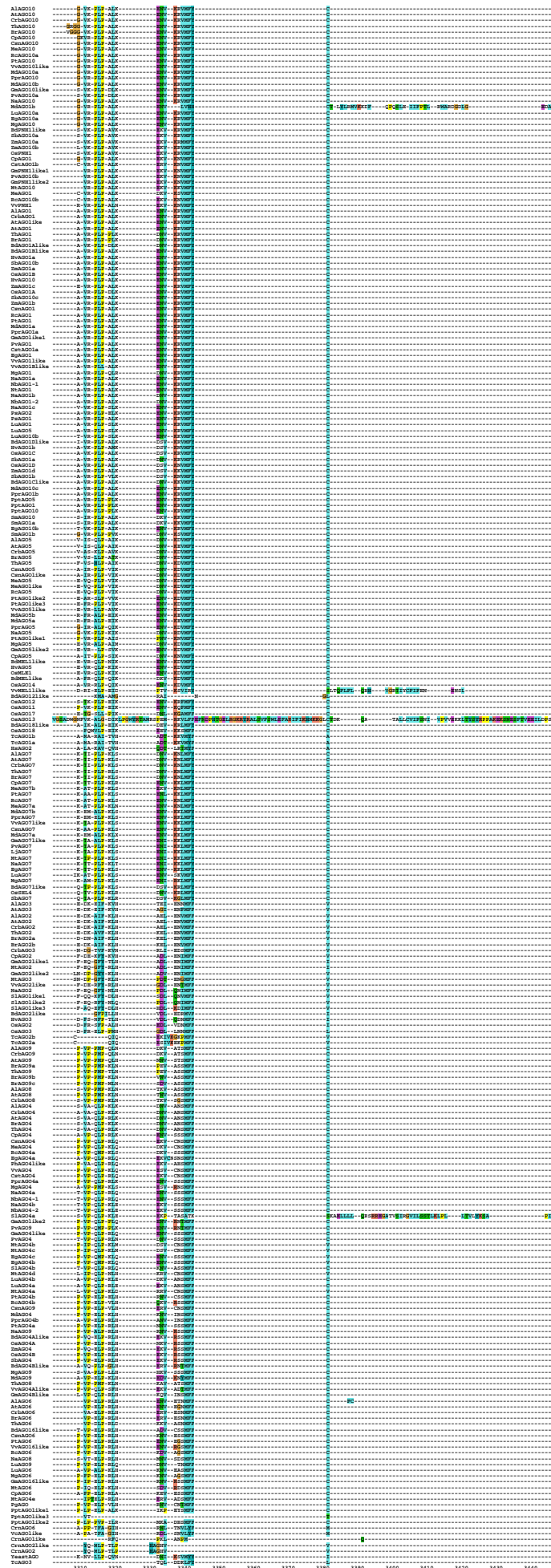

Supplement: Additional file 2: Figure S1. — Multiple sequence alignment of ‘AGO dataset I’ by using the MAFFT v 7.130b with option ‘L-INS-I’. A total of 270 AGO sequences were aligned. In total, 55 columns are having column score >90 (score for conservation of physio-chemical properties of residues for a particular column, calculated by ClustalX; range: 0 - 100) are marked with star. The columns marked on the top of the MSA are the positions that were retained in the ‘plant AGO dataset II’ after trimming (the co-ordinates of positions are: 1256-1262, 1267-1282, 1444-1460, 1474-1490, 1598-1611, 1659-1677, 1747-1757, 1767-1772, 1778-1788, 1795-1800, 1844-1852, 1873-1879, 1941-1950, 1999-2013, 2058-2063, 2074-2082, 2086-2091, 2100-2107, 2111-2116, 2123-2134, 2157-2172, 2198-2206, 2213-2229, 2233-2246, 2282-2292, 2298-2315, 2333-2337, 2343-2347, 2400-2406, 2436-2449, 2484-2500, 2522-2537, 2548-2567, 2571-2577, 2592-2599, 2605-2622, 2639-2668, 2738-2743, 2766-2777, 2787-2815, 2822-2841, 2883-2905, 2914-2955, 2968-2980, 2986-2990, 3001-3007, 3312-3319 and 3331-3341. A total of 620 positions were retained). [file 12870_2014_364_MOESM2_ESM.pdf]

A

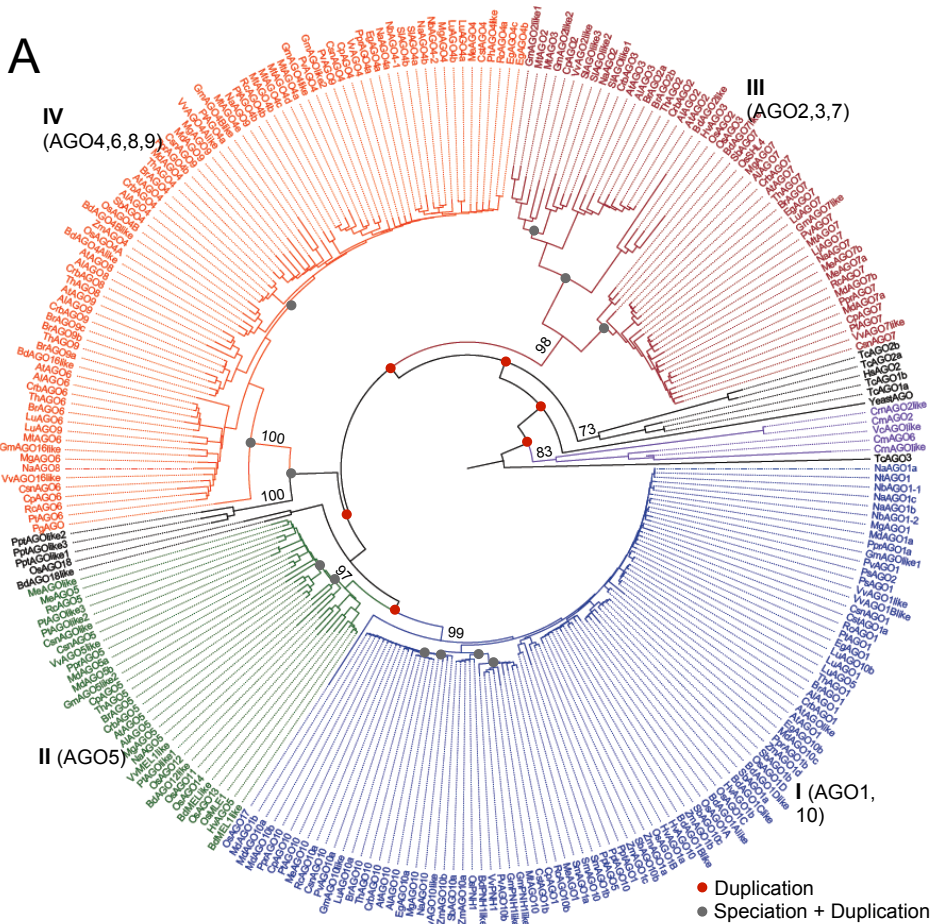

B

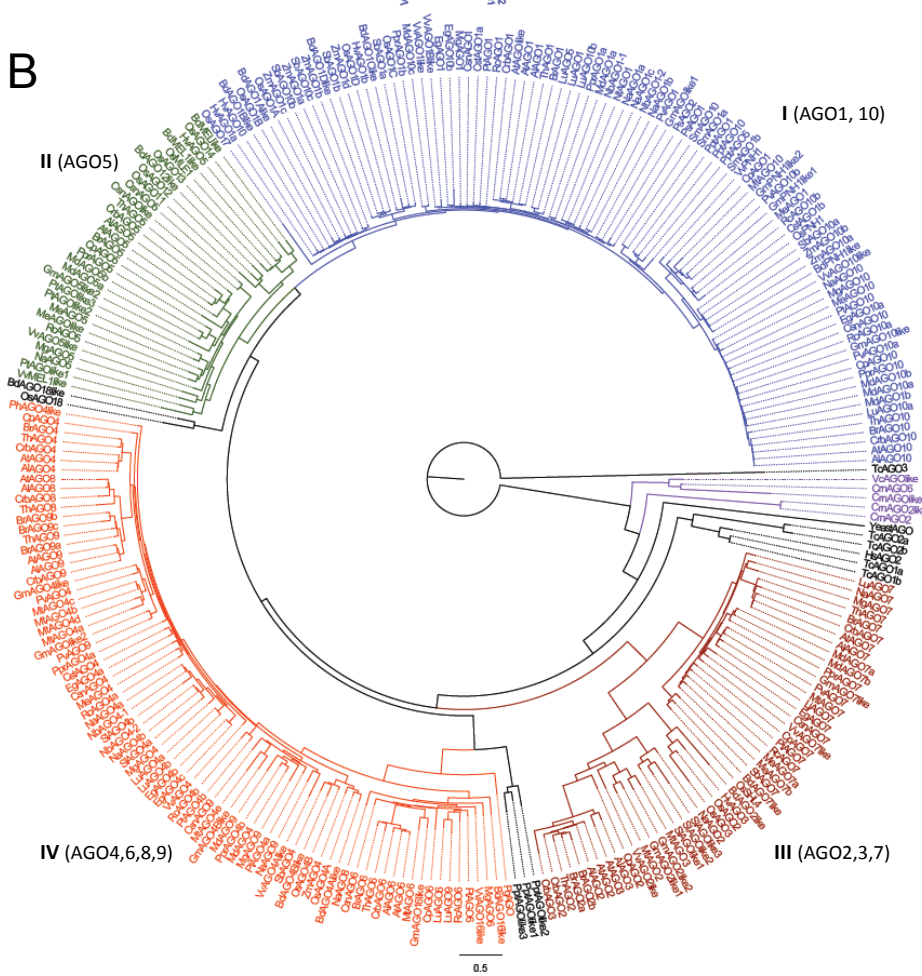

Supplement: Additional file 3: Figure S2. — Phylogenetic classification of plant AGOs. (A) Neighbor-Joining (NJ) phylogenetic tree of the plant AGOs (plant ‘AGO dataset II’). The brown colored nodes indicate duplication whereas the grey colored circles indicate the speciation followed by duplication nodes respectively. Clade robustness was assessed with 100 bootstrap replicates. (B) Maximum Likelihood (ML) phylogeny of all the AGOs in current study (‘plant AGO dataset II’). RAxML v 7.2.8 was used to run the ML analyses. The phylogeny was reconstructed by using the Jones-Taylor-Thornton (JTT) amino acid substitution model with 'G + I' parameter. Nodes show the AGOs from the respective plant. [file 12870_2014_364_MOESM3_ESM.pdf]

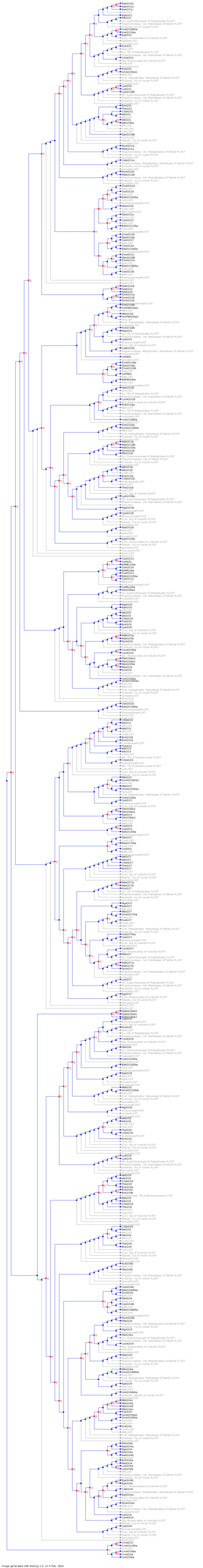

Supplement: Additional file 4: Figure S3. — Reconciled AGO gene family tree (GFT) with species tree showing details of duplication and loss events. The red internal node represents the duplication event while the blue node represents the speciation event. Grey color terminals are the lost nodes from respective taxonomic units. [file 12870_2014_364_MOESM4_ESM.pdf]

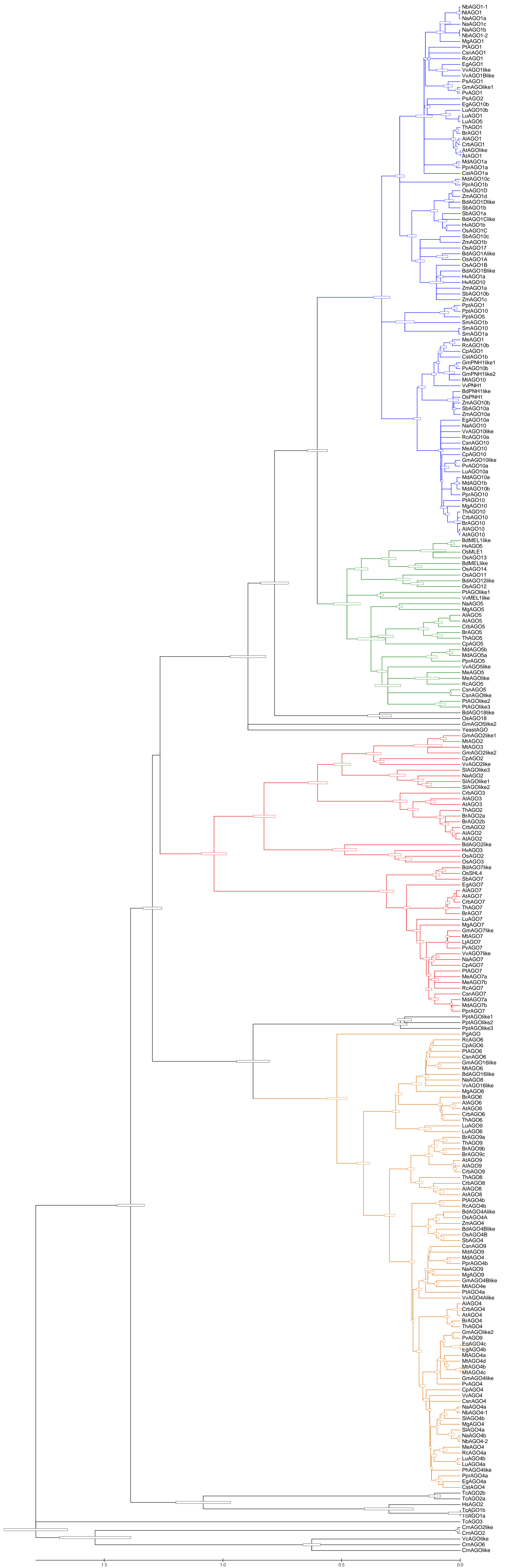

Supplement: Additional file 5: Figure S4. — Maximum likelihood (ML) tree of AGOs showing the approximate relative time of divergence of all the nodes using the molecular clock test on ‘plant AGO dataset II’. Small rectangular bars at the nodes in the tree represent the 90% confidence interval of the variance of the node height. The ‘scale’ (x-axis) is in million-years. [file 12870_2014_364_MOESM5_ESM.pdf]

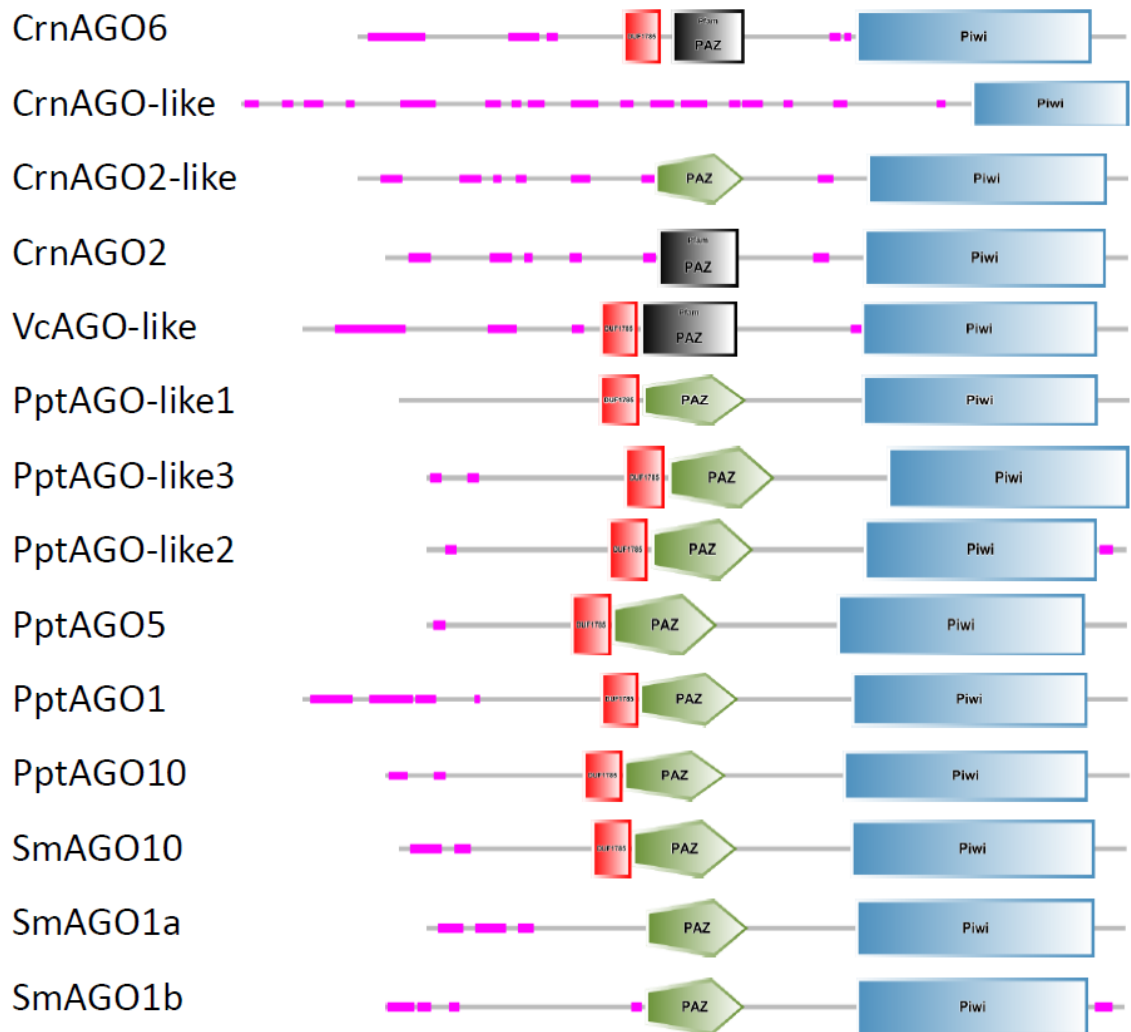

Supplement: Additional file 7: Figure S5. — Schematic representation of domain architectures in AGOs from lower plant groups. The positions of different domains were identified by sequence search of the SMART database. [file 12870_2014_364_MOESM7_ESM.pdf]

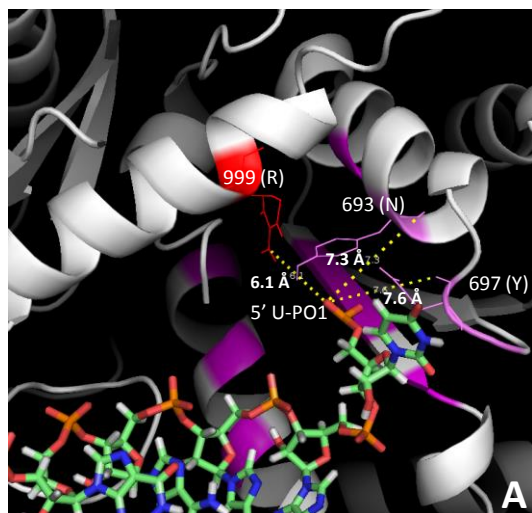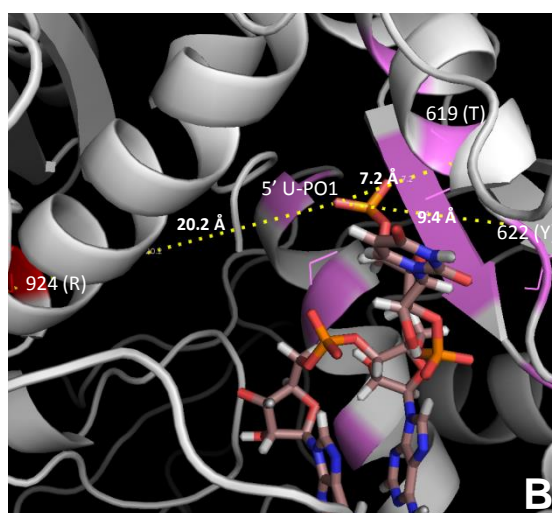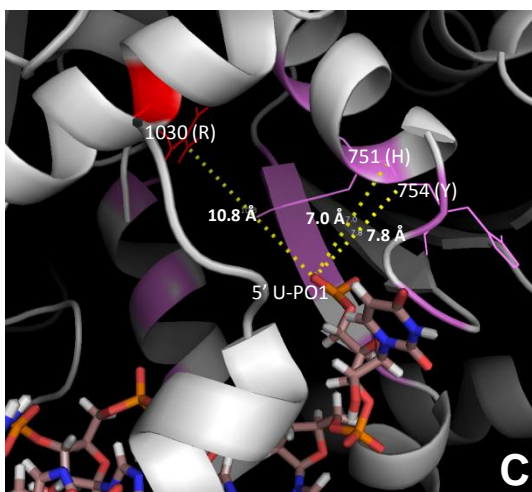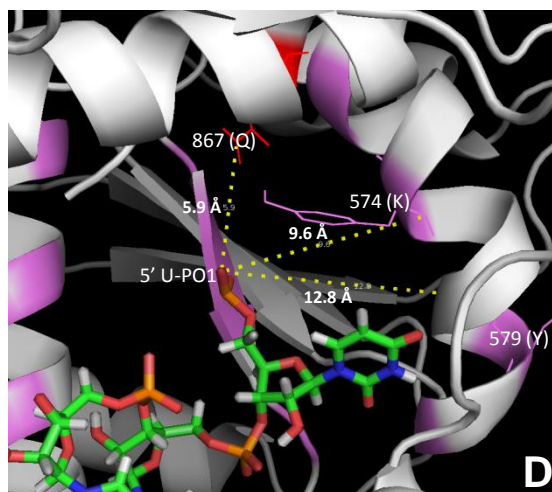

Supplement: Additional file 9: Figure S7. — Effect of variation in the residues in four classes on smRNA binding and stability with AGOs. Fig A, B, C and D show the interaction and position of the first oxygen atom of 5' P-U residue of RNA (PDB code 4F3T, chain R) in the lobe formed by MID-PIWI domain of NaAGO1a, NaAGO5, NaAGO2 and NaAGO4a, respectively. These docked structures were generated by using ClusPro server. The colored regions are the positions that interact and bind to the 5' P of smRNAs (red color on the PIWI domain and violet color on the MID domain). The first oxygen atom of the 5' P of smRNAs interacts with hydroxyl of 2nd and 3rd functionally important position of the MID domain and the 14th functionally important position of the PIWI domain. In NaAGO1a, the distance between hydroxyl of asparagine (N) at 2nd position and oxygen atom of the 5' P of smRNAs is estimated to be 7.3 Å, while in NaAGO4a, which has a lysine (K) at 2nd position, this distance is estimated to be 9.6 Å. This difference may affect the binding capacity and functional specificity of plant AGOs. [file 12870_2014_364_MOESM9_ESM.pdf]

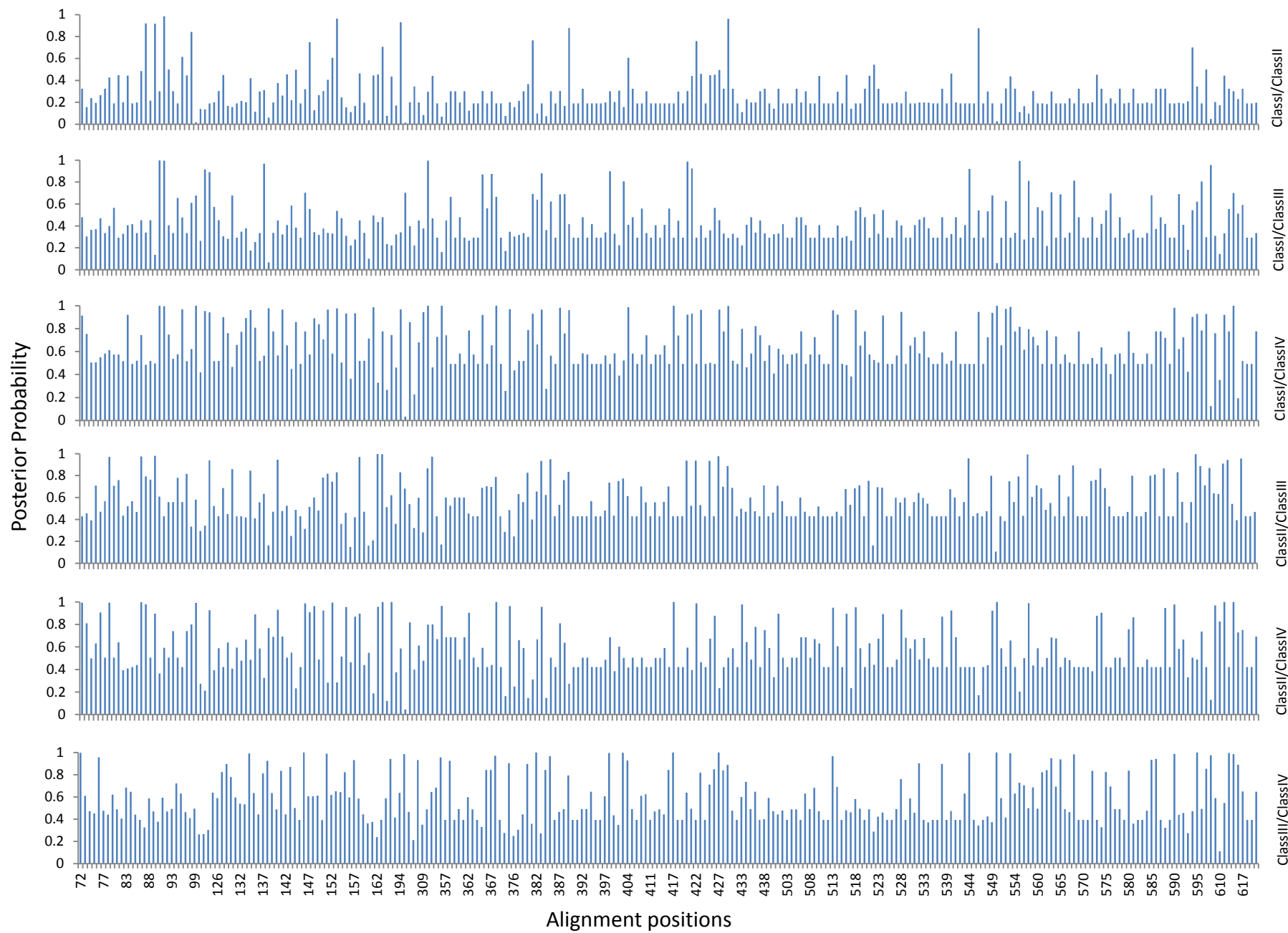

Supplement: Additional file 12: Figure S8. — The site-specific profile indicating critical amino acid positions measured by the posterior probability of being functionally divergent. X-axis represent the positions in ‘plant AGO dataset II’. Y-axis shows the posterior probability (PP). Result shows that there are 51 sites (PP > 0.9) that have strong probability of divergence between Class I and Class IV, whereas 36 and 38 sites (PP > 0.9) show strong probability of divergence between Class III and Class IV & Class II and IV, respectively. [file 12870_2014_364_MOESM12_ESM.pdf]

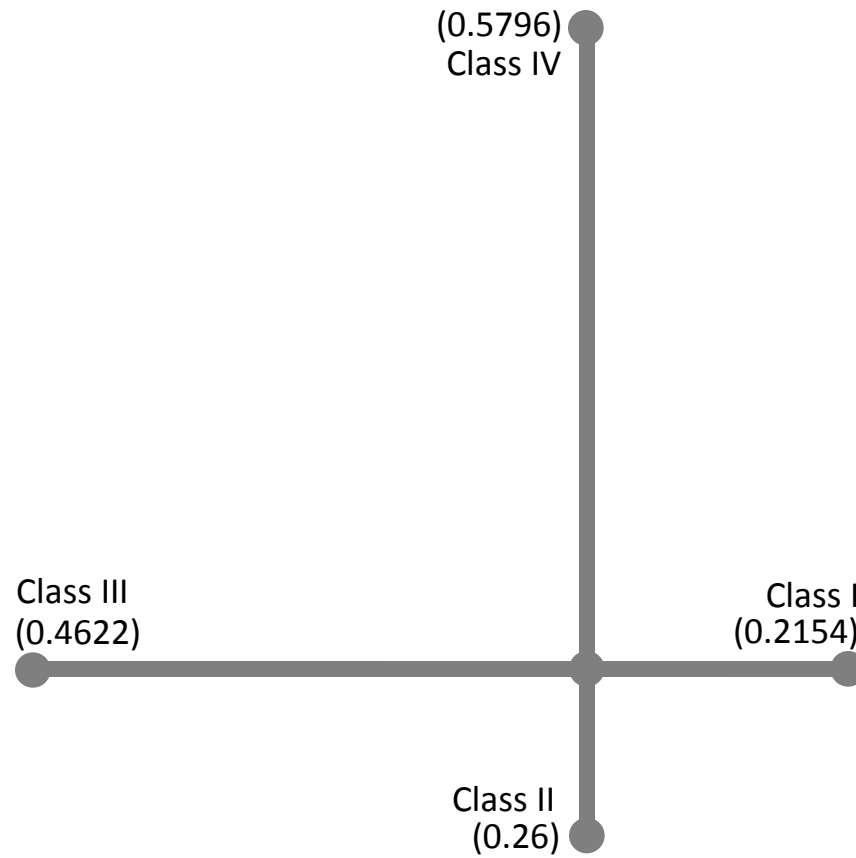

Supplement: Additional file 13: Figure S9. — Functional distance between four AGO classes in terms of tree-like topology. Class IV has longest branch length, indicating shift in largest number of sites from ancestor after duplication. [file 12870_2014_364_MOESM13_ESM.pdf]
