# Supplementary material for: Molecular evolution and diversification of the Argonaute family of proteins in plants
Source: BMC Plant Biol. 2015 Jan 28;15:23. doi: 10.1186/s12870-014-0364-6 (PMC4318128; doi:10.1186/s12870-014-0364-6)
Supplement: Additional file 6: Table S2. — Log-likelihood parameter of molecular clock test. [file 12870_2014_364_MOESM6_ESM.pdf]

Table S2. Log-likelihood parameter of molecular clock test.

	lnL	parameters	(+G)	(+I)
with clock	-83884.351	290	1.132	0.01
without clock	-81336.631	558	0.99	0.02
