# Supplementary material for: Molecular evolution and diversification of the Argonaute family of proteins in plants
Source: BMC Plant Biol. 2015 Jan 28;15:23. doi: 10.1186/s12870-014-0364-6 (PMC4318128; doi:10.1186/s12870-014-0364-6)
Supplement: Additional file 8: Figure S6. — Comparative analysis of the signature residues at functionally important sites in different domains of AGOs of lower plant groups as compared to higher plant. Amino acids at sites corresponding to signature residues of AGOs of higher plants are not found/substituted in lower organisms such as Chlamydomonas. [file 12870_2014_364_MOESM8_ESM.pdf]

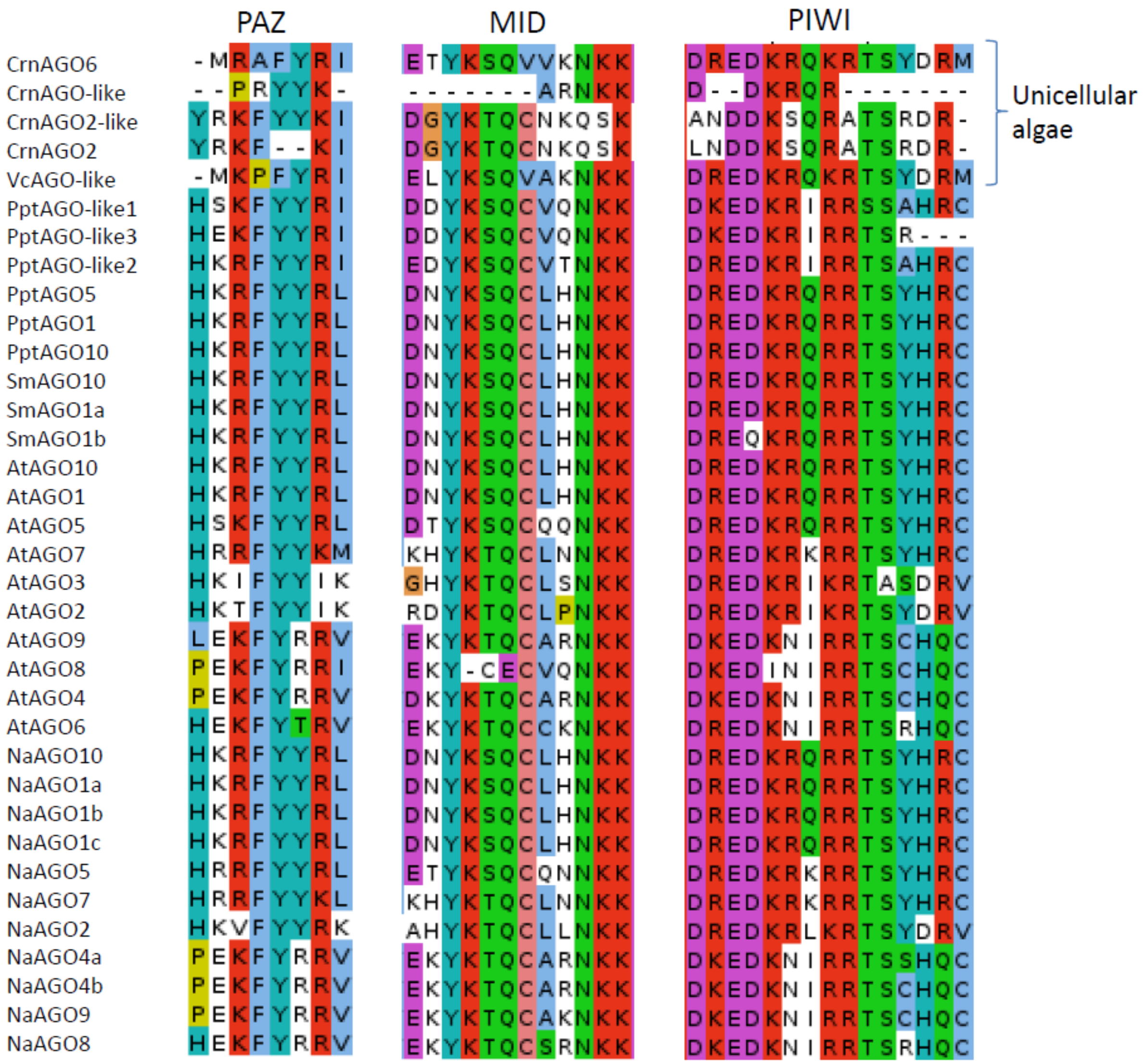
PAZ
MID
PIWI
Unicellular algae
CrnAGO6 -MRAFYRI ETYKSQVVKNKK DREDKRQKRTSYDRM
CrnAGO-like --PRYYK- -------ARNKK D--DKRQR-------
CrnAGO2-like YRKFYYKI DGYKTQCNKQSK ANDDKSQRATSRDR-
CrnAGO2 YRKF--KI DGYKTQCNKQSK LNDDKSQRATSRDR-
VcAGO-like -MKPFYRI ELYKSQVAKNKK DREDKRQKRTSYDRM
PptAGO-like1 HSKFYYRI DDYKSQCVQNKK DKEDKRIRRSSAHRC
PptAGO-like3 HEKFYYRI DDYKSQCVQNKK DKEDKRIRRTSR---
PptAGO-like2 HKRFYYRI EDYKSQCVTNKK DREDKRIRRTSAHRC
PptAGO5 HKRFYYRL DNYKSQCLHNKK DREDKRQRRTSYHRC
PptAGO1 HKRFYYRL DNYKSQCLHNKK DREDKRQRRTSYHRC
PptAGO10 HKRFYYRL DNYKSQCLHNKK DREDKRQRRTSYHRC
SmAGO10 HKRFYYRL DNYKSQCLHNKK DREDKRQRRTSYHRC
SmAGO1a HKRFYYRL DNYKSQCLHNKK DREDKRQRRTSYHRC
SmAGO1b HKRFYYRL DNYKSQCLHNKK DREQKRQRRTSYHRC
AtAGO10 HKRFYYRL DNYKSQCLHNKK DREDKRQRRTSYHRC
AtAGO1 HKRFYYRL DNYKSQCLHNKK DREDKRQRRTSYHRC
AtAGO5 HSKFYYRL DTYKSQCQQNKK DREDKRQRRTSYHRC
AtAGO7 HRRFYYKM KHYKTQCLNNKK DREDKRKRRTSYHRC
AtAGO3 HKIFYYIK GHYKTQCLSNKK DREDKRIKRTASDRV
AtAGO2 HKTFYYIK RDYKTQCLPNKK DREDKRIKRTSYDRV
AtAGO9 LEKFYRRV EKYKTQCARNKK DKEDKNIRRTSCHQC
AtAGO8 PEKFYRRI EKY-CECVQNKK DKEDINIRRTSCHQC
AtAGO4 PEKFYRRV DKYKTQCARNKK DKEDKNIRRTSCHQC
AtAGO6 HEKFYTRV EKYKTQCCKNKK DREDKNIRRTSRHQC
NaAGO10 HKRFYYRL DNYKSQCLHNKK DREDKRQRRTSYHRC
NaAGO1a HKRFYYRL DNYKSQCLHNKK DREDKRQRRTSYHRC
NaAGO1b HKRFYYRL DNYKSQCLHNKK DREDKRQRRTSYHRC
NaAGO1c HKRFYYRL DNYKSQCLHNKK DREDKRQRRTSYHRC
NaAGO5 HRRFYYRL ETYKSQCQNNKK DREDKRKRRTSYHRC
NaAGO7 HRRFYYKL KHYKTQCLNNKK DREDKRKRRTSYHRC
NaAGO2 HKVFYYRK AHYKTQCLLNKK DREDKRLKRTSYDRV
NaAGO4a PEKFYRRV EKYKTQCARNKK DKEDKNIRRTSSHQC
NaAGO4b PEKFYRRV EKYKTQCARNKK DKEDKNIRRTSCHQC
NaAGO9 PEKFYRRV EKYKTQCAKNKK DKEDKNIRRTSCHQC
NaAGO8 HEKFYRRV EKYKTQCSRNKK DKEDKNIRRTSRHQC
