# Supplementary material for: Molecular evolution and diversification of the Argonaute family of proteins in plants
Source: BMC Plant Biol. 2015 Jan 28;15:23. doi: 10.1186/s12870-014-0364-6 (PMC4318128; doi:10.1186/s12870-014-0364-6)
Supplement: Additional file 10: Table S3. — Relative evolutionary rate in plant AGOs sequences. (A) Site-specific relative evolutionary rate of AGOs across Classes. Orange: sites that show high relative rate (>1) across all classes. Sky blue: sites showing low relative rate (<1) across all classes. (B) Comparative summary of relative rate across four AGO Classes. [file 12870_2014_364_MOESM10_ESM.pdf]

Table S3. Relative evolutionary rate in plant AGOs.

(A) Site-specific relative evolutionary rate of AGOs across Classes. Orange: sites that show high relative rate (>1) across all classes. Sky blue: sites showing low relative rate (<1) across all classes.

Site number in dataset II	Class I	Class II	Class III	Class IV
1	0.371446204	0.107456362	0.231259924	0.077060449
3	0.446950293	0.133428082	0.294633827	3.196170876
4	2.974092211	1.411464924	0.361348475	0.823092522
5	4.31170862	3.328584819	3.046088218	3.196199235
6	0.160188199	0.149776962	0.338235918	0.801141601
7	4.311511552	2.772846907	0.820364929	3.072508342
8	0.160188199	0.149776962	1.404781032	0.099752617
9	4.311711402	3.330420453	3.05113281	3.196168004
10	4.305720432	1.125955191	2.983999832	3.195905205
11	0.958705343	3.216934015	0.929439811	0.577511644
13	4.311537129	3.328590161	2.975483014	3.196199238
14	0.472645518	1.214422579	0.278266231	0.216237994
15	0.848862315	0.76214312	1.67989952	2.683249165
16	0.684657487	0.092428968	0.255527312	1.156480271
17	0.277602554	0.092771564	0.066405101	0.07413575
18	0.724822333	0.091481967	0.066505165	0.214499887
19	0.474881768	0.132094457	0.284325773	0.747196224
20	4.289746284	1.816264215	2.852800167	2.146827698
21	1.271947897	0.091481045	0.067124249	0.138566976
22	4.309363812	3.216758341	3.049690001	1.804583899
23	2.511232891	1.213343193	1.006313959	1.238923947
24	0.851798935	1.327380606	0.408995965	1.340465064
25	3.568561341	0.915720955	0.712896411	2.94679834
26	4.311693795	2.48765693	2.910122863	1.037047504
27	4.099045433	1.999527051	0.659908966	1.517476837
28	0.466968942	0.12838694	1.006622913	0.575283375
29	0.489609705	1.572402323	1.232934217	3.183504371
30	4.311706733	3.330270818	3.024703082	2.131380647
31	4.311575944	3.328954651	1.195424493	0.073709878
32	4.216014149	1.195992167	1.881899653	0.232197271
33	4.201683758	3.055258814		
34	4.311711417	3.330024956	1.669441096	3.19619828
35	0.999760111	1.122521311	3.051241378	2.184682275
36	2.974324596	1.296589251	3.051219635	0.968149135
37	0.77299745	1.195665083	2.417973531	0.825796174
38	2.453728382	1.70344458	0.693568746	3.196021915
39	4.31171137	3.328310981	2.306104344	2.928163765
40	1.058751542	0.933324611	1.83159272	0.443802908

41	1.26337001	0.246291935	0.35303225	0.141186227
42	0.152455864	0.142015131	1.104180106	0.231633836
43	0.347969902	0.110071092	0.223989301	0.079546243
44	0.528783601	0.148759168	0.085034274	0.099497903
45	1.096147475	1.258467263	1.329358536	0.861642841
46	0.88191027	0.300572994	1.558738779	0.297611956
47	1.128388965	0.922258575	0.534734165	1.20030986
48	0.469530198	3.210643652	0.835629557	0.088993779
49	0.50723023	0.71969678	1.226501933	0.423765863
50	0.249416814	0.089528083	0.307466451	0.13307096
51	0.895746895	0.091934227	1.326228154	3.034455961
53	0.852494443	0.148759168	1.265386362	0.272687239
54	4.215062921	3.222937906	2.689744929	3.012637204
55	0.144249189	0.12838694	0.41954392	0.222209135
57	1.05896682	0.414554523	1.155061787	0.672120233
58	0.479490688	0.232783459	2.186688066	0.136653012
59	4.277025565	1.070272148	0.702345294	2.005075238
60	0.836902886	0.662419914	1.219101752	0.759619861
61	0.586386654	0.929077554	1.625793642	2.095940887
63	1.816060736	3.324248673	1.100571983	3.177878223
64	2.180675728	0.231760686	0.719069475	0.456004724
66	1.265899046	1.303313612	0.639969354	1.194528809
68	1.980765202	2.406897031	1.303391554	0.923303484
69	1.050728946	3.222561077	1.228874322	0.302416753
70	1.381682745	0.732790009	2.837916959	0.229127402
71	1.187130588	1.29498172	0.909202334	1.077744833
72	0.435667298	0.713241499	0.290349557	3.162498602
73	0.788497088	1.425987669	0.471097424	0.498831416
74	0.38991368	2.451943502	1.232776614	0.371010278
75	0.705746565	1.032611624	0.986880379	0.462216837
76	2.632972769	0.366649682	0.759077185	2.943200767
77	1.424256464	0.49370427	0.25603081	0.161766248
78	1.415445552	2.682535812	0.272146965	0.19382776
79	0.990091184	0.12838694	0.490738595	0.370874613
80	0.110893775	0.543932365	0.220018365	0.169581831
82	0.429143743	0.610359759	0.701417214	0.861974155
83	0.273303621	0.78321596	0.54230083	1.149666536
84	0.466930382	0.133234659	0.584128258	0.088980773
85	1.067382052	0.541938029	0.229739427	0.568761849
86	0.944632672	1.467035261	0.233251134	0.077130422
87	1.312710467	3.325476054	1.021759429	1.169312199
88	1.311219069	0.940720529	0.380786867	0.582695375
89	3.805947957	0.225050265	2.401010805	0.524436087
90	4.31170793	3.319045262	1.270158821	1.213928794

91	3.411866441	0.388177511	0.083627312	0.501676799
92	1.144488151	0.317953485	0.427790125	0.158185768
93	1.219473073	0.225166413	1.199368009	0.445843213
94	1.256807919	0.136167449	0.969342071	0.251054259
95	1.379636702	0.423775545	0.757993736	0.15602678
96	0.425910454	1.222506771	0.774411723	0.614188723
97	0.757302544	3.328440325	1.346905913	0.837257115
99	4.311703454	3.330407457	1.829803365	0.915834683
100	1.940203143	1.864275313	2.777012722	2.799720479
101	2.092132032	0.163052305		3.195565813
102	4.311710982	3.190260229	1.798297562	3.152653736
103	0.785809547	0.296102342	1.623509357	1.381752297
104	1.541398638	0.520758555	2.999004003	0.889048656
105	4.310782547	1.59428552	2.979643734	1.537509564
106	0.917892806	0.151828492	0.717039752	0.276599974
107	1.062004214	3.158778879	1.154739957	0.523566437
108	2.558600779	1.670528763	0.893773768	0.824358101
109	0.159021796	0.151828492	0.083627312	0.165883199
110	0.135770396	1.664515851	0.637425933	0.916550665
111	0.819183044	0.73485191	1.129560351	0.144687011
112	0.548093684	0.105162409	0.383281628	0.135500544
113	0.478962204	0.088727125	0.659592907	0.135436649
114	0.246981991	0.384076387	0.075049911	0.156630368
115	0.591034536	0.105228391	0.223680405	0.250507665
116	0.775637437	0.974192434	1.551414424	1.19520935
117	0.252943733	0.409665818	0.066331746	0.42988147
118	0.313667482	0.320695298	1.655187671	0.835582399
119	0.517562378	0.457477898	0.083627312	0.165883199
120	0.141503473	0.385363853	0.275785497	0.22262266
121	0.49747756	0.206112171	0.979352339	0.311746889
122	0.141503473	1.213300958	0.075049911	0.148109119
123	0.293252529	0.238369116	0.29928294	0.190184023
124	0.434669167	0.379678022	0.954533196	1.1241569
125	0.278350447	0.760511874	1.066709296	0.515727082
126	0.429644987	0.209086741	0.065779415	0.181701756
128	0.752278037	0.571589342	0.697981094	0.133238543
129	0.619987058	1.028250053	1.125236535	0.133125462
130	0.49185377	1.187996456	0.774715887	0.919849961
131	0.135678165	0.129124767	0.800918094	0.438166056
132	0.237855863	1.206564146	0.834239147	1.22506288
133	0.386924789	0.729071298	0.679588022	3.154945534
134	1.588256173	0.56108857	1.281091387	0.275339518
135	1.482655883	2.959475118	1.462209803	0.691392158
136	0.260688127	0.227313838	0.562893261	0.19060074

137	1.568366674	1.343248973	0.696232757	1.208084998
138	4.31161584	3.297738016	3.028437512	0.770924694
139	1.451035189	0.667786904	1.07584326	0.094006653
140	0.663684381	1.048898674	0.281693723	0.222575721
141	1.014523375	0.940777357	0.588432021	0.074908944
142	0.109160785	0.528082103	1.334013021	1.011539999
143	0.606429351	1.108790815	3.044353948	1.162261148
144	0.669163794	1.067815597	1.232069813	1.444087524
145	0.45862966	0.131005084	0.075049911	0.236274157
146	0.689696175	1.530870141	2.371347655	0.173394579
147	0.158914733	1.272533887	0.61510569	0.328296122
148	0.686803305	1.136772156	0.78025316	0.671159527
149	0.81049575	0.953982771	0.690024748	0.154157819
150	0.260245604	0.621478404	0.516754662	1.15403619
151	0.405022179	2.410443157	0.551562784	3.19483874
152	0.6133277	1.512489187	0.304298969	0.188568759
153	0.152455864	2.529771853	0.786810163	3.168978485
154	0.328326791	0.732329019	1.245643776	0.444085007
155	0.434072028	0.524333226	0.51618908	0.074105876
156	1.324094296	1.667765718	3.050118059	1.344391159
157	0.905885158	1.502348854	2.732864862	0.101100599
158	0.775141123	0.876957719	0.273360461	0.557714353
159	0.28406196	0.732532609	0.352880282	0.186483131
160	3.45205213	3.245877069	3.049258111	3.188344412
161	0.546419363	3.313428007	3.04910143	3.196199055
162	1.690011483	0.688307799	3.05110692	3.196186577
163	0.253253329	3.280616723	0.199177288	0.105478759
164	1.40555187	3.186604184	1.336090657	3.195539741
165	0.440926354	2.311364331	1.603899724	3.043753631
166	0.490487548	0.526647919	1.368684742	0.186776008
167	0.10373848	0.090345528	1.050969595	0.194454285
168	1.57148292	1.788639032	2.777968658	2.715943016
169	1.461829127	0.295144868	3.042415036	3.190375977
170	0.175814139	0.139740837	0.275630837	0.092256235
171	0.156941976	0.395419403	0.430933503	0.762633744
172	0.744311241	3.276409501	1.863901046	3.164433007
173	0.744039784	1.455788878	1.084642779	3.195515093
174	0.175814139	0.139740837	0.273578619	1.222171023
175	2.19100028	0.728957579	2.748763066	3.196199013
176	1.056892907	0.81922581	2.19269831	0.76534502
177	4.302942786	2.72962897	1.356342518	3.196199238
178	0.085082791	0.366970865	0.065411012	0.567194701
179	3.074696141	1.827857625	2.320373427	3.196013712
180	1.373120348	3.154114656	2.197986591	1.777713107

181	1.139239656	1.027458366	0.910565072	2.941635638
182	0.138433279	0.130434926	0.077393665	0.239868041
183	0.166099874	0.863699256	0.686918714	0.107223856
184	0.141406262	0.812349543	0.075049911	0.489527946
185	1.352900516	0.693201615	0.803576213	0.357634834
186	0.091177869	0.878407816	0.994761692	0.204512775
187	0.521999261	0.146389366	0.504615597	0.100996338
188	0.488821446	0.848137577	0.775590942	0.654077975
189	4.264000838	0.997374119		0.687313819
191	4.061800345	1.096235657	1.257652674	0.79413041
192	1.143906408	1.742825916	0.693750834	0.701989543
194	2.417776504	0.130434926	1.794500981	0.392831093
195	0.492292079	1.190293855	1.339930372	1.897236215
196	0.143101597	0.637216136	0.216723064	2.991348077
197	0.140070084	0.130434926	0.077393665	0.230760473
198	0.486156031	0.089975726	0.196007322	3.196192719
199	0.1204266	0.110177698	0.070392274	0.082711268
200	0.274802784	2.287436916	1.114375708	0.83902883
201	0.16925237	0.459548182	0.084757509	0.299237497
202	0.111234805	3.289132239	3.049284829	3.196178507
203	0.241025991	0.084310723	0.490951443	0.380589229
204	1.120730578	1.222704433	1.262235735	1.071657256
205	1.604678049	3.239686233	0.233140465	0.788469679
206	0.161970138	0.744404928	0.347622619	0.220672126
207	0.105415338	0.101804811	0.208114264	0.245740411
208	0.356921677	0.105228391	0.582522046	0.076965862
209	0.476631058	0.716141504	1.050821443	0.231841882
210	0.913601555	1.219312821	0.633819511	0.310276486
211	0.591457566	1.646977379	0.660230203	0.174964345
212	0.888118766	0.887417733	0.798398644	1.154620896
213	0.311810719	0.101804811	1.081015699	0.156504989
214	0.448258735	0.22405196	1.014220898	0.260098705
215	0.864223311	0.387953926	2.926641059	1.201115894
216	1.300619002	3.245722143	3.036815909	1.122540991
217	0.460304183	1.150775865	0.640726105	0.223673596
218	0.142554433	0.126361819	1.247316671	0.44317156
219	1.534206773	1.046212044	2.903048683	0.751494834
220	1.125325345	0.827854173	0.878133849	1.168653097
221	0.458719368	0.688326124	1.082765776	0.298861893
222	0.557199244	0.471090671	1.257367016	0.252614316
223	0.547911051	0.879638521	1.286532605	0.253180906
224	0.874514878	0.531108826	2.961894005	0.813146269
225	0.1397679	0.130434926	0.447130013	0.089500785
226	4.311710881	3.330427679	3.040749422	1.689300939

227	4.309403192	3.327042982	1.555617111	1.20600879
228	1.241513246	0.105228391	0.067791634	0.594807183
229	0.247398169	1.307718004	0.856053025	0.83115279
230	1.244134619	1.065299695	0.526490739	1.214261629
232	4.311711326	3.329168598	3.048839824	3.195109532
233	3.447618623	3.330422028	1.089037888	3.196199206
234	0.094836279	0.946751002	3.051241233	1.199313194
235			0.847067264	
236	4.280647623	3.282796766	0.342245912	3.168076214
237	0.15289214	2.263195149	0.624542081	0.392168571
238	4.311711404	3.330442595		3.158030327
239	0.337234406	1.216394175	0.94317212	1.200261701
240	1.56720044	3.330443783	3.049459717	1.277980229
241	0.468238628	3.328069814	1.644966105	1.208468163
242	0.094662792	1.336718911	2.77659596	0.381624157
244	4.089563872	3.327170412	2.894722411	1.473200769
245	0.119360525	0.81810466	0.72044196	2.453509259
246	0.136064287	0.129124767	0.076126768	1.238290225
247	0.521728071	0.459384304	2.119847067	0.273495409
248	0.767538625	0.696057409	0.902922263	1.086219021
249	3.173661918	3.236412195	3.040734273	0.890196526
250	0.236035764	0.583460797	0.562908309	0.295566752
251	1.211585727	3.325753033	0.818142305	3.029535954
252	4.309483969	3.283220526	2.577806876	3.196139881
253	4.310501542	2.125416114	1.956020303	3.196198746
254	0.774643559	1.029628929	0.066144604	1.111508772
255	1.270034689	1.340320947	0.19969403	1.193436615
256	1.478555158	3.328437143	3.049107252	1.428691419
257	0.66051243	1.138955024	0.590406639	1.203136842
258	0.939190145	1.270163606	3.050239432	3.004512765
259	0.283772114	0.091934227	0.336855273	0.09960581
260	0.108684193	0.106780675	0.067791634	0.154682521
261	1.413994321	0.22926297	1.142670404	1.188864769
262	0.143436986	0.12838694	0.362515136	0.089074783
263	0.140392988	0.684918391	3.036727899	3.19486353
264	1.841495149	2.076339573	0.833986165	1.20713565
265	0.140392988	0.132527644	0.077393665	0.089595909
266	4.297298546	1.545721877	2.885058057	2.620653205
267	0.14301942	0.126361819	0.075049911	0.089662567
268	0.110366468	0.696488195	0.89393713	0.962605808
269	0.158914733	0.146389366	0.596040293	0.464142593
270	1.382403713	0.101804811	0.498957953	0.499817918
271	0.23971568	0.215148861	0.766578425	0.149488528
272	0.095577206	0.091130385	0.324983328	0.30571775

275	0.091092489	0.245086578	1.073912291	0.590278753
276	1.074088059	0.761872216	3.040927533	0.883537787
277	0.489824278	1.047723147	2.954835706	1.215979642
278	0.109160785	0.519408943	0.756687964	0.550379345
279	0.578449816	0.294947451	1.309318168	3.183860396
280	0.830131167	0.243322863	0.700042871	3.107466184
281	0.964456563	1.90415946	1.887285828	3.191399283
282	0.665203647	0.413131443	0.859981537	3.196197289
283	0.159070187	0.456959746	0.335561145	1.385301106
284	1.010900199	0.871273264	1.079885508	3.196197258
285	4.304303222	3.32615773	3.020400664	3.109253424
286	0.470480209	0.722747073	0.878035995	1.109101528
287	4.310261084	3.301061936	1.08616057	3.190297102
288	4.310081389	3.328358953	1.136805626	3.196177981
289	0.239923571	0.215148861	0.111138699	0.149270562
290	4.231349277	0.849105062	0.426247407	0.747664158
291	0.551750046	0.759725854	0.993949861	0.791194116
292	1.496492377	1.824851557	1.467393863	0.441426251
293	0.274055393	1.230401234	0.296610393	0.185520363
294	0.135812201	0.129124767	0.278029065	0.094006653
295	0.450786683	0.366410617	0.404451463	0.073839518
296	1.1171355		0.770410407	1.047423534
297	4.311639411	2.913901774	3.011076221	1.180856261
298	4.311711418	3.330331954	3.051049808	2.124399944
299	1.383944544	3.293673169	2.716866819	1.266747115
300	0.459116538	0.126361819	0.541518585	0.676170301
301	1.167484149	3.21062265	1.633489413	2.160748218
302	4.299832639	3.330357133	3.036317216	3.16224327
303	0.159070187	0.146389366	0.481612887	0.099481825
304	0.090255001	0.224783572	0.560219331	0.891008562
305	4.178522509	3.330363866	3.017765274	3.196199239
306	0.43624675	1.841623463	0.407942485	1.197110419
307	1.95021349	1.329102904	2.953338357	3.185414021
308	1.135680041	0.539817603	1.892877325	1.284650576
309	2.933535729	3.322187962	1.77379688	3.057699482
310	0.87015597	1.250722134	0.812300765	0.074634575
311	0.141406262	0.597517013	0.075049911	1.734785397
312	1.509932807	3.32954866	2.138485084	2.617797563
313	4.311635423	1.114847516	3.037737817	3.196199239
314	1.255141485	1.065397757	0.463457419	3.088741685
315	1.329422926	0.482301532	0.269209307	3.196181578
317	0.141406262	0.842107903	0.775442556	0.774755243
318	0.46224812	0.126361819	1.108450607	1.201316089
319	1.334765919	0.351276572	1.335395498	0.390079623

320	1.451066112	0.911453121	0.084757509	0.29169352
321	0.430897941	0.194837282	1.282478239	1.148394948
322	0.141406262	0.126361819	0.066144604	0.541078209
323	0.138433279	0.663778602	1.164701539	1.003617093
324	0.335689679	0.921833988	3.049281737	1.102635665
325	1.102500188	1.278526409	1.147013029	1.132723
326	0.094587279	2.204023198	1.00701342	0.178304453
327	0.158914733	0.993826871	1.787485316	0.193082474
328	1.349793081	0.89242534	0.083627312	1.235168259
329	1.530040969	0.354613914	0.075049911	0.150342385
330	0.109160785	0.105739541	0.069876379	0.080488756
331	0.567787886	0.513271387	0.708685895	2.612062699
332	0.616534052	1.39520615	0.41994656	1.215884157
333	0.166099874	0.150654156	0.827422104	1.246267426
334	0.372426403	0.110870602	0.233023429	0.231788248
335	0.442372216	0.218712741	0.066331746	1.951059953
336	1.829874884	0.750278781	1.757123587	3.186264168
337	0.462415094	0.64759756	1.290340237	3.196199239
338	0.158914733	0.467239009	0.083627312	0.282632036
339	4.24945723	0.540996466	0.896108481	0.441628619
340	0.535011338	0.407410627	0.797784356	1.197735407
341	0.420112178	0.373371135	0.599866549	0.510201692
342	0.103493028	0.102404561	0.067420073	0.158607027
343	0.166099874	0.150654156	0.084757509	0.105365168
344	0.166099874	0.486926711	0.754784436	1.191239241
345	1.117164283	0.984792622	0.075049911	1.158688568
346	4.269660328	2.70378951	3.011820799	0.619426971
347	0.822430546	3.072975608	0.703052925	0.427653637
349	0.386897312	0.595508753	0.208390738	0.498794972
350	0.464942266	0.460762461	0.421331678	0.090952169
351	1.054427245	1.687355441	1.393050829	1.504246905
352	0.274481125	0.114789682	0.881073417	0.510158313
353	0.274324524	0.094313523	0.197489927	0.074081715
354	0.911072	1.003689431	0.272016517	0.970714432
355	1.330202136	1.08123222	0.37030898	0.220086164
356	0.141406262	0.131929571	0.38588319	0.377771688
357	0.333150836	0.308482108	0.069876379	0.080194135
358	0.085082791	0.372572571	0.530643878	0.133997991
359	0.094587279	0.244398436	0.066264682	0.074081715
360	0.264689733	0.241027366	0.259890504	0.455030722
361	0.109160785	0.312795763	0.069876379	0.080194135
362	0.488274797	2.2468887	1.364156866	0.56453287
363	0.158914733	0.457072118	0.083627312	0.274335456
364	0.158914733	0.478712939	0.083627312	0.099481825

365	0.107197691	0.912810474	0.996378155	1.210310252
366	0.094587279	0.227729181	0.239334938	0.074081715
367	0.087576163	0.684430999	1.162256982	0.180489053
368	0.091119602	0.571087777	0.884785998	3.196191001
369	0.141406262	0.384455325	0.075049911	0.088980773
370	1.484203764	3.041661668	2.790566818	2.893698971
371	0.339296004	0.804940954	1.209357572	3.150433919
372	0.094412722	0.416198988		0.186102536
373	4.302186281	1.188329534		3.195951454
374	1.473942867	3.32744406		1.210095399
375	2.774797488	1.215195198		3.196184164
376	1.372801019	2.57623699	1.451574162	3.060478515
378	0.640339197	0.52052266	0.774906767	1.138370972
379	0.446812409	0.132527644	0.393184632	0.682036826
380	1.111285729	3.301630489	1.059417681	3.19506121
381	0.091119602	1.546714005	1.473442207	0.652923669
382	0.821021972	1.236007349	3.051229767	0.923850571
383	0.110893775	0.540694649	1.920118439	1.217690979
384	1.739837528	3.328630788	2.130589294	3.140972958
385	0.445466985	0.413570986	1.71067554	0.233646642
386	0.087576163	0.089528083	0.430403696	0.073706934
387	0.085082791	0.368133784	0.313177464	1.223770309
388	0.429427572	0.569812616	0.076126768	0.134056696
389	0.135678165	1.328245193	0.365418723	1.195423604
390	0.158914733	0.148759168	0.083627312	0.099481825
391	0.094412722	0.091934227	0.066493222	0.190911027
392	0.369176086	0.110071092	0.068807153	0.172748055
393	0.091119602	0.22714679	0.066940746	0.136045523
394	0.245538651	0.215241883	0.199479843	0.073839518
395	0.08901721	0.089901689	0.066359393	0.075142834
396	0.138433279	0.401648783	0.077393665	0.276092945
397	1.161074548	1.261170177	0.909680537	0.687672793
398	0.158914733	0.46120493	2.552702122	0.275440037
399	0.665146796	0.583340553	0.651223485	1.372227175
400	1.127280264	0.30828927	2.292338922	1.164758575
402	1.304414913	0.902673394	3.049818079	0.538782941
404	0.791410196	0.856321435	0.502601215	3.001808327
407	0.435667298	0.610788404	0.293091808	0.384554729
408	0.083956901	0.0864428	0.065779415	0.073839518
409	0.085082791	0.084310723	0.217702348	0.752163886
410	0.273749471	0.090798824	0.517981039	0.141245383
411	0.094412722	0.090798824	0.066493222	0.074908944
412	0.091119602	0.229376938	0.202946552	0.136045273
413	0.262347231	0.090345528	0.066940746	0.136072449

414	0.273456972	0.090798824	0.307133286	0.376856473
415	0.229021153	0.0864428	0.23825081	0.073839518
416	0.310346538	0.089975726	1.317659326	0.678732366
417	0.110893775	0.108439796	0.241169838	3.196194112
418	0.79664291	0.215148861	0.563439268	0.149270562
419	0.138433279	0.130434926	0.639752584	0.089500785
420	0.119024316	0.65312788	1.333322318	1.443726289
421	1.402899907	0.565410016	0.337309726	0.553170132
422	0.087576163	1.035720469	0.236038204	0.13430008
423	0.3330728	0.691200661	0.626919166	1.1795003
424	0.152455864	0.139740837	0.075453836	0.090952169
425	0.48418307	0.512684729	0.735970508	0.659801663
426	0.158914733	1.258731963	0.529959749	0.141629677
427	0.458873973	2.333599954	0.067791634	3.024296894
428	0.256405708	0.090345528	0.486034036	0.136077168
429	1.678792935	0.0864428	1.006412472	0.383226911
430	0.278397958	0.410538396	0.631507394	0.498944416
431	0.103493028	0.101804811	0.067420073	0.077130422
433	1.289679678	3.22641336	3.03488742	0.508032117
434	0.712422447	0.405235298	0.491486953	2.470156379
435	0.328326791	0.292264214	0.211446127	0.434399168
436	0.767794557	0.967770217	1.204916414	1.607349624
437	0.374852913	0.110177698	0.070392274	0.081888298
438	0.819376684	0.084310723	0.820040918	0.444900148
439	0.085082791	0.084310723	0.065411012	0.43322392
440	1.017923857	1.32763764	1.353913432	1.026491855
441	0.369913402	0.52912917	1.352485417	1.15536256
442	0.141406262	0.406017909	0.454601213	0.221845281
443	4.29447521	0.749373466		3.049740761
444	1.3546463	2.956954107		0.665048579
445	0.648849264	0.114265029	0.483612638	0.472369172
446	0.847859009	1.062311451	0.594574038	1.231251514
447	0.144455294	2.523465057	0.794657224	0.392256221
448	1.625960177	2.629623957	3.04847882	3.196135256
449	0.580686194	0.585700836	1.597943168	0.316646165
450	0.445407557	0.13419299	0.730572831	0.252347115
451	1.331419064	3.264354907	3.051199567	
452	4.197723238	3.330348823	1.276860928	1.303861086
453	0.346436971	0.733850491	0.556631003	0.441896726
454	0.468886474	0.135562324	0.077393665	0.090082224
455	4.14987372	3.330365123	3.012566017	3.196132216
456	0.332097544	0.109021464	0.746469169	1.047680476
457	0.240762538	0.534837267	0.189037908	0.303425052
458	0.607766679	0.205879088	0.692495295	0.433341256

459	0.442406945	0.13419299	0.783916587	0.860161858
460	0.506494727	0.994858856	0.076126768	0.094673718
462	0.107517625	0.109021464	0.067791634	0.076965862
463	0.362136114	0.112425832	0.068807153	0.176808736
464	0.519867187	0.152180724	0.083627312	0.277449669
465	0.27028257	0.240737333	0.066940746	0.329815691
466	0.083956901	0.222097298	0.065779415	0.307589522
467	0.386924789	0.334129748	0.632550843	0.469357595
468	0.519867187	0.477246095	1.378239953	0.134428101
469	0.106689074	0.307713028	0.207697995	0.157127413
470	0.460222139	0.13419299	0.076126768	0.094006653
471	1.034414804	1.102821732	2.714872215	1.18839491
472	0.106689074	0.897545174	0.205184115	0.388150182
473	0.276400143	0.093102818	0.250548796	0.074410843
474	0.145987275	0.131300739	0.408055799	0.538398845
475	0.778978987	0.402683087	1.318735687	0.444385779
476	4.31167793	2.931250139	0.639122463	1.170072897
477	0.122822184	0.114265029	0.070392274	0.082544567
478	0.145987275	0.413505332	0.411297249	0.837464377
479	0.915252157	1.172958654	3.046041775	3.179161126
480	0.097251164	0.240341763	1.214133907	0.398737691
481	0.087511027	0.086540231	0.753764217	0.440053743
482	0.325967345	0.532526505	1.357626293	0.556371033
483	1.149842247	1.044872298	3.051241866	1.084729184
484	0.094412722	0.090798824	1.425971799	0.144383959
485	0.166099874	0.823914723	0.954511713	0.474547935
487	0.515536785	1.183800087	1.713467228	0.648124593
488	0.230135891	0.363116832	3.010208292	1.665655374
489	0.445164108	0.384076387	3.050631269	0.892036496
490	0.403192303	1.553153505		0.609781844
491	2.519452514	1.017822631		0.864255963
492	4.047515806	2.755755676	2.989679187	3.196199218
493	0.152455864	0.139740837	0.424244637	1.143289002
494	0.357070595	2.460393919	3.031665398	3.196165913
495	0.138433279	0.130434926	0.077393665	0.091084364
496	3.580027438	2.321540379	3.043131261	0.180331733
497	0.091119602	0.398051353	0.188937128	0.261108837
498	0.087576163	0.088471764	0.066331746	1.050365622
499	0.439940674	0.129124767	0.977920599	1.024760659
500	0.861099483	0.581630454	1.030752479	0.316496768
501	0.694211602	0.395757615	0.202821016	1.189430317
502	0.286411357	0.090345528	0.349536056	0.144414996
503	0.103493028	0.101804811	0.213345584	0.077130422
504	0.109160785	0.105098727	0.069876379	0.175328288

505	0.347739989	0.105228391	0.067791634	0.515353597
506	0.264258823	0.22983008	0.197745428	0.075142834
507	0.08901721	0.395017178	0.359727437	0.075142834
508	0.087576163	0.088471764	0.066331746	0.291255411
509	0.107197691	0.105228391	0.210755651	0.638105492
510	0.141857352	0.637700543	0.800524722	0.240443215
511	0.135678165	0.129124767	0.076126768	0.094006653
512	0.087576163	0.088471764	0.066331746	0.073706934
513	0.091119602	0.090345528	0.066940746	1.154230172
514	0.091119602	0.420312109	0.203181816	0.851793502
515	0.110893775	0.108439796	0.068807153	0.079538369
516	1.381684493	0.387850666	0.328699586	1.102979404
517	1.07042214	3.255432013	1.959935776	3.195907388
518	0.085082791	0.196815634	0.655334279	0.97919996
519	0.166099874	0.481665472	0.750941085	0.543012771
520	0.258588184	0.089901689	0.246291991	0.075142834
521	0.138433279	0.655781163	0.29780896	0.224358658
522	1.319887412	2.975569843	3.028770577	1.85847903
523	0.371573841	0.110177698	0.90772177	0.514654012
524	0.435262966	0.383190061	0.882829826	1.245528145
525	0.110893775	0.311004408	0.068807153	0.079538369
526	0.135678165	0.129124767	0.076126768	0.094006653
527	0.500033614	0.437985523	0.275917771	0.231743498
528	0.141406262	0.126361819	0.272178415	0.970724999
529	0.166099874	0.325176768	0.084757509	0.103720001
530	0.083956901	0.0864428	0.065779415	0.456149506
531	0.08901721	0.089901689	0.238422874	0.461420833
532	0.315360991	0.559974056	1.559911631	0.344725607
533	0.511266793	0.47055968	0.083627312	0.099481825
534	0.442301018	0.197400692	1.93346237	1.164204615
535	0.158914733	0.146389366	0.083627312	0.099481825
536	0.087576163	0.088471764	0.066331746	0.073706934
537	0.281268677	0.0864428	0.065779415	0.697341862
538	0.331827374	0.293791143	0.229739427	0.076965862
539	0.138433279	0.130434926	0.439774554	0.089500785
540	0.315360991	1.141441399	0.327817383	0.303888163
541	0.26377561	0.224344989	0.066359393	0.075142834
542	0.152455864	0.139740837	0.075453836	0.090952169
543	0.08901721	0.400090753	0.400104151	0.144043804
544	0.091119602	0.090345528	1.289359237	0.137511819
545	0.77492216	0.126361819	0.075049911	0.088980773
546	0.788004227	3.330121142	3.033792243	3.191302975
547	0.110893775	0.108439796	0.068807153	0.275493034
548	0.371426355	0.560288126	0.652930341	0.673513165

549	0.094587279	0.091130385	0.714419376	0.788722609
550	4.219888266	3.329784345	3.045310222	1.099326615
551	0.135678165	0.129124767	0.281773063	0.419676338
552	2.358726168	1.139945929	1.607690029	1.00631444
553	0.27382548	0.398879817	0.193867188	2.425102006
554	0.369913402	0.108439796	0.477859309	0.079538369
555	4.263837638	3.270892559	1.492772491	3.098014976
556	1.128164938	0.63753298	1.134280622	0.676192779
557	0.103493028	0.101804811	0.067420073	0.2515109
558	1.375777605	3.256224402	0.754059619	0.889201245
559	0.141406262	0.383880881	0.075049911	0.97747818
560	0.244841782	0.217887098	0.325907843	0.44690629
561	0.094587279	0.443488634	0.591925237	0.210759918
562	0.756253846	0.742937943	0.9405889	0.297167765
563	0.141406262	0.380939771	0.705787784	0.088980773
564	0.166099874	0.467499671	0.084757509	0.305656716
565	0.152455864	0.139740837	0.608575366	0.090952169
566	0.087576163	0.215451733	0.262893812	0.137704165
567	0.52812172	1.025549315	0.28005548	0.678883615
568	0.094412722	0.090798824	0.704198346	0.077130422
569	0.364762436	0.105228391	0.211513623	0.076965862
570	0.166099874	0.14966126	0.084757509	0.073839518
571	0.087576163	0.088471764	0.066331746	0.073706934
572	0.337904489	0.695826262	0.321472275	0.690780505
573	0.083956901	0.660164057	0.288377517	0.074908944
574	0.256405708	0.090345528	0.510227475	1.096614123
575	0.083956901	0.0864428	0.223097035	0.073839518
576	1.178682316	0.707721478	0.401968174	0.816655797
577	0.091119602	0.225598774	0.366984286	0.191115407
578	0.451626722	0.424813893	0.270173447	0.351122855
579	0.138433279	0.13352282	0.077393665	0.224700833
580	0.278397958	0.585006668	0.360736154	1.225290938
581	0.466787376	0.454063804	0.881462609	2.024923454
582	0.152455864	0.441091806	0.075453836	0.231876395
583	0.094412722	0.401932979	0.066493222	0.144715448
584	0.264258823	0.41712016	0.218399073	0.216801969
585	0.141406262	0.126361819	0.560813633	0.388551688
586	0.339293017	0.40313264	0.604893034	0.075245292
587	0.278450077	0.229788546	0.066493222	0.075245292
588	0.764913557	0.887353763	0.803671193	1.174367152
589	0.107197691	0.507362971	0.067791634	0.077643138
590	0.107197691	0.504460582	0.273349386	3.192495196
591	0.492299718	1.601182542	1.229985949	1.050249905
592	0.152455864	0.71229636	0.275481494	0.706141388

593	1.617407579	1.988530993	1.492378187	2.808659187
594	0.921683041	0.5375562	0.75770276	0.455040039
595	1.991084424	0.737952809	3.048773348	0.434721509
596	1.443350722	3.329043151		1.085883899
597	3.875552661	1.405580478	2.209400176	1.170961706
598	1.032564684	0.534991695	3.050873873	1.142309864
599	4.311711419	3.201857977	3.046975331	0.638441581
601	0.435667298	3.209739709	1.186700578	
602				1.249556571
603	0.141406262	3.328580097	3.04734016	
604	0.783293549	0.38119562	1.002064435	0.618371436
605	3.175406273	0.682450142	0.470387874	0.089500785
607	1.539693387	3.300374003	1.765797956	3.196149259
608	0.578249361	0.941828248	0.748735177	0.222622975
609	2.380414586	1.034123399	0.888930254	2.080337447
610	1.221251851	2.924569691	2.960558322	3.196199239
611	0.286411357	0.787277904	0.836806717	3.196198737
612	0.109464553	0.400913803	1.221809217	0.136603286
615	4.082535852	0.518543459	1.313023688	3.196199216
616	0.647998262	2.390683367	1.106263741	3.195579184
617	0.255374669	0.249888743	1.939424901	0.548162893
618	0.43847318	0.409954324	0.066144604	0.137446681
619	0.818260546	0.480140187	0.293665277	0.25072125
620		0.725366309	0.280029716	0.252640516

(B) Comparative summary of relative rate across four AGO Classes.

Class	ML shape parameter	Number of sites	
		Slow evolving	Fast evolving
Class I	0.5881	405	182
Class II	0.7455	388	199
Class III	1.0174	361	215
Class IV	0.7922	370	215
