# Supplementary material for: Molecular evolution and diversification of the Argonaute family of proteins in plants
Source: BMC Plant Biol. 2015 Jan 28;15:23. doi: 10.1186/s12870-014-0364-6 (PMC4318128; doi:10.1186/s12870-014-0364-6)
Supplement: Additional file 11: Table S4. — Functional diversification in AGOs among four classes. (A) Coefficient of Type I (θI) divergence (i.e functional divergence) and functional distance (dF) between different AGO Classes. The upper diagonal shows the 'θI ± SE' values for all pairwise combinations of AGOs Classes. The lower diagonal shows functional distance (dF) between respective Classes. (B) Site-specific profile of posterior probability (PP) responsible for the Class specific type I functional divergence among AGOs. The colored cells are the corresponding sites between AGO Classes indicating a strong probability of divergence (PP > 0.90). [file 12870_2014_364_MOESM11_ESM.pdf]

Table S4. Functional diversification in AGOs among four classes.

(A) Coefficient of Type I (θ_I) divergence (i.e functional divergence) and functional distance (dF) between different AGO Classes. The upper diagonal shows the 'θ_I ± SE' values for all pairwise combinations of AGOs Classes. The lower diagonal shows functional distance (dF) between respective Classes.

	Class I	Class II	Class III	Class IV
Class I		0.2814 ± 0.0466	0.4265 ± 0.0494	0.6509 ± 0.0432
Class II	0.3304		0.5728 ± 0.0538	0.5816 ± 0.0401
Class III	0.5559	0.8505		0.5784 ± 0.0396
Class IV	1.0525	0.8713	0.8637	

(B) Site-specific profile of posterior probability (PP) responsible for the Class specific type I functional divergence among AGOs. The coloured cells are the corresponding sites between AGO Classes indicating a strong probability of divergence (PP > 0.90).

Site number in dataset II	Class I Vs Class II	Class I Vs Class III	Class I Vs Class IV	Class II Vs Class III	Class II Vs Class IV	Class IIII Vs Class IV
72	0.322824	0.478866	0.913696	0.427683	0.992458	0.996616
73	0.156638	0.302275	0.753962	0.455145	0.809192	0.608963
74	0.238123	0.36259	0.505175	0.389794	0.497922	0.470832
75	0.194752	0.370742	0.505729	0.707709	0.630097	0.450713
76	0.265108	0.471088	0.550868	0.470212	0.905155	0.955127
77	0.322824	0.333408	0.582696	0.56702	0.505832	0.475486
78	0.425363	0.399034	0.612915	0.969322	0.993799	0.440584
79	0.189823	0.565282	0.573857	0.707121	0.504013	0.620782
80	0.448146	0.291782	0.57342	0.758115	0.641175	0.488106
82	0.20059	0.327417	0.517223	0.433752	0.392552	0.405572
83	0.44303	0.406051	0.919793	0.520411	0.409747	0.68343
84	0.189823	0.416559	0.493183	0.56702	0.422082	0.646012
85	0.197776	0.333408	0.519956	0.467822	0.440308	0.440853
86	0.483906	0.451913	0.744395	0.97363	0.997073	0.393028
87	0.920422	0.338331	0.485506	0.792824	0.978264	0.325443
88	0.214187	0.451913	0.518363	0.762977	0.505391	0.58739
89	0.917109	0.136407	0.496239	0.980909	0.895456	0.469466
90	0.300638	0.998246	0.999383	0.60761	0.365311	0.37578
91	0.984348	0.99563	0.995955	0.427683	0.59203	0.592519
92	0.499864	0.405171	0.748629	0.558219	0.505832	0.469968
93	0.300565	0.333406	0.535668	0.558219	0.74003	0.492921
94	0.189823	0.654296	0.575607	0.779787	0.505832	0.720205
95	0.615373	0.477411	0.967719	0.558219	0.422082	0.630764

96	0.444093	0.334254	0.517353	0.815188	0.74	0.46246
97	0.841394	0.610813	0.621835	0.333917	0.799131	0.408659
99	0.018371	0.676261	0.999355	0.58197	0.991652	0.494044
100	0.138889	0.262792	0.418434	0.295384	0.273888	0.263813
102	0.135901	0.916415	0.954205	0.341881	0.211036	0.264596
103	0.189823	0.889868	0.941625	0.939651	0.92693	0.301622
125	0.200973	0.572187	0.51651	0.522386	0.39313	0.638017
126	0.302214	0.451913	0.518147	0.427683	0.588549	0.588417
128	0.449117	0.304447	0.900028	0.685919	0.422082	0.824348
129	0.166306	0.282848	0.759172	0.450012	0.638222	0.896248
130	0.157001	0.677089	0.467213	0.858745	0.407231	0.77929
131	0.189823	0.291782	0.658174	0.427683	0.593767	0.594564
132	0.212833	0.345711	0.771653	0.428055	0.478726	0.538865
133	0.201119	0.378673	0.892538	0.418762	0.66652	0.53376
134	0.420397	0.175567	0.960736	0.845173	0.487499	0.990554
135	0.115211	0.252624	0.80721	0.408891	0.889789	0.634581
136	0.299331	0.333936	0.518578	0.555277	0.586805	0.442552
137	0.310302	0.968118	0.564336	0.633464	0.325896	0.810918
138	0.059467	0.066247	0.97691	0.162717	0.766837	0.924429
139	0.197399	0.335524	0.775589	0.469413	0.689363	0.63442
140	0.376045	0.449176	0.566549	0.944928	0.929634	0.488106
141	0.260489	0.321157	0.965278	0.475582	0.691798	0.834398
142	0.45569	0.408065	0.654582	0.524625	0.503965	0.441996
143	0.218366	0.58447	0.447987	0.248542	0.54921	0.868685
144	0.497032	0.384345	0.858585	0.485889	0.232436	0.501136
145	0.189823	0.291782	0.493183	0.427683	0.422082	0.393028
146	0.318807	0.703317	0.775589	0.312801	0.988026	0.999139
147	0.747588	0.554378	0.57517	0.513466	0.907777	0.605732
148	0.127355	0.341889	0.889652	0.59876	0.963603	0.605732
149	0.264975	0.316394	0.837031	0.481209	0.488947	0.61123
150	0.30304	0.376217	0.705764	0.780912	0.924271	0.390047
151	0.404658	0.336093	0.965545	0.817217	0.284048	0.988341
152	0.606833	0.330943	0.582696	0.743684	0.993694	0.616989
153	0.964074	0.536094	0.974791	0.829711	0.285283	0.651405
154	0.244664	0.470696	0.505165	0.359203	0.515339	0.642981
155	0.155053	0.308771	0.931907	0.459942	0.953603	0.823002
156	0.109599	0.22163	0.362727	0.147218	0.46392	0.595356
157	0.167346	0.275155	0.933213	0.419715	0.868684	0.9316
158	0.463971	0.449723	0.518892	0.969344	0.895058	0.584819
159	0.19714	0.336238	0.519956	0.470075	0.440273	0.442016
160	0.034634	0.099529	0.714619	0.160583	0.54789	0.362598
161	0.445339	0.494932	0.985718	0.208491	0.18768	0.37543
162	0.453803	0.4337	0.328404	0.997343	0.956896	0.238536
163	0.707154	0.478866	0.775589	0.995508	0.999826	0.393028

164	0.075955	0.234674	0.267983	0.512368	0.121178	0.586704
175	0.434109	0.220679	0.744149	0.621741	0.998906	0.942375
176	0.172048	0.320724	0.461457	0.359312	0.37568	0.414515
194	0.930138	0.340368	0.96814	0.829605	0.586805	0.63659
305	0.013627	0.702485	0.033121	0.680602	0.044698	0.985866
306	0.199319	0.396044	0.85607	0.538741	0.818543	0.466127
307	0.344951	0.221059	0.227754	0.32084	0.398429	0.210636
308	0.199249	0.449176	0.680405	0.597891	0.613157	0.931655
309	0.083041	0.375031	0.943743	0.281931	0.478752	0.349312
320	0.296579	0.995071	0.999179	0.866028	0.797291	0.488641
321	0.440494	0.467677	0.463122	0.972345	0.800116	0.644191
322	0.189823	0.291782	0.727195	0.427683	0.670455	0.684061
346	0.068714	0.16103	0.999868	0.170298	0.964363	0.955758
357	0.199108	0.449176	0.741057	0.599628	0.685936	0.393028
358	0.299768	0.664053	0.493183	0.523732	0.685936	0.925573
359	0.299768	0.291782	0.493183	0.599628	0.685936	0.393028
360	0.197776	0.478866	0.583261	0.599628	0.488741	0.490248
361	0.299768	0.291782	0.493183	0.599628	0.685936	0.393028
362	0.123268	0.265838	0.78326	0.453768	0.90434	0.597194
363	0.189823	0.291782	0.573857	0.427683	0.504013	0.488641
364	0.189823	0.291782	0.493183	0.427683	0.422082	0.393028
365	0.302936	0.869076	0.920162	0.687383	0.593135	0.32877
366	0.189823	0.559231	0.493183	0.701899	0.422082	0.843575
367	0.301614	0.87427	0.654181	0.696622	0.440059	0.843243
368	0.189823	0.665486	0.999859	0.788399	0.999837	0.971339
369	0.189823	0.291782	0.493183	0.427683	0.422082	0.393028
370	0.075755	0.170316	0.257643	0.285346	0.162208	0.274667
371	0.199297	0.346657	0.970217	0.485755	0.962987	0.901972
376	0.156923	0.303837	0.436552	0.243991	0.24775	0.249174
378	0.213025	0.317941	0.517641	0.63063	0.660332	0.304957
379	0.299742	0.333347	0.517699	0.558219	0.590725	0.441987
380	0.365361	0.299239	0.786902	0.825893	0.147727	0.896807
381	0.764502	0.691443	0.928789	0.398164	0.310893	0.358316
382	0.097621	0.639786	0.661463	0.654711	0.666966	0.999906
383	0.189823	0.879941	0.964628	0.933756	0.955821	0.271401
384	0.072404	0.360952	0.275127	0.622049	0.146465	0.842182
385	0.299742	0.621792	0.565287	0.94924	0.504467	0.967188
386	0.189823	0.291782	0.493183	0.427683	0.422082	0.393028
387	0.302936	0.688	0.981535	0.53251	0.80992	0.465181
388	0.165889	0.689884	0.758183	0.758373	0.636502	0.490784
389	0.877755	0.416559	0.962331	0.833824	0.271993	0.793179
390	0.189823	0.291782	0.493183	0.427683	0.422082	0.393028
391	0.189823	0.291782	0.493183	0.427683	0.422082	0.393028
392	0.322824	0.478866	0.5844	0.427683	0.504467	0.489177

393	0.189823	0.291782	0.574295	0.427683	0.504467	0.489177
394	0.189823	0.416559	0.493183	0.56702	0.422082	0.646012
395	0.189823	0.291782	0.493183	0.427683	0.422082	0.393028
396	0.189823	0.291782	0.493183	0.427683	0.422082	0.393028
397	0.199051	0.341335	0.565806	0.480268	0.487228	0.604813
398	0.299768	0.899907	0.493183	0.736657	0.685936	0.995391
399	0.201864	0.327362	0.584862	0.433965	0.384744	0.434898
400	0.307743	0.222989	0.390578	0.751105	0.601828	0.348689
402	0.157182	0.806581	0.521476	0.772918	0.502838	0.998002
404	0.607028	0.410083	0.988351	0.612795	0.418262	0.926866
407	0.322824	0.478866	0.582696	0.427683	0.505832	0.490784
408	0.189823	0.291782	0.493183	0.427683	0.422082	0.393028
409	0.189823	0.557211	0.57517	0.700147	0.505377	0.608885
410	0.300153	0.334103	0.741057	0.554856	0.422082	0.624884
411	0.189823	0.291782	0.493183	0.427683	0.422082	0.393028
412	0.189823	0.405648	0.57342	0.555697	0.503558	0.470215
413	0.189823	0.291782	0.57342	0.427683	0.503558	0.488106
414	0.189823	0.410083	0.654582	0.560318	0.589855	0.441639
415	0.189823	0.558019	0.493183	0.700848	0.422082	0.842348
417	0.189823	0.291782	0.998649	0.427683	0.998388	0.999453
418	0.299331	0.448082	0.739714	0.427683	0.422082	0.393028
419	0.189823	0.291782	0.493183	0.427683	0.422082	0.393028
420	0.302936	0.987359	0.921017	0.936076	0.594879	0.638145
421	0.440421	0.92401	0.930874	0.525029	0.395187	0.494887
422	0.758042	0.291782	0.493183	0.937005	0.987852	0.393028
423	0.460529	0.404441	0.963497	0.531425	0.463775	0.818834
424	0.189823	0.291782	0.493183	0.427683	0.422082	0.393028
425	0.446719	0.359028	0.502317	0.933951	0.674765	0.710202
426	0.450256	0.565282	0.493183	0.42748	0.876557	0.849591
427	0.496155	0.451913	0.965067	0.975564	0.234797	0.998777
428	0.322824	0.32912	0.775589	0.698036	0.422082	0.839039
429	0.961004	0.290698	0.996922	0.887055	0.504013	0.888525
430	0.322824	0.327307	0.519956	0.687358	0.588549	0.476568
431	0.189823	0.291782	0.493183	0.427683	0.422082	0.393028
433	0.110981	0.22224	0.797295	0.497677	0.977451	0.598656
434	0.227236	0.409508	0.463395	0.470587	0.643855	0.735968
435	0.197666	0.478866	0.583261	0.600322	0.489159	0.490248
436	0.198608	0.33803	0.822451	0.47459	0.777279	0.645919
437	0.300153	0.449176	0.741057	0.427683	0.422082	0.393028
438	0.322824	0.331781	0.518487	0.710916	0.749724	0.399783
439	0.189823	0.291782	0.654982	0.427683	0.59029	0.590469
440	0.14224	0.324316	0.409551	0.462711	0.331112	0.47051
441	0.322824	0.330869	0.623843	0.706774	0.894658	0.442351
442	0.189823	0.416559	0.573857	0.56702	0.504013	0.477099

503	0.189823	0.291782	0.493183	0.427683	0.422082	0.393028
504	0.189823	0.291782	0.573857	0.427683	0.504013	0.488641
505	0.322824	0.478866	0.584975	0.427683	0.504013	0.488641
506	0.197666	0.478866	0.775589	0.600322	0.686917	0.393028
507	0.300295	0.408065	0.493183	0.469855	0.686917	0.630764
508	0.189823	0.291782	0.57517	0.427683	0.505377	0.490248
509	0.189823	0.291782	0.725804	0.427683	0.66888	0.682246
510	0.441227	0.408065	0.574295	0.5173	0.631718	0.471039
511	0.189823	0.291782	0.493183	0.427683	0.422082	0.393028
512	0.189823	0.291782	0.493183	0.427683	0.422082	0.393028
513	0.189823	0.291782	0.958874	0.427683	0.948584	0.966763
514	0.297142	0.404039	0.92126	0.470686	0.607274	0.691016
515	0.189823	0.291782	0.493183	0.427683	0.422082	0.393028
516	0.448637	0.307354	0.483028	0.676843	0.896565	0.480711
517	0.140785	0.265207	0.383223	0.533145	0.23363	0.46085
518	0.189823	0.539353	0.962153	0.684476	0.952708	0.581693
519	0.189823	0.569707	0.652979	0.710916	0.588113	0.493942
520	0.322824	0.478866	0.775589	0.427683	0.422082	0.393028
521	0.442128	0.291782	0.573857	0.752924	0.633681	0.488641
522	0.5428	0.506702	0.526651	0.163306	0.442802	0.287664
523	0.322824	0.328418	0.505247	0.694145	0.674374	0.422167
524	0.189823	0.546267	0.913934	0.690583	0.892395	0.458819
525	0.189823	0.291782	0.493183	0.427683	0.422082	0.393028
526	0.189823	0.291782	0.493183	0.427683	0.422082	0.393028
527	0.199278	0.449176	0.565607	0.597543	0.48846	0.489177
528	0.189823	0.404039	0.946087	0.554014	0.932523	0.759315
529	0.297666	0.291782	0.493183	0.596847	0.681995	0.393028
530	0.189823	0.291782	0.651775	0.427683	0.586805	0.586362
531	0.189823	0.408065	0.724758	0.558219	0.667697	0.456168
532	0.198113	0.458019	0.584975	0.641949	0.488947	0.903323
533	0.198404	0.478866	0.775589	0.595802	0.68051	0.393028
534	0.19714	0.378175	0.549158	0.543479	0.496875	0.37104
535	0.189823	0.291782	0.493183	0.427683	0.422082	0.393028
536	0.189823	0.291782	0.493183	0.427683	0.422082	0.393028
537	0.322824	0.478866	0.592875	0.427683	0.868273	0.896376
539	0.189823	0.291782	0.493183	0.427683	0.422082	0.393028
540	0.462802	0.326279	0.520488	0.67575	0.924405	0.473203
541	0.197666	0.478866	0.775589	0.600322	0.686917	0.393028
542	0.189823	0.291782	0.493183	0.427683	0.422082	0.393028
543	0.189823	0.408065	0.493183	0.558219	0.422082	0.630764
544	0.189823	0.918848	0.493183	0.956463	0.422082	0.997011
545	0.189823	0.291782	0.493183	0.427683	0.422082	0.393028
546	0.876638	0.54044	0.945973	0.455322	0.172146	0.342172
547	0.189823	0.291782	0.493183	0.427683	0.422082	0.393028

548	0.298191	0.532424	0.724409	0.474564	0.438408	0.423039
549	0.189823	0.677549	0.937885	0.797559	0.922248	0.372296
550	0.02599	0.059866	0.999922	0.105505	0.999988	0.999757
551	0.189823	0.291782	0.653781	0.427683	0.588984	0.588931
552	0.326824	0.627189	0.972101	0.384902	0.426485	0.414051
553	0.437626	0.291782	0.989628	0.748982	0.656818	0.993683
554	0.322824	0.336238	0.775589	0.558219	0.422082	0.630764
555	0.109506	0.993888	0.816515	0.792971	0.204772	0.727371
556	0.16562	0.276631	0.616457	0.433159	0.5008	0.703421
558	0.096253	0.81002	0.796115	0.992868	0.988942	0.497973
559	0.302936	0.291782	0.728928	0.603788	0.436235	0.686322
560	0.189823	0.569707	0.65338	0.710916	0.588549	0.493458
561	0.189823	0.538539	0.493183	0.683753	0.422082	0.821648
562	0.182963	0.217269	0.782864	0.486202	0.502018	0.841336
563	0.299242	0.706668	0.493183	0.548888	0.684953	0.947521
564	0.189823	0.291782	0.732715	0.427683	0.676714	0.691265
565	0.189823	0.687309	0.493183	0.804877	0.422082	0.938234
566	0.189823	0.291782	0.574295	0.427683	0.504467	0.489177
567	0.235139	0.337437	0.50538	0.608364	0.482658	0.462907
568	0.189823	0.812452	0.493183	0.891805	0.422082	0.982
569	0.322824	0.478866	0.775589	0.427683	0.422082	0.393028
570	0.189823	0.291782	0.493183	0.427683	0.422082	0.393028
571	0.189823	0.291782	0.493183	0.427683	0.422082	0.393028
572	0.199623	0.478866	0.54441	0.750827	0.384824	0.834954
573	0.452066	0.291782	0.493183	0.761449	0.877987	0.393028
574	0.322824	0.416851	0.636124	0.8647	0.903444	0.326491
575	0.189823	0.540981	0.493183	0.685919	0.422082	0.824348
576	0.23485	0.695002	0.405711	0.51814	0.417803	0.694571
577	0.189823	0.291782	0.574295	0.427683	0.504467	0.489177
578	0.322824	0.478866	0.5844	0.427683	0.504467	0.489177
579	0.189823	0.291782	0.493183	0.427683	0.422082	0.393028
580	0.19714	0.333408	0.775937	0.467877	0.758211	0.836682
581	0.322824	0.364798	0.588444	0.797559	0.863887	0.360077
582	0.189823	0.291782	0.493183	0.427683	0.422082	0.393028
583	0.189823	0.291782	0.493183	0.427683	0.422082	0.393028
584	0.197666	0.333408	0.582696	0.467826	0.488672	0.475486
585	0.189823	0.679654	0.493183	0.799144	0.422082	0.934255
586	0.322824	0.371227	0.775589	0.808461	0.422082	0.940649
587	0.322824	0.478866	0.775589	0.427683	0.422082	0.393028
588	0.322824	0.418041	0.720409	0.865826	0.94719	0.323049
589	0.189823	0.291782	0.493183	0.427683	0.422082	0.393028
590	0.189823	0.291782	0.982047	0.427683	0.977729	0.987773
591	0.19714	0.689162	0.622135	0.83075	0.582484	0.437617
592	0.189823	0.410083	0.723009	0.560318	0.66572	0.453989

593	0.209469	0.181774	0.425498	0.369139	0.329213	0.273598
594	0.699853	0.543461	0.901224	0.558639	0.504922	0.470935
595	0.344712	0.621087	0.926761	0.996123	0.489159	0.999996
604	0.189823	0.805378	0.784742	0.887216	0.736631	0.493012
605	0.499864	0.297107	0.928308	0.710916	0.422082	0.853881
607	0.04825	0.956314	0.125433	0.869028	0.130346	0.97557
608	0.201789	0.309482	0.759075	0.636671	0.969263	0.588766
610	0.173157	0.143875	0.35226	0.630174	0.82647	0.111261
611	0.443844	0.332897	0.918816	0.908688	0.998739	0.545036
612	0.322824	0.554213	0.775589	0.943304	0.422082	0.995016
615	0.301614	0.700631	0.999958	0.541724	0.998799	0.984234
616	0.228554	0.511726	0.193878	0.393142	0.729198	0.889264
617	0.322824	0.592025	0.518702	0.95479	0.750384	0.648999
618	0.189823	0.291782	0.493183	0.427683	0.422082	0.393028
619	0.189823	0.291782	0.493183	0.427683	0.422082	0.393028
620	0.19714	0.333408	0.775589	0.467877	0.691798	0.646012
