# Supplementary material for: Molecular evolution and diversification of the Argonaute family of proteins in plants
Source: BMC Plant Biol. 2015 Jan 28;15:23. doi: 10.1186/s12870-014-0364-6 (PMC4318128; doi:10.1186/s12870-014-0364-6)
Supplement: Additional file 14: Table S5. — Correlated amino acid pairs among different Classes. (A) Column ‘AA1’ and ‘AA2’ are the positions in the alignment of AGO sequences of respective Classes between which correlation coefficient (in the last column) were calculated by CAPS 2.0. (B) Summary of coevolving amino acid pairs in NaAGO1a, NaAGO5, NaAGO2 and NaAGO4a, representatives of Class I – IV, respectively. (C) Frequency of coevolving amino acid (AA) pairs in NaAGO1a, NaAGO5, NaAGO2 and NaAGO4a. [file 12870_2014_364_MOESM14_ESM.pdf]

Table S5. Correlated amino acid pairs among different Classes.

(A) Column ‘AA1’ and ‘AA2’ are the positions in the alignment of AGO sequences of respective Classes between which correlation coefficient (in the last column) were calculated by CAPS 2.0.

Class I

AA1	AA2	Correlation coefficient	AA1	AA2	Correlation coefficient	AA1	AA2	Correlation coefficient
20	21	0.57924	423	645	0.609695	1013	1016	0.716607
20	54	0.603423	423	659	0.634627	1020	1034	0.630224
20	64	0.619498	423	712	0.578224	1020	1522	0.641321
20	274	0.523343	423	754	0.568959	1020	1619	0.568276
20	672	0.564881	423	763	0.740555	1025	1270	0.579325
21	54	0.816396	423	778	0.745258	1025	1496	0.602411
21	64	0.701888	423	788	0.500501	1027	1064	0.711181
21	550	0.707539	423	813	0.563803	1027	1066	0.642624
21	641	0.548905	423	815	0.593309	1030	1223	0.679952
21	672	0.673135	423	816	0.552393	1030	1294	0.613071
23	48	0.744128	423	1878	0.656213	1030	1311	0.906067
23	278	0.6051	423	1879	0.582037	1030	1314	0.691258
47	271	0.701646	423	1884	0.523883	1030	1321	0.75318
47	1438	0.548403	423	1885	0.696848	1030	1517	0.682404
48	278	0.624165	423	1887	0.591387	1034	1068	0.622225
51	391	0.658508	424	442	0.639096	1034	1522	0.628866
54	550	0.540519	427	437	0.545319	1034	1833	0.668949
54	672	0.532169	427	443	0.608416	1034	1915	0.652649
62	253	0.706336	427	643	0.572147	1035	1374	0.894675
64	274	0.560797	427	703	0.548363	1035	1379	0.618917
64	550	0.55225	427	734	0.567629	1036	1265	0.757935
64	641	0.589351	427	776	0.549956	1041	1042	0.833815
64	672	0.597033	427	1470	0.579241	1041	1517	0.551419
66	712	0.628189	435	437	0.557171	1042	1276	0.552614
66	788	0.631435	435	445	0.660948	1042	1316	0.602338
67	215	0.614825	435	644	0.637158	1063	1276	0.584554
67	439	0.661461	435	686	0.575278	1063	1706	0.609233
72	749	0.553941	437	445	0.614755	1063	1710	0.634266
74	175	0.53767	437	604	0.533204	1063	1923	0.721534
96	97	0.5549	437	644	0.636704	1064	1077	0.561459
96	98	0.726821	437	645	0.630853	1064	1433	0.657249
96	157	0.739885	437	659	0.55229	1064	1502	0.654237
96	158	0.514148	437	703	0.556429	1064	1706	0.641012
96	196	0.630231	437	754	0.544274	1064	1751	0.650958
96	197	0.580006	437	763	0.732225	1066	1256	0.654908
96	203	0.673606	437	778	0.661607	1066	1706	0.648717

96	214	0.523162	437	788	0.487677	1066	1710	0.708668
96	256	0.568595	437	813	0.594968	1066	1921	0.784862
96	257	0.626314	437	815	0.565677	1067	1068	0.636912
96	277	0.596919	437	816	0.510862	1067	1637	0.620292
96	279	0.653452	437	834	0.558261	1068	1156	0.677563
96	282	0.608331	437	1878	0.628536	1068	1577	0.536387
96	283	0.743704	437	1879	0.562533	1068	1833	0.758096
96	423	0.83912	437	1884	0.560425	1068	1873	0.695522
96	427	0.549491	437	1885	0.609967	1074	1507	0.717803
96	435	0.550099	437	1887	0.578577	1076	1507	0.95027
96	437	0.822463	437	1907	0.601698	1076	1706	0.769221
96	445	0.586883	438	442	0.638035	1076	1710	0.824349
96	604	0.552185	445	604	0.532898	1077	1703	0.645603
96	644	0.693229	445	644	0.652105	1077	1706	0.60141
96	645	0.623869	445	645	0.566663	1077	1710	0.699113
96	659	0.54387	445	686	0.543327	1077	1807	0.640194
96	703	0.578733	445	763	0.56239	1080	1620	0.763414
96	712	0.519268	445	778	0.55368	1083	1084	0.694753
96	713	0.49192	456	776	0.522023	1083	1091	0.666959
96	754	0.626228	550	641	0.543929	1084	1091	0.871783
96	763	0.723361	604	643	0.584336	1103	1477	0.714154
96	778	0.684254	604	644	0.651518	1155	1667	0.636252
96	803	0.520864	604	1885	0.543596	1155	1784	0.701889
96	813	0.632955	604	1887	0.52123	1156	1873	0.730701
96	815	0.557235	641	686	0.611528	1159	1776	0.96365
96	816	0.613798	641	717	0.598787	1162	1170	0.693164
96	1470	0.584202	643	644	0.652473	1177	1454	0.628142
96	1877	0.593924	644	645	0.592531	1180	1254	0.722256
96	1878	0.712235	644	686	0.607025	1187	1327	0.737747
96	1879	0.592376	644	754	0.543692	1201	1232	0.997732
96	1884	0.588836	644	778	0.535551	1201	1386	0.641918
96	1885	0.688649	645	712	0.48968	1201	1743	0.995005
96	1887	0.655308	645	763	0.599137	1206	1413	0.629867
96	1907	0.601098	645	767	0.529701	1207	1612	0.85844
97	196	0.607453	645	778	0.551802	1214	1294	0.605798
97	197	0.520248	645	815	0.544264	1223	1294	0.6335
97	238	0.563791	645	833	0.561034	1223	1300	0.671964
97	437	0.523148	645	1885	0.606949	1223	1311	0.7999
97	554	0.546665	645	1887	0.657782	1223	1314	0.875754
97	644	0.588742	656	737	0.566048	1223	1321	0.662116
97	767	0.54255	656	741	0.684666	1223	1324	0.664511
97	1512	0.557555	659	712	0.557344	1223	1575	0.75246
98	157	0.578354	659	760	0.537609	1227	1276	0.62125
98	196	0.595266	659	763	0.691681	1227	1815	0.666965

98	283	0.648415
98	423	0.696667
98	427	0.537592
98	437	0.676966
98	644	0.534592
98	645	0.557171
98	659	0.543972
98	713	0.521892
98	734	0.53349
98	763	0.656997
98	778	0.623335
98	815	0.535288
98	1470	0.529476
100	1056	0.557121
157	277	0.539976
157	283	0.66019
157	423	0.615833
157	435	0.525647
157	437	0.615344
157	445	0.555704
157	643	0.518512
157	644	0.527589
157	645	0.541903
157	763	0.631565
157	813	0.528884
157	1878	0.536832
157	1885	0.534608
157	1907	0.557552
158	282	0.556547
172	193	0.604554
172	713	0.586607
172	738	0.545126
172	740	0.540689
172	788	0.577303
193	196	0.557156
193	235	0.588919
193	273	0.547856
193	643	0.504884
193	712	0.612282
193	713	0.664466
193	737	0.555571
193	738	0.585502
193	740	0.580765
193	788	0.698436
659	1879	0.557524
659	1907	0.575433
698	713	0.627563
698	788	0.698213
702	776	0.567168
703	763	0.549698
707	1047	0.63225
708	737	0.638252
708	738	0.581616
708	740	0.57511
708	741	0.596015
711	737	0.526713
712	713	0.640675
712	738	0.568001
712	740	0.563811
712	763	0.507732
712	778	0.510321
712	782	0.528778
712	788	0.669581
713	737	0.621496
713	738	0.651142
713	740	0.645842
713	788	0.725869
713	1907	0.517443
737	738	0.851149
737	740	0.863649
737	741	0.816496
737	742	0.56768
737	788	0.622718
738	740	0.757864
738	741	0.718965
738	742	0.5004
738	788	0.651194
740	741	0.710296
740	788	0.645832
740	1892	0.617868
741	742	0.509446
741	788	0.517039
749	754	0.624661
752	754	0.558814
753	754	0.571677
754	778	0.557641
754	803	0.529645
754	816	0.66206
1232	1386	0.653637
1232	1743	0.995742
1249	1784	0.640525
1256	1921	0.804839
1257	1280	0.60499
1257	1394	0.783833
1257	1433	0.586306
1257	1453	0.646271
1257	1549	0.658895
1257	1554	0.593522
1257	1562	0.783833
1257	1819	0.664625
1257	1864	0.792206
1261	1264	0.71874
1261	1387	0.540296
1261	1426	0.572524
1261	1537	0.677673
1261	1552	0.700802
1261	1722	0.649192
1261	1751	0.860657
1261	1771	0.643353
1261	1819	0.808392
1261	1917	0.846623
1262	1537	0.863296
1262	1751	0.698833
1264	1741	0.652219
1280	1554	0.553639
1280	1864	0.604616
1282	1284	0.729738
1284	1575	0.84615
1294	1300	0.839978
1294	1301	0.689116
1294	1311	0.70083
1294	1314	0.645956
1300	1311	0.71712
1300	1314	0.722972
1302	1321	0.565861
1310	1328	0.597733
1310	1833	0.657931
1311	1314	0.794689
1311	1315	0.621682
1311	1319	0.617629
1311	1321	0.759117
1311	1324	0.757844

193	1907	0.587395	754	1887	0.525626	1311	1575	0.820055
196	197	0.717721	763	767	0.539821	1314	1321	0.694689
196	203	0.60148	763	778	0.737134	1314	1324	0.654468
196	256	0.526755	763	803	0.583847	1314	1575	0.643369
196	257	0.592945	763	813	0.611496	1315	1319	0.735484
196	282	0.531365	763	815	0.664194	1315	1710	0.592335
196	283	0.613774	763	1878	0.655362	1321	1324	0.778491
196	423	0.532448	763	1879	0.704133	1336	1525	0.764748
196	435	0.596508	763	1880	0.521891	1356	1383	0.798611
196	437	0.621543	763	1885	0.601553	1362	1363	0.822489
196	644	0.550636	763	1887	0.573128	1362	1366	0.934703
196	712	0.582281	763	1907	0.656313	1363	1366	0.788041
196	713	0.766575	766	815	0.538564	1374	1379	0.627422
196	763	0.571427	767	777	0.533907	1379	1554	0.648447
196	767	0.570335	767	1907	0.540486	1380	1389	0.709701
196	788	0.554701	775	1056	0.633906	1381	1382	0.759056
196	813	0.570749	775	1512	0.592652	1386	1461	0.628783
196	1907	0.572636	775	1784	0.573721	1386	1743	0.65767
197	203	0.54236	778	815	0.613605	1387	1552	0.64659
197	283	0.507896	778	1878	0.541918	1389	1917	0.70494
197	423	0.564628	778	1885	0.640632	1394	1453	0.721188
197	437	0.542848	781	782	0.537	1394	1496	0.707781
197	645	0.529016	788	1471	0.540143	1394	1549	0.737009
197	712	0.574299	788	1907	0.507196	1394	1552	0.813938
197	763	0.535805	803	804	0.545992	1394	1554	0.824752
197	767	0.542917	803	812	0.575697	1394	1562	1
197	778	0.585176	803	1879	0.539657	1394	1819	0.891004
197	782	0.513989	813	816	0.573938	1394	1864	0.842842
197	1887	0.517388	833	1885	0.532751	1396	1415	0.909312
203	256	0.546717	833	1887	0.641201	1397	1403	0.635296
203	257	0.562607	860	1041	0.583554	1399	1403	0.74204
203	277	0.56478	860	1517	0.59388	1412	1426	0.594049
203	282	0.576571	863	989	0.57586	1413	1741	0.66952
203	283	0.65731	865	1187	0.651334	1413	1751	0.706397
203	423	0.613237	869	886	0.672621	1426	1929	0.550311
203	437	0.606155	869	888	0.869348	1433	1620	0.673631
203	442	0.553811	869	890	0.617772	1433	1864	0.665813
203	644	0.617366	869	892	0.543957	1453	1554	0.710058
203	645	0.546911	869	903	0.574122	1453	1562	0.721188
203	703	0.53199	869	1921	0.717449	1453	1819	0.705351
203	754	0.630611	874	885	0.621757	1453	1864	0.741326
203	763	0.584729	874	888	0.640604	1454	1459	0.731786
203	778	0.612065	874	950	0.644212	1456	1766	0.60241
203	782	0.56891	881	888	0.60402	1456	1807	0.556649

203	815	0.518215	881	893	0.821913	1460	1461	0.575129
203	1470	0.543427	884	899	0.62194	1468	1616	0.627071
203	1887	0.593674	884	903	0.719923	1470	1878	0.539758
212	213	0.568166	884	908	0.69852	1470	1885	0.563708
212	236	0.539072	885	905	0.527564	1470	1887	0.55908
212	552	0.606756	885	916	0.758457	1475	1864	0.741575
214	659	0.560019	886	888	0.750003	1496	1554	0.671069
214	763	0.542184	886	899	0.589557	1496	1562	0.707781
214	1907	0.546011	886	903	0.676367	1496	1819	0.679077
230	245	0.64544	886	905	0.586542	1496	1923	0.592655
231	278	0.575766	886	938	0.600274	1502	1512	0.577481
233	234	0.91531	886	1256	0.636376	1502	1525	0.73221
236	238	0.596424	888	890	0.629425	1507	1706	0.715039
236	797	0.585065	888	899	0.579273	1507	1710	0.790953
238	1880	0.552599	888	903	0.643961	1512	1525	0.601372
238	1887	0.553371	888	912	0.477942	1513	1517	0.57679
253	592	0.660312	888	934	0.738374	1515	1784	0.715902
254	1605	0.672287	888	1066	0.728848	1517	1710	0.792135
255	257	0.568841	888	1256	0.63511	1522	1619	0.591834
255	729	0.565359	888	1921	0.847093	1525	1784	0.688703
256	283	0.531004	892	903	0.60117	1537	1751	0.857563
256	423	0.549315	898	937	0.6125	1537	1771	0.656283
256	437	0.570431	899	903	0.692018	1549	1554	0.561752
256	767	0.643573	899	905	0.667247	1549	1562	0.737009
256	1885	0.524651	899	908	0.685714	1549	1819	0.627083
257	283	0.581846	899	924	0.678765	1550	1572	0.652587
257	423	0.56551	899	937	0.625494	1550	1833	0.654248
257	437	0.612912	903	905	0.600647	1552	1554	0.670872
257	590	0.597857	903	924	0.64049	1552	1562	0.813938
257	644	0.527443	903	926	0.685188	1552	1744	0.647967
257	763	0.57343	903	935	0.597047	1552	1819	0.911378
257	766	0.551112	904	937	0.71932	1552	1864	0.683441
257	777	0.609083	908	923	0.571831	1552	1917	0.667109
257	778	0.533477	908	1168	0.642223	1554	1562	0.824752
257	782	0.506415	911	971	0.612814	1554	1766	0.603773
257	1907	0.551266	912	924	0.746089	1554	1809	0.801538
274	756	0.503974	912	926	0.614657	1554	1819	0.911971
277	279	0.615141	912	934	0.499905	1554	1864	0.767286
277	442	0.626246	916	924	0.761087	1562	1819	0.891004
277	763	0.548592	916	926	0.763773	1562	1864	0.842842
278	291	0.590711	923	937	0.589839	1577	1622	0.612677
278	671	0.527961	923	950	0.670766	1626	1865	0.743719
278	707	0.551194	924	926	0.915191	1637	1915	0.610981
279	283	0.629379	924	937	0.746832	1642	1706	0.590416

279	423	0.667724	926	937	0.670746	1642	1710	0.684962
279	437	0.559602	929	1066	0.613782	1642	1923	0.730469
279	644	0.530012	934	938	0.598691	1659	1662	0.633405
279	1878	0.536828	934	1066	0.680167	1659	1663	0.685544
282	283	0.782548	934	1921	0.688701	1659	1676	0.721652
282	285	0.535601	938	1066	0.586692	1662	1663	0.864419
282	423	0.547696	938	1076	0.627447	1662	1668	0.811429
282	437	0.561898	938	1507	0.630726	1662	1669	0.694677
282	445	0.538727	949	1020	0.897214	1662	1670	0.597775
282	644	0.518716	949	1034	0.651619	1662	1676	0.760542
282	763	0.554269	951	1012	0.571085	1663	1668	0.793604
283	285	0.574649	951	1030	0.763311	1663	1670	0.614167
283	423	0.702306	951	1517	0.582113	1663	1676	0.81427
283	435	0.592025	957	1270	0.554049	1667	1746	0.581202
283	437	0.706492	959	967	0.843689	1668	1669	0.68121
283	445	0.681198	964	967	0.654754	1668	1670	0.647076
283	604	0.54927	964	971	0.68042	1668	1676	0.723783
283	644	0.660914	967	969	0.810715	1670	1673	0.685664
283	645	0.591485	967	999	0.755745	1706	1710	0.921776
283	703	0.533004	967	1001	0.763724	1706	1751	0.758952
283	754	0.56454	967	1006	0.714827	1710	1923	0.63383
283	763	0.710709	969	999	0.723529	1710	1929	0.658637
283	778	0.662857	969	1001	0.748973	1740	1766	0.745614
283	813	0.5718	969	1006	0.65277	1741	1751	0.724656
283	815	0.619189	978	997	0.599408	1744	1819	0.60009
283	1878	0.596708	982	1669	0.696475	1751	1771	0.752786
283	1885	0.527401	997	1001	0.632401	1766	1807	0.625742
283	1887	0.547493	999	1001	0.83189	1809	1864	0.723159
290	394	0.594615	999	1006	0.905137	1819	1864	0.79185
290	737	0.757315	999	1014	0.67608	1819	1917	0.898944
290	738	0.739341	1001	1006	0.699954	1877	1878	0.63376
290	740	0.640533	1001	1014	0.637661	1877	1879	0.54158
290	741	0.58946	1002	1012	0.633733	1877	1884	0.510519
294	1438	0.630368	1006	1010	0.545162	1878	1879	0.614124
391	456	0.581388	1006	1012	0.589139	1878	1885	0.670313
392	393	0.535418	1006	1013	0.512689	1878	1887	0.630339
407	552	0.533239	1006	1014	0.668552	1879	1885	0.608909
423	427	0.628012	1006	1016	0.691386	1879	1887	0.581224
423	437	0.801597	1010	1014	0.588946	1879	1907	0.631416
423	445	0.593371	1012	1013	0.593611	1885	1887	0.83025
423	604	0.542445	1012	1016	0.684281			
423	644	0.63331	1012	1279	0.609275			

Class II								
AA1	AA2	Correlation coefficient	AA1	AA2	Correlation coefficient	AA1	AA2	Correlation coefficient
4	235	0.665342	549	1384	0.67553	748	1097	0.769635
61	414	0.586699	550	1012	0.740204	748	1105	0.815254
61	1134	0.778269	560	787	0.630522	748	1232	0.732431
150	159	0.660291	563	1062	0.659572	748	1237	0.690611
157	1391	0.732726	568	752	0.711085	748	1384	0.632657
159	312	0.683762	568	834	0.613652	752	925	0.654437
159	1095	0.702915	568	925	0.660648	752	1152	0.844112
160	317	0.671162	568	1152	0.856627	752	1207	0.68169
160	489	0.680703	568	1207	0.654408	753	976	0.573098
160	522	0.695574	570	609	0.791579	753	1392	0.582193
160	1084	0.659581	570	616	0.761455	754	1247	0.729702
160	1237	0.668407	570	631	0.795092	755	1084	0.712258
162	848	0.620866	570	1368	0.762504	757	1045	0.812448
166	734	0.65208	570	1376	0.739962	757	1691	0.728523
166	1045	0.674536	572	1113	0.816619	758	947	0.717611
193	958	0.69571	573	1388	0.669632	758	1387	0.923513
198	295	0.758013	574	909	0.795498	759	852	0.694054
251	573	0.680909	575	920	0.838911	760	950	0.82669
251	594	0.641866	575	1388	0.744654	760	1012	0.890558
268	299	0.763054	577	580	0.715803	760	1714	0.84294
315	405	0.629931	582	609	0.754006	768	955	0.738909
315	407	0.634177	582	616	0.721229	789	1320	0.656055
319	323	0.805671	582	1098	0.680412	790	896	0.693774
334	717	0.703946	582	1108	0.670359	790	1162	0.689205
365	474	0.733214	582	1368	0.723295	790	1314	0.732504
367	1270	0.783631	583	589	0.688911	793	847	0.712371
375	614	0.619404	586	1105	0.684961	801	990	0.999306
383	556	0.611644	587	743	0.746374	811	1204	0.657591
386	403	0.692734	589	913	0.665924	817	847	0.691694
386	1198	0.740865	589	916	0.75741	818	833	0.921877
414	440	0.714476	594	844	0.679483	818	1161	0.918863
448	449	0.615254	594	959	0.682644	818	1266	0.893004
448	469	0.81322	595	596	0.761752	818	1267	0.896706
448	867	0.679347	595	609	0.790063	818	1321	0.906917
449	468	0.707268	595	616	0.755448	822	824	0.740736
451	1062	0.746535	595	1368	0.757825	822	1219	0.771508
451	1710	0.802338	596	609	0.872821	826	966	0.774281
452	564	0.621947	596	616	0.835694	833	1161	0.999236
461	462	0.649545	596	661	0.829361	833	1266	0.98109
462	1219	0.694378	596	720	0.628246	833	1321	0.991911
464	827	0.730133	596	1368	0.837448	834	925	0.690716

464	1301	0.644218
465	516	0.771009
465	522	0.735333
465	596	0.804092
465	609	0.812734
465	616	0.815304
465	755	0.777039
465	1368	0.769827
466	914	0.651485
469	615	0.821116
469	867	0.859437
474	948	0.687766
477	528	0.897338
477	625	0.891378
481	1368	0.741607
483	1100	0.743766
483	1647	0.644949
485	1111	0.821834
489	522	0.894329
489	550	0.822676
489	610	0.690372
489	616	0.746762
489	1012	0.821125
491	570	0.621289
491	654	0.67674
493	565	0.686579
493	833	0.849456
493	1161	0.839868
493	1266	0.789124
493	1321	0.813552
494	631	0.787287
494	650	0.728399
494	853	0.701932
497	1709	0.654885
498	1035	0.738863
499	1389	0.702236
500	541	0.629062
500	547	0.736737
502	1219	0.779666
502	1260	0.68777
503	630	0.684398
503	1098	0.84603
503	1108	0.793012
504	912	0.699393
601	952	0.764667
606	734	0.667405
606	766	0.688224
606	925	0.680779
606	1207	0.734247
606	1237	0.778761
609	616	0.960385
609	631	0.718164
609	1118	0.773909
609	1368	0.925097
609	1552	0.758709
613	817	0.766435
615	867	0.731103
615	1045	0.71299
615	1085	0.739691
616	1118	0.777038
616	1368	0.893829
616	1552	0.710666
624	911	0.630567
625	1045	0.731863
625	1384	0.615507
625	1552	0.597416
630	1098	0.636483
631	650	0.688944
647	1005	0.706801
648	681	0.66783
648	705	0.844424
648	712	0.698814
648	718	0.699397
648	1075	0.728978
648	1128	0.68047
650	656	0.751371
650	733	0.762943
652	733	0.7554
652	848	0.675593
654	733	0.645482
654	760	0.843205
654	950	0.716758
654	1012	0.756937
654	1714	0.758748
658	1045	0.80216
658	1222	0.794721
660	669	0.711541
660	734	0.874027
847	1105	0.746761
848	966	0.807745
848	1204	0.676385
853	1076	0.634308
856	1326	0.756508
857	1008	0.881416
857	1034	0.771472
857	1213	0.652529
866	1011	0.663242
896	1105	0.667257
913	916	0.666596
913	1376	0.73571
916	1106	0.668198
916	1112	0.699087
917	1101	0.808076
920	1388	0.786801
923	1382	0.681611
947	1085	0.73259
947	1132	0.735003
947	1154	0.777251
947	1387	0.832374
950	1012	0.979642
950	1714	0.872169
951	1035	0.775934
951	1303	0.963294
955	1019	0.694215
957	1714	0.66642
961	1551	0.671326
964	1166	0.608751
966	1311	0.809427
967	1194	0.636441
984	1188	0.796511
994	1075	0.723366
995	1232	0.723139
997	1341	0.622072
1008	1188	0.733731
1012	1714	0.882623
1014	1207	0.672189
1014	1250	0.621378
1014	1251	0.727682
1020	1021	0.64961
1035	1303	0.673562
1042	1314	0.849625
1045	1133	0.781828

508	1188	0.717098
511	859	0.730591
513	1280	0.667687
516	595	0.751232
516	596	0.865125
516	609	0.790398
516	616	0.757082
516	1008	0.782071
516	1368	0.758432
516	1552	0.610469
517	528	0.663847
517	625	0.748132
517	733	0.686928
517	925	0.753748
518	736	0.726943
522	550	0.745768
522	609	0.818234
522	610	0.690459
522	616	0.932147
522	925	0.715467
522	1012	0.738781
522	1118	0.620902
528	625	0.896174
528	660	0.638763
528	1045	0.771811
529	1078	0.711418
530	570	0.951251
530	596	0.655024
530	609	0.838417
530	616	0.802969
530	916	0.761885
530	1368	0.804485
530	1376	0.78469
530	1552	0.699628
539	610	0.678866
539	1098	0.850073
539	1108	0.821226
540	806	0.786273
540	1205	0.712717
541	544	0.74533
544	912	0.677042
544	1388	0.77331
549	606	0.650667
549	1045	0.676871
660	746	0.587487
661	720	0.638882
681	718	0.758174
681	984	0.794063
681	1188	0.72732
681	1384	0.703933
685	976	0.711235
686	760	0.814556
686	950	0.9966
686	1012	0.973583
686	1714	0.862525
689	1150	0.592242
689	1273	0.676046
691	1046	0.754585
692	1132	0.732468
705	712	0.711068
705	1044	0.63777
705	1075	0.776534
711	1014	0.680288
712	1046	0.702793
712	1075	0.781148
712	1128	0.751877
713	758	0.707105
713	947	0.878551
713	1132	0.756157
713	1387	0.784915
722	857	0.73132
722	1008	0.858924
734	766	0.700961
734	1045	0.626961
735	852	0.701421
735	1154	0.684213
736	750	0.676284
738	741	0.767091
738	848	0.813655
739	1148	0.881701
740	972	0.684294
741	742	0.761687
741	848	0.782578
741	966	0.877604
741	1311	0.862973
742	966	0.784293
744	1014	0.847248
746	1045	0.825106
1045	1222	0.788637
1045	1237	0.716753
1045	1384	0.697881
1046	1075	0.723902
1046	1162	0.763694
1052	1054	0.890799
1052	1064	0.857411
1054	1064	0.841389
1062	1063	0.91529
1062	1085	0.652687
1067	1160	0.95754
1068	1160	0.725311
1075	1128	0.80769
1075	1384	0.697904
1077	1154	0.665485
1085	1132	0.770617
1085	1387	0.679134
1095	1360	0.737477
1095	1376	0.901661
1095	1717	0.870654
1097	1105	0.772687
1097	1162	0.778737
1097	1233	0.707286
1098	1108	0.961232
1105	1233	0.704215
1105	1237	0.683615
1149	1267	0.707878
1157	1166	0.796567
1159	1392	0.617786
1161	1227	0.790797
1161	1266	0.987904
1161	1321	0.996114
1162	1314	0.789309
1198	1301	0.683084
1227	1229	0.972974
1237	1342	0.566545
1237	1384	0.779986
1247	1261	0.735938
1266	1321	0.997722
1360	1384	0.782457
1368	1552	0.774546
1376	1717	0.815045
1710	1712	0.765125
1710	1717	0.764378

549	1237	0.65684	748	896	0.729879	1712	1717	0.883776

Class III

AA1	AA2	Correlation coefficient	AA1	AA2	Correlation coefficient	AA1	AA2	Correlation coefficient
8	687	0.725	825	1176	0.708857	1057	1664	0.861407
8	974	0.670083	825	1208	0.649771	1068	1452	0.719719
13	89	0.744844	825	1375	0.78696	1071	1094	0.739824
13	817	0.794157	827	1023	0.856423	1082	1343	0.671674
13	958	0.842772	827	1322	0.811622	1082	1386	0.739431
13	983	0.814527	828	1375	0.647302	1082	1513	0.737121
13	1013	0.913108	829	897	0.741509	1085	1287	0.696701
13	1246	0.808319	829	1346	0.747463	1088	1360	0.69267
13	1331	0.965308	829	1380	0.650546	1090	1496	0.706676
13	1367	0.871233	830	921	0.749197	1093	1227	0.978623
13	1371	0.729235	831	841	0.653686	1094	1180	0.65596
13	1455	0.842772	831	1024	0.680877	1094	1485	0.830409
13	1499	0.732145	831	1045	0.715233	1094	1543	0.772248
13	1558	0.891434	831	1095	0.668268	1095	1135	0.73539
14	43	0.73771	831	1135	0.676891	1095	1496	0.72817
14	112	0.775003	831	1225	0.663197	1098	1154	0.771066
14	117	0.657168	831	1496	0.81408	1098	1223	0.781126
14	122	0.687212	831	1661	0.650319	1098	1246	0.673557
14	206	0.675933	832	897	0.698408	1098	1259	0.720283
14	668	0.687395	832	1496	0.698346	1098	1304	0.949272
24	1260	0.867522	832	1576	0.636421	1098	1364	0.916462
25	76	0.664961	840	1419	0.727338	1098	1382	0.801474
25	774	0.718593	841	1135	0.665511	1098	1455	0.72562
25	846	0.628816	841	1395	0.653231	1098	1499	0.968127
25	1246	0.656301	841	1496	0.650244	1098	1524	0.801238
25	1364	0.699346	843	879	0.721568	1098	1546	0.974645
25	1371	0.69855	843	927	0.737093	1098	1557	0.997947
25	1397	0.757709	843	946	0.732906	1098	1558	0.75834
25	1432	0.649422	843	1503	0.754536	1098	1664	0.845064
26	967	0.644303	845	892	0.683832	1109	1502	0.771883
43	112	0.666328	845	974	0.713045	1109	1534	0.766823
48	76	0.715963	845	1046	0.731305	1114	1384	0.692286
68	323	0.785742	845	1169	0.740632	1114	1451	0.729294
68	418	0.670457	846	861	0.702309	1115	1176	0.754773
68	567	0.724924	846	877	0.705566	1123	1244	0.645863
72	1018	0.696434	846	888	0.825869	1123	1261	0.722184
76	1371	0.675162	846	889	0.793594	1123	1371	0.720865
77	922	0.70993	846	892	0.756167	1125	1126	0.746383
77	1055	0.697072	846	983	0.841632	1135	1330	0.67962
77	1135	0.663414	846	1013	0.702156	1135	1382	0.695669
77	1315	0.698165	846	1057	0.846968	1135	1384	0.687339
77	1323	0.670309	846	1098	0.81002	1135	1397	0.688893
77	1432	0.66487	846	1154	0.833042	1135	1400	0.722473
83	208	0.724705	846	1304	0.839945	1135	1451	0.695911
89	582	0.650497	846	1315	0.805183	1138	1164	0.962084
89	815	0.726023	846	1324	0.727287	1142	1323	0.755986
89	1013	0.678648	846	1362	0.793594	1150	1424	0.733308

89	1246	0.72061	846	1364	0.953304	1154	1201	0.689842
89	1331	0.741199	846	1367	0.826116	1154	1223	0.749513
89	1432	0.642751	846	1371	0.707328	1154	1259	0.732848
108	1031	0.742807	846	1499	0.791159	1154	1304	0.813952
109	967	0.683425	846	1502	0.715189	1154	1324	0.626259
112	206	0.683854	846	1524	0.79115	1154	1346	0.713718
112	668	0.649591	846	1546	0.791373	1154	1364	0.81211
114	207	0.820689	846	1557	0.79271	1154	1382	0.783991
117	122	0.917724	846	1558	0.665063	1154	1384	0.744528
125	154	0.65844	846	1644	0.682001	1154	1451	0.753073
134	1090	0.745703	846	1658	0.738057	1154	1499	0.696816
147	956	0.692777	846	1661	0.687208	1154	1524	0.725108
154	217	0.709242	846	1664	0.942034	1154	1546	0.704687
154	222	0.730095	847	947	0.896269	1154	1557	0.760895
154	287	0.688956	847	1274	0.873271	1154	1576	0.778093
154	668	0.653527	852	910	0.717646	1154	1664	0.842958
154	669	0.659209	856	972	0.625581	1155	1485	0.678847
154	1620	0.73908	856	1025	0.87104	1155	1543	0.679733
157	1496	0.691254	857	1386	0.732733	1158	1396	0.689188
168	231	0.686665	857	1459	0.746797	1165	1405	0.89004
176	426	0.64484	857	1462	0.715365	1166	1342	0.692831
176	557	0.625887	860	1009	0.894523	1176	1221	0.637045
192	672	0.61822	860	1564	0.999985	1180	1192	0.651516
192	965	0.648492	860	1669	1	1180	1595	0.699603
192	967	0.647867	861	1502	0.740014	1192	1200	0.694941
192	969	0.643293	861	1664	0.705593	1192	1397	0.708379
192	970	0.662853	864	1052	0.681082	1192	1524	0.700787
206	210	0.697755	864	1496	0.763593	1195	1300	0.775861
206	287	0.678756	864	1502	0.740159	1195	1573	0.740635
206	668	0.728777	869	1339	0.999532	1196	1321	0.720402
210	1176	0.664184	869	1423	0.999138	1196	1322	0.808076
211	736	0.697775	869	1574	0.689848	1196	1659	0.705957
211	1299	0.747215	872	1662	0.831538	1201	1223	0.820556
211	1300	0.728464	875	917	0.770939	1201	1259	0.778014
211	1349	0.761668	875	1534	0.849531	1201	1315	0.702754
213	215	0.711036	877	888	0.708396	1201	1330	0.685179
215	219	0.668886	877	1057	0.76863	1201	1382	0.790866
215	736	0.751205	877	1098	0.673459	1201	1384	0.692967
215	1300	0.729613	877	1123	0.664401	1201	1411	0.756048
217	222	0.794238	877	1246	0.651409	1201	1518	0.736342
217	1620	0.720366	877	1261	0.749747	1201	1644	0.685221
222	1637	0.684746	877	1304	0.78961	1201	1656	0.731127
235	1659	0.712883	877	1364	0.745453	1201	1664	0.769415
250	361	0.683187	877	1371	0.761191	1202	1316	0.672097
250	563	0.627235	877	1499	0.632913	1203	1317	0.745352
250	825	0.730435	877	1546	0.631674	1205	1469	0.65164
250	1375	0.734272	878	1248	0.804066	1207	1387	0.735758
252	1255	0.741239	879	917	0.705307	1207	1556	0.818307
252	1277	0.629257	879	1054	0.703234	1217	1272	0.731063
253	265	0.646166	879	1109	0.65115	1223	1259	0.919705
265	334	0.668201	879	1182	0.743313	1223	1304	0.759916
275	1011	0.815671	879	1287	0.675644	1223	1315	0.665853
275	1452	0.744224	880	1111	0.777622	1223	1330	0.811385
278	1432	0.700297	880	1144	0.722428	1223	1364	0.786083

286	867	0.711355
286	1272	0.753953
287	668	0.774668
287	669	0.75016
323	418	0.661419
323	567	0.722709
339	1397	0.655879
348	1223	0.749757
348	1259	0.748888
348	1382	0.778141
348	1451	0.704938
348	1576	0.691367
350	968	0.806252
358	1259	0.721502
358	1382	0.710833
363	702	0.668213
363	1223	0.768587
363	1259	0.78416
363	1382	0.789512
363	1386	0.718616
363	1514	0.693581
363	1576	0.704345
389	881	0.658833
399	1429	0.69049
427	493	0.706147
494	1176	0.697304
522	523	0.743722
563	881	0.633082
563	906	0.77986
567	860	0.667611
567	1011	0.740366
567	1669	0.667611
568	1319	0.693438
582	1235	0.667116
616	629	0.68262
616	702	0.728205
616	771	0.790488
616	803	0.778462
616	1000	0.721987
616	1053	0.757368
616	1054	0.756684
616	1135	0.651199
616	1223	0.760205
616	1259	0.81565
616	1382	0.799743
616	1384	0.685214
616	1397	0.638011
616	1431	0.763066
616	1451	0.793103
616	1514	0.907834
616	1576	0.860133
622	1502	0.725968
624	681	0.9156
624	809	0.806842
881	1375	0.676949
883	1002	0.791286
886	1050	0.696622
886	1595	0.837396
888	889	0.897999
888	959	0.705177
888	1027	0.790846
888	1057	0.942935
888	1098	0.905136
888	1154	0.88097
888	1223	0.767512
888	1259	0.729065
888	1272	0.67595
888	1304	0.95838
888	1346	0.628956
888	1364	0.881862
888	1382	0.792775
888	1384	0.682789
888	1451	0.732976
888	1499	0.793687
888	1524	0.740327
888	1546	0.806421
888	1557	0.896956
888	1576	0.8466
888	1644	0.681546
888	1664	0.847637
889	1027	0.855977
889	1057	0.966443
889	1098	0.998708
889	1154	0.762009
889	1223	0.77613
889	1259	0.717651
889	1304	0.93612
889	1364	0.902954
889	1382	0.80145
889	1499	0.965708
889	1524	0.797277
889	1546	0.973068
889	1557	0.999286
889	1664	0.83408
890	1315	0.648308
891	1427	0.793916
892	897	0.658533
892	1046	0.694105
892	1154	0.724599
892	1201	0.748028
892	1259	0.718648
892	1314	0.771806
892	1315	0.677256
892	1323	0.718341
892	1324	0.771791
892	1346	0.699585
892	1364	0.676756
892	1371	0.664715
1223	1382	0.972262
1223	1384	0.738973
1223	1408	0.616499
1223	1411	0.792137
1223	1451	0.827948
1223	1499	0.75804
1223	1514	0.793337
1223	1518	0.774795
1223	1546	0.761817
1223	1557	0.775132
1223	1576	0.839389
1223	1644	0.769438
1223	1656	0.769576
1223	1664	0.748135
1225	1260	0.759568
1225	1377	0.651473
1226	1418	0.731841
1226	1513	0.697666
1226	1519	0.739067
1227	1360	0.628639
1240	1489	0.753752
1241	1489	0.789621
1246	1261	0.708886
1246	1304	0.67813
1246	1331	0.835948
1246	1364	0.731812
1246	1367	0.776858
1246	1371	0.785326
1246	1432	0.661539
1246	1455	0.741219
1246	1499	0.748808
1246	1546	0.736436
1246	1558	0.806887
1248	1342	0.676645
1248	1404	0.702154
1248	1408	0.824508
1248	1438	0.744268
1248	1655	0.738691
1248	1670	0.587438
1252	1323	0.819545
1252	1655	0.706283
1255	1277	0.765243
1256	1386	0.720955
1256	1411	0.691516
1256	1578	0.708302
1256	1651	0.871299
1256	1656	0.730766
1259	1330	0.746805
1259	1344	0.756079
1259	1382	0.963304
1259	1384	0.752865
1259	1386	0.828981
1259	1411	0.815993
1259	1436	0.737544

624	864	0.668641	892	1496	0.677012	1259	1451	0.84865
629	652	0.741483	892	1664	0.746273	1259	1499	0.698134
629	738	0.808647	893	1319	0.692552	1259	1514	0.806476
629	803	0.727818	897	1346	0.649487	1259	1518	0.808935
629	845	0.718481	897	1380	0.680652	1259	1546	0.702262
629	974	0.738454	898	1477	0.717171	1259	1557	0.718047
629	1027	0.745995	898	1478	0.640556	1259	1576	0.841308
629	1045	0.694411	904	1441	0.688717	1259	1652	0.712489
629	1050	0.812721	906	1180	0.637748	1259	1656	0.802647
629	1054	0.807997	906	1202	0.676991	1259	1664	0.696009
629	1055	0.695595	906	1316	0.666481	1261	1304	0.64757
629	1135	0.776803	906	1317	0.685402	1261	1331	0.605426
629	1223	0.731958	911	1019	0.727973	1261	1371	0.850937
629	1259	0.75866	911	1055	0.737294	1261	1432	0.714734
629	1382	0.758221	911	1201	0.701401	1262	1547	0.99616
629	1502	0.723951	911	1223	0.730635	1272	1384	0.75723
629	1652	0.773976	911	1330	0.697404	1272	1397	0.79831
632	1176	0.652423	911	1408	0.771066	1272	1451	0.676041
634	978	0.6738	914	1048	0.676144	1272	1524	0.816966
637	768	0.705881	915	983	0.744895	1272	1651	0.745253
637	776	0.711414	915	1362	0.759784	1274	1306	0.724336
637	1161	0.938875	915	1367	0.732765	1277	1312	0.651802
652	767	0.781721	917	1095	0.731679	1294	1419	0.726441
652	974	0.705204	917	1182	0.694748	1299	1300	0.867658
652	1368	0.714354	917	1502	0.746912	1299	1349	0.871321
652	1652	0.712408	917	1534	0.756448	1300	1349	0.81902
653	817	0.724207	920	1496	0.641227	1304	1364	0.926876
653	1057	0.670591	922	1055	0.700372	1304	1371	0.698833
653	1304	0.677818	925	1009	0.840464	1304	1382	0.756157
653	1371	0.669675	925	1394	0.760223	1304	1499	0.872201
664	1524	0.698807	927	1019	0.837965	1304	1524	0.741593
668	669	0.824256	927	1055	0.743386	1304	1546	0.879848
669	1176	0.654254	927	1330	0.654416	1304	1557	0.935189
674	1299	0.75198	927	1408	0.868513	1304	1558	0.627398
674	1300	0.661886	934	954	0.733455	1304	1658	0.703337
674	1349	0.678645	945	1011	0.849602	1304	1664	0.84788
674	1573	0.681539	946	1503	0.828891	1314	1323	0.789732
677	832	0.636717	947	1274	0.946039	1315	1323	0.758726
677	1155	0.732316	950	1360	0.737218	1315	1364	0.797982
677	1543	0.685199	955	1109	0.639561	1315	1367	0.667694
679	680	0.7282	958	983	0.72409	1315	1371	0.743689
679	681	0.704858	958	1013	0.96862	1315	1496	0.667115
679	725	0.742594	958	1057	0.640741	1315	1644	0.680837
679	733	0.726288	958	1098	0.72562	1315	1658	0.694601
679	769	0.685508	958	1246	0.741219	1315	1661	0.727623
679	1195	0.787068	958	1331	0.928163	1315	1664	0.757402
680	1195	0.834446	958	1364	0.673152	1317	1325	0.638441
681	725	0.645361	958	1367	0.839072	1321	1397	0.754394
681	733	0.717295	958	1455	1	1321	1489	0.920804
682	688	0.853958	958	1499	0.874672	1322	1529	0.755959
682	1006	0.678492	958	1546	0.861154	1322	1659	0.780141
685	965	0.797372	958	1558	0.988809	1323	1432	0.650837
687	967	0.737696	959	1223	0.788333	1323	1496	0.657874
687	970	0.703878	959	1259	0.720008	1323	1655	0.717645

688	690	0.756611
689	706	0.699012
689	708	0.776616
689	715	0.780351
689	716	0.808838
692	880	0.733064
694	703	0.694597
694	841	0.691504
695	804	0.802378
696	711	0.662042
696	1008	0.675243
701	702	0.83664
701	888	0.684365
701	889	0.758904
701	959	0.683022
701	1057	0.731583
701	1098	0.760324
701	1199	0.658167
701	1223	0.703731
701	1259	0.710432
701	1304	0.698776
701	1382	0.728827
701	1499	0.766155
701	1546	0.767883
701	1557	0.758077
702	888	0.756993
702	889	0.797732
702	894	0.672439
702	897	0.669366
702	959	0.730639
702	1027	0.78984
702	1057	0.751696
702	1098	0.792476
702	1223	0.772787
702	1259	0.848808
702	1382	0.832383
702	1451	0.758512
702	1499	0.764419
702	1546	0.771646
702	1557	0.798245
702	1576	0.736713
703	841	0.756919
703	1135	0.726235
703	1386	0.715794
703	1656	0.714665
706	716	0.70289
708	715	0.783221
708	716	0.740601
715	716	0.766384
720	781	0.920124
720	860	0.992304
720	1073	0.82676
720	1564	0.992234
720	1669	0.992304
959	1382	0.770237
959	1513	0.681937
959	1644	0.691359
960	1063	0.68945
964	1055	0.664513
967	970	0.72096
972	974	0.700655
972	1368	0.729535
973	974	0.717424
973	1294	0.778764
974	1063	0.71609
974	1082	0.684159
974	1368	0.728241
974	1386	0.731292
977	1250	0.636357
982	1293	0.999556
983	1013	0.797052
983	1246	0.727561
983	1315	0.680772
983	1324	0.808734
983	1331	0.827215
983	1362	0.984403
983	1364	0.723234
983	1367	0.982704
983	1371	0.672963
983	1455	0.72409
983	1558	0.773419
983	1664	0.767205
990	1046	0.724272
1000	1050	0.717331
1000	1054	0.740942
1000	1094	0.739161
1000	1431	0.74629
1000	1485	0.731092
1000	1514	0.722443
1001	1212	0.637087
1002	1011	0.785486
1002	1201	0.737017
1002	1386	0.774457
1002	1411	0.765111
1002	1518	0.786224
1002	1656	0.780556
1008	1052	0.679935
1008	1226	0.685337
1008	1346	0.665815
1008	1496	0.664692
1009	1394	0.73081
1009	1564	0.894408
1009	1669	0.894523
1011	1073	0.791309
1011	1359	0.810244
1011	1452	0.854397
1013	1057	0.737238
1013	1098	0.770891
1323	1661	0.66805
1324	1346	0.724795
1324	1362	0.813913
1324	1367	0.706512
1324	1664	0.652214
1330	1382	0.787711
1330	1397	0.771746
1330	1408	0.709481
1330	1451	0.696691
1330	1459	0.691459
1330	1576	0.690168
1330	1658	0.756361
1331	1367	0.903374
1331	1371	0.712329
1331	1455	0.928163
1331	1499	0.810435
1331	1546	0.792547
1331	1558	0.962306
1334	1454	0.84957
1339	1423	0.99994
1339	1574	0.677524
1342	1513	0.714379
1342	1566	0.693594
1342	1670	0.697155
1343	1459	0.738273
1343	1513	0.721516
1343	1535	0.706421
1344	1382	0.725419
1344	1386	0.730352
1344	1411	0.71366
1344	1436	0.781496
1344	1543	0.653488
1346	1496	0.701512
1349	1519	0.750066
1362	1367	0.968229
1364	1367	0.754386
1364	1371	0.748772
1364	1382	0.748914
1364	1455	0.673152
1364	1499	0.892826
1364	1524	0.800336
1364	1546	0.89474
1364	1557	0.902042
1364	1558	0.73569
1364	1644	0.718667
1364	1658	0.776929
1364	1664	0.924363
1367	1371	0.683893
1367	1455	0.839072
1367	1499	0.758033
1367	1546	0.742721
1367	1558	0.8763
1367	1664	0.773086
1371	1432	0.688516

724	1169	0.705738	1013	1246	0.841184	1371	1558	0.654682
725	736	0.706199	1013	1304	0.670024	1374	1386	0.70693
725	769	0.769038	1013	1331	0.973226	1375	1651	0.682942
725	964	0.689745	1013	1364	0.768769	1380	1384	0.68816
725	1055	0.785789	1013	1367	0.890408	1380	1432	0.791974
725	1225	0.711852	1013	1371	0.720831	1380	1451	0.72443
725	1395	0.670477	1013	1455	0.96862	1382	1384	0.817422
730	898	0.906256	1013	1499	0.897601	1382	1386	0.784634
730	1478	0.61434	1013	1546	0.883328	1382	1397	0.718232
733	1161	0.70562	1013	1558	0.99486	1382	1411	0.817578
736	1299	0.785346	1013	1664	0.701464	1382	1436	0.707893
736	1300	0.814046	1015	1088	0.714539	1382	1451	0.903429
736	1349	0.862602	1018	1026	0.83024	1382	1499	0.775482
738	790	0.700544	1018	1166	0.763354	1382	1514	0.822368
738	1050	0.854543	1018	1248	0.806418	1382	1518	0.801576
738	1054	0.789036	1018	1404	0.765088	1382	1546	0.781037
738	1496	0.677066	1018	1655	0.751077	1382	1557	0.802285
744	760	0.828093	1018	1670	0.839472	1382	1576	0.867573
745	1319	0.729674	1019	1055	0.797325	1382	1644	0.716799
764	802	0.696133	1019	1201	0.645159	1382	1651	0.681113
767	925	0.762357	1019	1223	0.767344	1382	1652	0.71815
767	972	0.699026	1019	1315	0.711518	1382	1656	0.806951
767	1009	0.867758	1019	1330	0.709098	1382	1664	0.745074
767	1085	0.708876	1019	1364	0.691997	1384	1397	0.749365
767	1368	0.702109	1019	1408	0.754804	1384	1432	0.674504
767	1394	0.704645	1019	1595	0.708708	1384	1451	0.868686
768	1560	0.70216	1019	1644	0.849431	1384	1576	0.679201
770	1023	0.726407	1023	1322	0.938141	1384	1651	0.735367
770	1322	0.745773	1023	1529	0.820855	1384	1656	0.679217
774	1192	0.641707	1023	1659	0.785929	1386	1411	0.959664
774	1217	0.76023	1025	1073	0.783166	1386	1436	0.734169
774	1272	0.757282	1025	1255	0.822357	1386	1459	0.714421
774	1397	0.797405	1025	1344	0.747584	1386	1513	0.768789
776	832	0.687755	1025	1436	0.752419	1386	1518	0.978396
776	857	0.787726	1025	1454	0.809006	1386	1652	0.759223
776	1161	0.735679	1026	1314	0.72065	1386	1656	0.970782
782	1535	0.713944	1026	1323	0.810813	1391	1652	0.70423
787	927	0.678125	1026	1670	0.66523	1395	1502	0.742984
787	1364	0.702648	1027	1057	0.824469	1397	1404	0.721295
795	904	0.657306	1027	1098	0.854257	1397	1432	0.715445
795	1317	0.682846	1027	1223	0.80135	1397	1451	0.74165
802	804	0.839673	1027	1259	0.807401	1397	1651	0.736031
803	1000	0.75462	1027	1304	0.797894	1397	1655	0.755729
803	1053	0.656271	1027	1364	0.76945	1400	1513	0.712267
803	1054	0.715369	1027	1382	0.834998	1400	1655	0.689489
803	1223	0.748113	1027	1451	0.689903	1404	1655	0.897608
803	1259	0.809351	1027	1499	0.825765	1411	1436	0.716172
803	1382	0.781811	1027	1502	0.742907	1411	1513	0.737632
803	1384	0.665149	1027	1546	0.832261	1411	1518	0.978404
803	1431	0.845938	1027	1557	0.85484	1411	1652	0.78393
803	1451	0.817446	1027	1576	0.745769	1411	1656	0.976655
803	1514	0.852687	1027	1664	0.769234	1418	1519	0.810183
803	1576	0.762513	1044	1208	0.667493	1423	1574	0.672883
806	869	0.998944	1045	1055	0.702253	1429	1552	0.708975

806	1339	0.997074	1045	1095	0.720815	1431	1514	0.833109
806	1423	0.996181	1045	1135	0.704257	1431	1576	0.758858
806	1574	0.70684	1045	1436	0.798917	1432	1655	0.706682
807	857	0.758733	1045	1496	0.789644	1436	1459	0.707459
807	1386	0.726662	1045	1661	0.659307	1436	1462	0.74356
811	914	0.648551	1046	1055	0.694852	1436	1496	0.696086
811	1386	0.707911	1046	1088	0.727428	1436	1518	0.750908
812	1002	0.786224	1046	1169	0.681989	1436	1576	0.65355
812	1201	0.736342	1046	1223	0.699463	1436	1652	0.687449
812	1223	0.774795	1046	1315	0.684624	1436	1656	0.736096
812	1259	0.808935	1046	1371	0.735006	1451	1514	0.837537
812	1382	0.801576	1048	1192	0.677286	1451	1576	0.825779
812	1386	0.978396	1048	1489	0.610036	1451	1651	0.696925
812	1411	0.978404	1050	1054	0.866585	1455	1499	0.874672
812	1436	0.750908	1050	1595	0.732691	1455	1546	0.861154
812	1513	0.734507	1052	1346	0.773661	1455	1558	0.988809
812	1518	1	1052	1496	0.740665	1485	1543	0.71812
812	1652	0.763108	1052	1502	0.784632	1487	1498	0.766053
812	1656	0.993023	1052	1661	0.742915	1496	1502	0.680565
815	883	0.819792	1053	1223	0.724779	1496	1661	0.711261
817	846	0.759723	1053	1382	0.711684	1496	1664	0.635453
817	877	0.786566	1053	1514	0.755662	1499	1524	0.815788
817	888	0.736054	1053	1576	0.74648	1499	1546	0.999508
817	958	0.619952	1054	1055	0.698996	1499	1557	0.964952
817	983	0.718303	1054	1109	0.732502	1499	1558	0.895121
817	1013	0.771593	1054	1135	0.703644	1499	1664	0.822613
817	1057	0.812875	1054	1514	0.758837	1502	1534	0.807912
817	1098	0.734584	1054	1534	0.835972	1502	1664	0.793201
817	1123	0.644876	1055	1095	0.709158	1503	1561	0.806397
817	1154	0.726006	1055	1135	0.774973	1503	1572	0.808067
817	1223	0.704803	1055	1223	0.784336	1513	1518	0.734507
817	1246	0.813601	1055	1225	0.77072	1513	1656	0.722949
817	1259	0.70401	1055	1315	0.721255	1513	1670	0.656971
817	1261	0.829858	1055	1330	0.73706	1514	1576	0.933499
817	1304	0.811985	1055	1364	0.711804	1514	1595	0.756741
817	1331	0.786292	1055	1382	0.726367	1518	1652	0.763108
817	1364	0.782599	1055	1397	0.683614	1518	1656	0.993023
817	1367	0.738716	1055	1400	0.715138	1524	1546	0.816046
817	1371	0.879888	1055	1408	0.736054	1524	1557	0.796066
817	1382	0.705808	1057	1098	0.977033	1524	1664	0.77849
817	1432	0.738085	1057	1154	0.805649	1546	1557	0.972322
817	1455	0.619952	1057	1223	0.777604	1546	1558	0.881047
817	1499	0.748938	1057	1246	0.707517	1546	1664	0.824861
817	1546	0.741549	1057	1261	0.625627	1557	1664	0.832931
817	1558	0.71571	1057	1304	0.993197	1558	1664	0.673532
817	1664	0.757022	1057	1364	0.939927	1564	1669	0.999985
818	1047	0.696396	1057	1367	0.630097	1578	1655	0.713741
819	857	0.796971	1057	1371	0.691619	1595	1644	0.717582
819	1459	0.771309	1057	1382	0.779439	1618	1619	0.916235
824	983	0.8976	1057	1455	0.640741	1644	1664	0.685279
824	1324	0.749273	1057	1499	0.92312	1651	1656	0.725362
824	1362	0.914368	1057	1524	0.776622	1652	1656	0.752839
824	1367	0.882933	1057	1546	0.929201	1655	1670	0.677681
824	1524	0.733534	1057	1557	0.965537	1658	1664	0.722336

825	881	0.702027	1057	1558	0.703559	1661	1664	0.677431

Class IV

AA1	AA2	Correlation coefficient	AA1	AA2	Correlation coefficient	AA1	AA2	Correlation coefficient
2	20	0.470608	293	776	0.671878	648	920	0.569647
2	44	0.79055	293	1193	0.658298	648	930	0.912468
4	17	0.621704	305	417	0.564158	648	1055	0.535589
4	18	0.684087	311	433	0.62424	648	1198	0.550398
4	61	0.612399	311	637	0.619315	649	653	0.609913
4	575	0.517644	311	947	0.695602	650	657	0.727969
4	1007	0.566678	311	950	0.613773	650	1151	0.632603
4	1111	0.82282	325	864	0.532136	656	727	0.531475
17	18	0.59449	326	403	0.579986	657	1151	0.819439
17	19	0.485955	329	330	0.564584	658	980	0.490752
17	21	0.705084	330	585	0.514837	661	732	0.7091
17	61	0.990162	334	576	0.463568	663	671	0.518341
17	179	0.463778	349	668	0.51966	663	930	0.614388
17	575	0.615947	349	925	0.545276	665	687	0.583864
17	1111	0.427419	349	1246	0.635104	666	668	0.521006
18	19	0.566457	350	379	0.513792	666	669	0.499549
18	44	0.558553	351	600	0.540249	668	730	0.512054
18	61	0.557151	359	925	0.541576	668	925	0.495844
18	1111	0.567466	360	362	0.492809	668	1111	0.502073
20	43	0.43625	366	776	0.517133	668	1202	0.550469
20	44	0.500621	367	668	0.522474	668	1246	0.504981
21	61	0.698977	367	1093	0.503711	669	863	0.551929
44	84	0.432665	368	1218	0.485287	669	1211	0.503221
61	179	0.450332	368	1260	0.471061	671	938	0.516842
61	575	0.603551	376	619	0.556866	674	1067	0.530501
65	147	0.473448	380	597	0.627784	675	678	0.477929
65	217	0.508172	380	600	0.567887	687	1091	0.534313
65	1235	0.49019	380	628	0.685772	689	825	0.549624
83	86	0.824305	380	876	0.510482	701	890	0.565634
85	86	0.664397	381	665	0.769889	708	755	0.482879
90	100	0.451926	389	1181	0.600393	714	718	0.599255
92	94	0.4568	390	1222	0.570909	714	785	0.589443
92	170	0.467853	394	614	0.54158	714	1085	0.637298
93	98	0.573581	394	789	0.512279	714	1090	0.758775
94	97	0.524665	394	1110	0.490468	714	1101	0.574876
94	98	0.694692	398	499	0.753697	716	820	0.628196
94	107	0.503322	398	969	0.624844	727	729	0.521393
94	115	0.495764	398	1231	0.542995	729	789	0.516244

94	177	0.430257	403	641	0.513409	729	805	0.488386
94	363	0.448039	403	738	0.709074	730	764	0.528522
94	576	0.488129	403	739	0.644743	730	791	0.492655
96	668	0.497017	403	791	0.567585	730	795	0.541264
96	1077	0.563975	403	795	0.672759	730	868	0.540165
96	1189	0.643637	403	816	0.567665	730	924	0.511208
96	1202	0.522694	403	871	0.596055	730	1067	0.631367
97	1247	0.531221	403	970	0.56722	730	1195	0.529268
98	99	0.612401	403	1262	0.716896	738	739	0.793147
98	107	0.618682	404	417	0.561817	738	740	0.574513
115	132	0.516352	406	455	0.551461	738	791	0.545311
115	135	0.524699	406	587	0.565029	738	795	0.599987
115	136	0.501533	406	661	0.528564	738	868	0.531853
115	170	0.451495	407	1219	0.560055	738	1198	0.542042
116	124	0.561096	408	657	0.55957	738	1262	0.542446
116	743	0.505	409	738	0.526084	739	740	0.646453
124	153	0.486983	413	666	0.539225	739	823	0.495418
124	154	0.451436	419	544	0.613114	739	1198	0.502625
124	361	0.487287	419	1154	0.551601	749	815	0.508149
124	401	0.549308	422	427	0.586042	750	940	0.583333
132	135	0.569474	422	821	0.500781	752	875	0.626314
132	136	0.571934	422	1100	0.527132	753	760	0.515202
132	139	0.572499	422	1101	0.540373	753	923	0.658903
132	145	0.475488	422	1102	0.538143	753	985	0.487589
132	146	0.482756	424	951	0.605929	764	805	0.496363
132	150	0.466782	425	578	0.492563	764	924	0.499372
132	180	0.530435	425	1139	0.546422	776	791	0.466288
132	1106	0.542318	430	628	0.760748	776	923	0.712843
135	136	0.717703	433	725	0.475525	776	1193	0.599105
135	137	0.618599	433	1218	0.512054	785	1181	0.647493
135	138	0.558769	439	499	0.841493	789	805	0.52573
135	139	0.48541	439	919	0.556183	789	807	0.513775
135	144	0.540271	439	1055	0.464748	789	932	0.531182
135	145	0.492282	439	1181	0.646759	791	1120	0.536115
135	147	0.485119	441	547	0.537219	791	1262	0.564879
135	150	0.558174	441	733	0.766877	795	871	0.789442
135	170	0.522248	446	778	0.560672	795	1262	0.533149
135	362	0.524105	452	495	0.588497	800	805	0.513684
135	367	0.516025	453	529	0.499563	801	882	0.603213
135	518	0.547111	456	822	0.577416	805	807	0.505994
135	619	0.490086	456	917	0.555593	815	930	0.545756
135	637	0.503383	456	1201	0.735977	815	1212	0.538815
135	1151	0.469331	457	463	0.51749	816	823	0.502015
135	1231	0.493292	458	476	0.77272	816	1262	0.679526

136	137	0.471795	458	480	0.559735	820	916	0.861867
136	150	0.61812	461	1201	0.662652	822	823	0.766661
136	154	0.471959	462	899	0.526825	822	930	0.804678
136	352	0.496057	463	825	0.666948	822	1198	0.50362
136	362	0.43658	465	1093	0.545472	822	1201	0.781241
136	690	0.490821	472	476	0.624381	823	930	0.632771
137	138	0.538568	474	947	0.52039	823	1046	0.58911
137	144	0.535771	476	480	0.548295	823	1201	0.765414
137	669	0.467509	480	947	0.712248	828	875	0.570205
137	1231	0.566046	498	977	0.540856	862	870	0.74469
138	139	0.504684	498	1198	0.594678	866	980	0.495213
138	145	0.501556	499	1181	0.659871	867	870	0.503229
138	147	0.555911	500	1157	0.615099	867	872	0.617989
138	150	0.47816	500	1219	0.587228	867	1262	0.434195
139	145	0.523149	501	661	0.57667	868	924	0.544307
139	147	0.540533	505	968	0.749298	870	872	0.728355
143	148	0.575939	506	1166	0.573704	871	1198	0.502154
144	147	0.462952	509	893	0.666824	871	1207	0.498385
144	359	0.543045	510	871	0.55127	887	977	0.616256
144	388	0.501949	516	517	0.500643	887	1151	0.657773
144	598	0.600191	518	609	0.672051	888	895	0.875719
144	1089	0.444557	518	614	0.526732	890	1133	0.51008
145	146	0.470432	518	730	0.529178	890	1139	0.646632
145	147	0.661538	518	970	0.535647	890	1252	0.533698
145	148	0.491892	519	748	0.535519	891	976	0.570384
145	177	0.513955	519	749	0.476544	891	985	0.897837
145	1088	0.491632	519	961	0.587829	899	914	0.716803
145	1090	0.445425	522	899	0.567782	920	930	0.616234
146	751	0.46853	527	600	0.552291	925	1054	0.534695
146	805	0.57044	532	533	0.645345	925	1111	0.48764
147	150	0.490561	532	592	0.646713	930	1055	0.524883
149	601	0.568386	532	647	0.692905	946	985	0.657104
149	1089	0.589121	532	981	0.563802	947	1189	0.653226
150	352	0.557061	533	647	0.899543	947	1231	0.548817
153	361	0.501538	533	1231	0.507486	951	1176	0.490957
154	177	0.455973	533	1246	0.733829	963	1042	0.573868
154	362	0.453077	544	653	0.712149	963	1148	0.762814
155	916	0.58033	545	630	0.630581	964	1111	0.455942
176	755	0.501298	547	576	0.520546	974	1250	0.467909
178	324	0.479549	547	733	0.573705	976	985	0.721564
178	380	0.512913	550	567	0.545702	984	1181	0.567889
180	899	0.56673	566	1250	0.582273	1007	1111	0.632791
180	914	0.572503	569	648	0.662162	1045	1047	0.683802
185	186	0.522585	569	930	0.690797	1045	1056	0.558005

186	259	0.623	575	1111	0.434493	1045	1061	0.582157
188	274	0.586364	585	1139	0.675844	1046	1201	0.684089
196	940	0.695469	587	592	0.685529	1047	1056	0.695623
196	1043	0.639648	587	658	0.614609	1047	1060	0.58307
196	1224	0.558243	588	637	0.70406	1047	1061	0.756315
199	323	0.519355	588	648	0.415085	1047	1062	0.649783
199	514	0.482319	588	649	0.817309	1049	1051	0.521815
200	1173	0.757707	588	653	0.57297	1049	1151	0.610304
203	600	0.644278	588	707	0.723181	1050	1053	0.48382
203	795	0.702422	588	947	0.611449	1051	1061	0.531294
203	871	0.530789	588	1231	0.519794	1051	1062	0.531059
204	262	0.52135	589	593	0.553558	1051	1195	0.59321
204	405	0.538715	590	635	0.526209	1054	1093	0.539943
207	263	0.678302	596	751	0.626917	1056	1059	0.521117
207	264	0.740695	597	628	0.540284	1056	1060	0.73284
208	210	0.560476	599	628	0.582742	1056	1061	0.885159
210	456	0.491961	600	615	0.525961	1056	1062	0.871367
210	1067	0.542032	600	645	0.589438	1059	1061	0.604254
217	368	0.495355	600	795	0.595803	1060	1061	0.743458
217	609	0.49421	600	876	0.481332	1060	1062	0.736603
217	656	0.449207	601	1089	0.535041	1061	1062	0.853582
217	1093	0.543598	605	705	0.533833	1067	1081	0.488964
218	650	0.509009	605	764	0.505089	1067	1195	0.642077
218	925	0.474151	609	610	0.786438	1078	1165	0.56087
219	545	0.522594	609	620	0.628857	1082	1252	0.566808
219	730	0.48134	609	1246	0.62276	1085	1087	0.656594
224	758	0.501534	610	620	0.586726	1085	1088	0.621079
224	1068	0.606686	613	614	0.530915	1085	1089	0.574118
224	1252	0.521691	613	789	0.479361	1085	1090	0.717438
225	268	0.629219	613	820	0.518843	1085	1091	0.591972
227	259	0.564383	613	916	0.74064	1085	1094	0.610748
259	397	0.46659	614	616	0.535332	1085	1101	0.720215
262	273	0.551878	614	732	0.61619	1085	1102	0.749499
262	792	0.561493	615	649	0.517614	1087	1088	0.572851
262	964	0.503175	619	1231	0.592214	1087	1091	0.685226
262	1111	0.518834	627	914	0.537631	1087	1094	0.716489
264	267	0.494935	637	644	0.576323	1087	1101	0.541595
267	390	0.511885	637	649	0.680984	1087	1102	0.537942
267	1111	0.551003	637	947	0.702313	1088	1091	0.652221
268	732	0.559193	637	950	0.567137	1088	1094	0.498199
269	1049	0.579163	637	1231	0.643062	1088	1101	0.732731
270	984	0.540879	640	1106	0.588464	1088	1102	0.688807
272	739	0.508897	641	730	0.571257	1089	1090	0.602782
273	394	0.501186	641	764	0.494748	1090	1101	0.608724

273	792	0.643995	641	1080	0.484532	1090	1102	0.611816
277	281	0.666672	644	649	0.637371	1091	1094	0.624024
278	333	0.501945	645	795	0.49205	1099	1101	0.505302
283	701	0.658797	645	1211	0.53337	1100	1101	0.683938
288	386	0.557299	648	649	0.495659	1100	1102	0.597282
289	822	0.571406	648	663	0.635171	1101	1102	0.905894
293	326	0.592161	648	822	0.789412	1147	1173	0.567588
293	437	0.709764	648	823	0.63837	1224	1225	0.481974

(B) Summary of coevolving amino acid pairs in NaAGO1a, NaAGO5, NaAGO2 and NaAGO4a, representatives of Class I – IV, respectively.

	Number of coevolving pairs	Number of coevolving residues	Number of coevolving residues in different domains			
			N	PAZ	MID	PIWI
NaAGO1a	561	272	35	27	36	60
NaAGO5	367	292	43	47	37	77
NaAGO2	1005	369	44	52	47	102
NaAGO4a	481	366	50	47	53	100

(B) Frequency of coevolving amino acid (AA) pairs in NaAGO1a, NaAGO5, NaAGO2 and NaAGO4a.

Amino acid pairs	NaAGO1a	NaAGO5	NaAGO2	NaAGO4a
RR	9	7	3	3
RK	3	0	6	0
RD	3	1	7	0
RE	1	1	4	4
RN	4	1	8	3
RC	0	0	1	1
RQ	12	1	3	0
RH	5	2	2	1
RS	3	7	3	3
RT	3	2	5	2
RY	0	4	1	1
RA	7	2	5	2
RG	21	1	3	0
RI	2	3	2	7
RL	3	2	6	3
RM	3	4	2	0

RF	5	1	8	2
RP	5	3	4	1
RW	0	0	2	0
RV	4	1	9	5
KR	2	3	1	3
KK	0	2	6	3
KD	3	2	1	2
KE	2	1	4	4
KN	1	1	6	5
KC	0	0	0	1
KQ	3	1	2	0
KH	0	0	2	1
KS	2	3	3	3
KT	0	1	2	0
KY	0	1	6	0
KA	3	3	4	1
KG	0	2	4	0
KI	0	1	5	5
KL	2	3	13	5
KM	0	1	2	2
KF	0	1	5	0
KP	0	0	3	1
KW	0	0	2	1
KV	2	4	13	1
DR	2	2	2	1
DK	1	1	2	2
DD	1	0	7	0
DE	1	2	1	2
DN	1	0	0	0
DC	0	0	0	0
DQ	3	0	1	0
DH	2	0	0	0
DS	2	1	0	1
DT	0	1	0	3
DY	0	0	0	0
DA	0	0	3	1
DG	6	0	3	1
DI	1	3	0	5
DL	0	0	6	4
DM	0	0	1	0
DF	0	2	2	1
DP	1	0	1	4
DW	0	0	0	1
DV	2	3	2	0

ER	2	1	0	4
EK	1	2	1	5
ED	2	1	2	2
EE	3	0	1	3
EN	3	0	3	3
EC	0	1	0	1
EQ	5	1	2	1
EH	0	2	1	1
ES	3	0	1	6
ET	4	0	3	1
EY	0	0	1	1
EA	1	0	3	4
EG	1	0	1	2
EI	6	1	0	1
EL	2	0	4	2
EM	0	0	1	0
EF	0	1	2	1
EP	1	0	6	1
EW	0	0	1	1
EV	4	1	8	4
NR	1	2	2	2
NK	2	0	2	4
ND	1	0	2	1
NE	2	0	2	2
NN	1	0	3	1
NC	0	0	0	0
NQ	1	0	4	1
NH	0	0	2	0
NS	2	0	0	2
NT	0	0	4	0
NY	0	2	1	1
NA	0	0	2	0
NG	0	0	0	1
NI	3	0	0	1
NL	2	1	3	5
NM	0	0	1	1
NF	0	0	3	2
NP	0	1	3	0
NW	0	0	0	0
NV	2	0	3	4
CR	0	1	1	0
CK	0	0	0	1
CD	0	2	0	1
CE	0	0	0	0

CN	0	0	1	0
CC	0	0	3	0
CQ	0	0	1	0
CH	0	3	0	0
CS	0	0	0	0
CT	0	2	0	0
CY	0	0	1	0
CA	0	0	0	1
CG	0	0	0	0
CI	0	0	2	1
CL	0	0	2	0
CM	0	1	1	0
CF	0	0	2	0
CP	0	0	1	0
CW	0	0	0	1
CV	0	0	1	1
QR	5	1	1	1
QK	2	0	0	0
QD	2	1	1	0
QE	2	0	0	0
QN	2	1	0	1
QC	0	1	0	0
QQ	4	0	0	0
QH	3	4	2	0
QS	2	1	0	0
QT	3	0	1	1
QY	0	1	0	0
QA	6	0	0	1
QG	4	0	1	0
QI	3	0	0	0
QL	2	0	2	3
QM	0	1	0	0
QF	4	1	2	1
QP	1	0	3	1
QW	0	0	0	0
QV	2	2	1	1
HR	4	0	2	0
HK	1	0	2	1
HD	0	0	0	0
HE	0	1	0	0
HN	0	0	1	0
HC	0	0	0	0
HQ	3	0	1	0
HH	0	1	1	0

HS	3	0	1	0
HT	0	0	1	0
HY	1	0	1	0
HA	4	0	0	1
HG	1	2	1	0
HI	1	0	0	1
HL	1	1	1	1
HM	0	0	0	0
HF	1	0	0	0
HP	2	0	2	0
HW	0	0	0	0
HV	2	0	4	1
SR	4	3	3	3
SK	0	3	3	3
SD	3	1	3	0
SE	3	2	0	1
SN	1	0	3	2
SC	1	0	0	0
SQ	5	1	1	0
SH	0	1	0	1
SS	2	2	0	1
ST	3	1	1	0
SY	0	2	0	0
SA	5	3	4	4
SG	1	5	2	1
SI	4	1	2	4
SL	1	5	5	5
SM	0	0	0	1
SF	2	2	2	2
SP	1	1	3	1
SW	0	0	0	0
SV	1	5	7	0
TR	3	1	1	1
TK	2	0	2	0
TD	0	0	1	0
TE	2	0	1	1
TN	1	0	1	2
TC	0	1	0	0
TQ	2	2	4	0
TH	0	0	2	0
TS	3	2	2	0
TT	0	0	3	0
TY	3	0	0	1
TA	1	1	1	0

TG	2	1	0	2
TI	1	1	0	1
TL	3	1	5	1
TM	0	0	1	2
TF	1	0	1	1
TP	0	0	7	0
TW	0	0	1	0
TV	1	2	6	1
YR	2	1	1	0
YK	0	2	2	0
YD	0	2	1	0
YE	0	0	2	0
YN	0	0	2	0
YC	0	0	0	0
YQ	1	3	2	2
YH	1	1	0	0
YS	0	2	1	1
YT	0	0	0	0
YY	0	3	1	0
YA	0	3	2	0
YG	0	1	1	1
YI	0	1	1	1
YL	0	0	3	0
YM	0	1	1	0
YF	0	2	6	1
YP	1	0	5	1
YW	0	0	1	0
YV	0	2	5	1
AR	1	2	4	1
AK	1	1	2	1
AD	1	2	2	0
AE	0	3	4	0
AN	1	0	2	0
AC	0	0	2	1
AQ	1	0	2	1
AH	3	0	4	0
AS	0	4	2	6
AT	0	0	3	1
AY	0	0	2	1
AA	2	0	7	0
AG	2	0	4	3
AI	0	1	2	3
AL	2	1	6	3
AM	0	3	1	0

AF	1	1	4	0
AP	1	1	6	2
AW	0	0	0	0
AV	1	3	9	0
GR	12	3	1	1
GK	3	3	4	0
GD	1	2	2	0
GE	0	3	3	1
GN	2	2	6	0
GC	0	0	1	0
GQ	17	1	2	1
GH	8	0	1	2
GS	6	6	2	1
GT	0	0	2	0
GY	0	2	3	0
GA	7	1	4	1
GG	18	6	2	0
GI	0	2	2	3
GL	1	1	4	1
GM	3	1	3	1
GF	2	0	5	0
GP	0	1	7	0
GW	0	0	0	0
GV	4	3	8	2
IR	4	2	2	0
IK	0	1	4	1
ID	2	1	5	3
IE	3	1	2	2
IN	2	1	1	2
IC	0	0	1	0
IQ	1	0	0	0
IH	2	0	2	0
IS	1	4	2	3
IT	2	2	1	2
IY	2	0	3	1
IA	2	2	4	0
IG	1	1	2	0
II	3	3	0	4
IL	3	2	9	3
IM	0	0	0	0
IF	1	0	2	0
IP	0	1	6	2
IW	0	0	1	0
IV	3	5	9	4

LR	3	1	1	6
LK	1	2	10	4
LD	0	1	4	1
LE	2	2	3	0
LN	2	2	1	3
LC	0	0	0	0
LQ	3	0	2	0
LH	0	0	2	1
LS	0	1	2	6
LT	1	0	3	1
LY	1	2	5	0
LA	0	0	5	5
LG	0	1	1	1
LI	3	1	1	7
LL	2	1	11	7
LM	0	0	0	1
LF	2	1	8	4
LP	0	0	5	7
LW	0	0	2	0
LV	1	4	13	7
MR	1	1	0	0
MK	0	0	0	0
MD	0	0	0	0
ME	0	1	0	0
MN	0	0	2	0
MC	0	0	0	0
MQ	1	1	4	0
MH	1	0	1	0
MS	0	1	0	2
MT	0	0	2	1
MY	0	0	0	0
MA	0	1	2	0
MG	1	1	0	0
MI	0	1	0	0
ML	1	1	0	0
MM	0	1	0	0
MF	0	2	2	0
MP	0	2	3	1
MW	0	0	0	0
MV	1	1	2	0
FR	0	1	6	2
FK	2	1	7	3
FD	0	0	0	4
FE	1	0	5	1

FN	0	0	6	0
FC	0	0	2	0
FQ	1	0	5	0
FH	1	0	3	0
FS	2	1	2	6
FT	1	0	4	0
FY	0	1	5	0
FA	2	1	5	0
FG	0	1	1	1
FI	2	1	2	2
FL	2	0	7	3
FM	0	1	2	0
FF	1	1	3	2
FP	1	0	10	1
FW	0	0	2	0
FV	0	1	12	1
PR	4	1	3	8
PK	1	0	7	2
PD	1	1	3	4
PE	1	0	5	2
PN	0	0	5	2
PC	0	0	4	0
PQ	2	0	10	0
PH	1	0	3	0
PS	1	1	3	0
PT	2	1	5	2
PY	0	0	2	0
PA	3	1	5	4
PG	6	2	2	7
PI	1	0	3	2
PL	1	0	7	1
PM	1	0	0	0
PF	1	0	3	4
PP	0	1	6	16
PW	0	0	2	0
PV	1	1	10	2
WR	0	0	1	0
WK	0	0	2	0
WD	0	0	0	0
WE	0	0	1	0
WN	0	0	1	0
WC	0	0	0	0
WQ	0	0	1	0
WH	0	0	1	0

WS	0	0	0	0
WT	0	0	1	0
WY	0	0	1	0
WA	0	0	0	0
WG	0	0	0	0
WI	0	0	1	0
WL	0	0	3	0
WM	0	0	0	0
WF	0	0	2	0
WP	0	0	0	0
WW	0	0	0	0
WV	0	0	3	0
VR	4	0	4	1
VK	4	1	9	2
VD	3	3	7	3
VE	2	0	4	2
VN	0	0	3	1
VC	0	0	1	0
VQ	1	0	5	0
VH	0	0	2	0
VS	4	5	4	3
VT	2	1	6	0
VY	1	1	2	0
VA	0	1	4	3
VG	1	2	3	2
VI	3	2	1	2
VL	1	1	11	0
VM	0	1	0	0
VF	0	1	2	0
VP	1	1	6	2
VW	0	0	2	0
VV	2	3	11	4
